# Quantifying Geographic Variation in Health Care Outcomes in the United States before and after Risk-Adjustment

**DOI:** 10.1371/journal.pone.0166762

**Published:** 2016-12-14

**Authors:** Barry L. Rosenberg, Joshua A. Kellar, Anna Labno, David H. M. Matheson, Michael Ringel, Paige VonAchen, Richard I. Lesser, Yue Li, Justin B. Dimick, Atul A. Gawande, Stefan H. Larsson, Hamilton Moses

**Affiliations:** 1 The Boston Consulting Group, Boston, Massachusetts, United States of America; 2 Department of Public Health Sciences, University of Rochester Medical Center, New York City, New York, United States of America; 3 Center for Healthcare Outcomes and Policy, University of Michigan, Ann Arbor, Michigan, United States of America; 4 Department of Surgery, University of Michigan, Ann Arbor, Michigan, United States of America; 5 Ariadne Labs At Brigham and Women’s Hospital and Harvard T.H. Chan School of Public Health, Boston, Massachusetts, United States of America; 6 The Alerion Institute and Alerion Advisors, LLC, North Garden, Virginia, United States of America; 7 Johns Hopkins School of Medicine, Baltimore, Maryland, United States of America; Osaka University Graduate School of Medicine, JAPAN

## Abstract

**Background:**

Despite numerous studies of geographic variation in healthcare cost and utilization at the local, regional, and state levels across the U.S., a comprehensive characterization of geographic variation in outcomes has not been published. Our objective was to quantify variation in US health outcomes in an all-payer population before and after risk-adjustment.

**Methods and Findings:**

We used information from 16 independent data sources, including 22 million all-payer inpatient admissions from the Healthcare Cost and Utilization Project (which covers regions where 50% of the U.S. population lives) to analyze 24 inpatient mortality, inpatient safety, and prevention outcomes. We compared outcome variation at state, hospital referral region, hospital service area, county, and hospital levels. Risk-adjusted outcomes were calculated after adjusting for population factors, co-morbidities, and health system factors. Even after risk-adjustment, there exists large geographical variation in outcomes. The variation in healthcare outcomes exceeds the well publicized variation in US healthcare costs. On average, we observed a 2.1-fold difference in risk-adjusted mortality outcomes between top- and bottom-decile hospitals. For example, we observed a 2.3-fold difference for risk-adjusted acute myocardial infarction inpatient mortality. On average a 10.2-fold difference in risk-adjusted patient safety outcomes exists between top and bottom-decile hospitals, including an 18.3-fold difference for risk-adjusted Central Venous Catheter Bloodstream Infection rates. A 3.0-fold difference in prevention outcomes exists between top- and bottom-decile counties on average; including a 2.2-fold difference for risk-adjusted congestive heart failure admission rates. The population, co-morbidity, and health system factors accounted for a range of R^2^ between 18–64% of variability in mortality outcomes, 3–39% of variability in patient safety outcomes, and 22–70% of variability in prevention outcomes.

**Conclusion:**

The amount of variability in health outcomes in the U.S. is large even after accounting for differences in population, co-morbidities, and health system factors. These findings suggest that: 1) additional examination of regional and local variation in risk-adjusted outcomes should be a priority; 2) assumptions of uniform hospital quality that underpin rationale for policy choices (such as narrow insurance networks or antitrust enforcement) should be challenged; and 3) there exists substantial opportunity for outcomes improvement in the US healthcare system.

## Introduction

Geographic variation in the cost and utilization of health care within the United States has been well documented and much debated [1–16]. In 2010, a comprehensive study was commissioned from the Institute of Medicine (IOM) to investigate geographic variation in health care spending and utilization [12–14]. The IOM study demonstrated 1.7x variation between the highest cost decile Hospital Service Areas (top 10%) and the lowest cost decile Hospital Service Areas (HSAs) (bottom 10%) in the US [14]. Additionally, prior research has examined the impact of demographics and systems factors on variation in healthcare cost and utilization [4–7,11,14,17–19].

Despite the extensive analysis of geographic variation in healthcare cost and utilization across the U.S., equivalent characterization of geographic variation in outcomes has not been published. Many studies focus on variation in cost, all-cause mortality, and *process* quality measures, but geographic variation in specific health *outcomes* has not been examined comprehensively. Prior efforts have often focused on investigating the correlation between an outcome and a single factor at a national level [20]. Research on the geographic variation of health outcomes has typically been subject to a number of important limitations including: (a) focus only on the Medicare population [21–31], which has recently been shown to be poorly representative of the all-payer population [32], (b) focus on a narrow geography [24,27,33–39], often looking at only one state or region of the US, (c) analyzing only a single or few outcomes [22–26,33–37,40–43] and/or (d) using limited risk-adjustment [24,29,33,37,39,44], typically by only adjusting for demographics and co-morbidities [42].

Finally, when geographic variation in outcomes is examined, most studies analyze outcomes over large geographic areas (national [45], regional [41], state [11,22,25,30], or hospital referral region [23,28–30,43] (HRR)). This results in "over-averaging" which masks the true extent of variation [46–48]. In fact, when the IOM examined variation in spending and utilization it concluded that variation "can be explained not by HRR-level factors but by factors at the smaller, HSA geographic level." [14] Ultimately, the IOM explicitly commented that, "more research on health care outcomes and quality is needed, particularly in commercially insured populations".[14] Recently, analysis of commercially insured populations from 2007–2009 was published by McKellar and colleagues, in which 10 quality measures were examined for variation at the HRR level, including four outcomes measures, and six process measures [49].

In this context, the present study provides a comprehensive analysis of geographic variation in United States health outcomes. Our analysis is conducted at the state, HRR, HSA, county, and hospital levels, both before and after risk-adjustment. The present study uses an all-payer population, spanning the 50% of the United States for which geographically identified data is available in the Health Care Utilization Project (HCUP), to examine 24 outcomes including inpatient mortality (IQIs), patient safety (PSIs), and prevention measures (PQIs) with rigorous risk-adjustment. These findings have important implications to all health care stakeholders including patients, physicians, hospital systems, payers, policymakers, and pharmaceutical and medical technology companies.

## Methods

### Data sources and study population

We analyzed outcomes variation across the United States using 2011 inpatient administrative data from the Healthcare Cost and Utilization Project (HCUP) National Inpatient Sample (NIS) and State Inpatient Databases (SID). The database includes over 22 million individual patient encounters, covers regions where approximately 50% of the US population lives, and includes hospitals responsible for 45% of all admissions. This data set represents the most recent data available via SID and the NIS shared via HCUP where patient residence and hospital ID can be traced back to their geographic location at the HSA or county level. The set consists of all states that chose to share this data (including AZ, AK, CA, CO, FL, MD, MA, NV, NJ, NY, NC, OR, RI, UT, VT, WA, WV, WI). Importantly, the set includes data from all insurers; allowing examination of outcomes on patients with both private and public insurance [50,51]. All patients were included, as well as associated patient-level variables: age, gender, race, length of stay, inpatient mortality, co-morbidities.

### Health outcome measures

**Fig 1:**
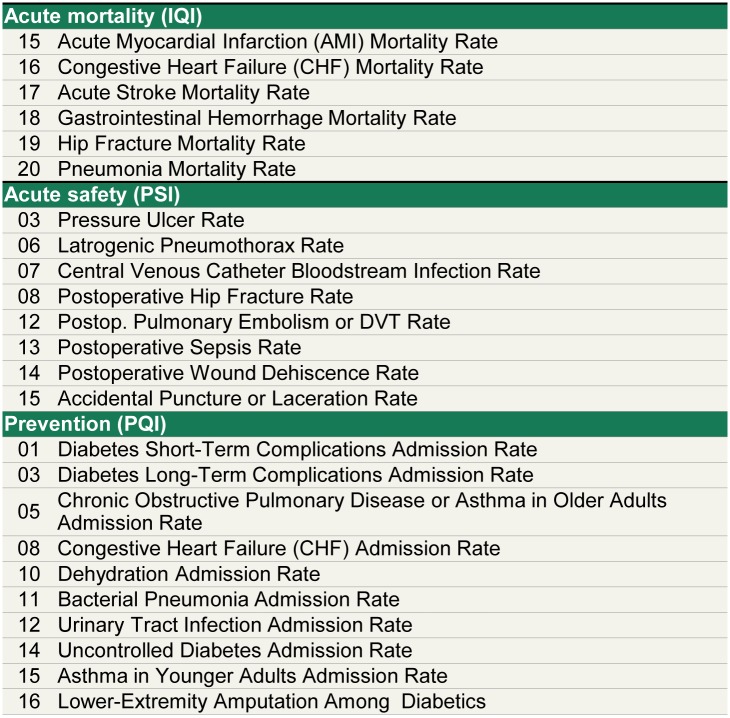
Overview of 24 AHRQ-outcomes investigated. We examined 24 AHRQ-defined outcomes to quantify the degree of geographic variation in outcomes across the US. The outcomes selected are collectively the set of measures which form the combined inpatient mortality (IQI 91), inpatient safety (PSI 90), and prevention (PQI 90) indices respectively, that have currently maintained endorsement by the National Quality Forum through 2015 either individually or as a part of an index.

The outcomes selected for use in this study (Fig 1) are the result of an effort by the Agency for Healthcare Research & Quality (AHRQ) to develop measures of inpatient mortality (IQI), inpatient safety (PSI), and prevention (PQI) for use with inpatient administrative data [52–55]. The IQIs measure the number of in-hospital deaths per number of hospital discharges with a specific principal diagnosis (e.g., principal diagnosis of Acute Myocardial Infarction (AMI)). The PSIs measure the rate of hospital complications (e.g., central venous catheter infection rate) per applicable discharges. The PQIs measure the ratio of the number of hospital admissions for a specific disease (e.g., Congestive Heart Failure) compared to the total number of eligible residents in a given county. See Figure A in S1 File for the definitions of outcomes used.

The 24 AHRQ measures, which have been used broadly by hospitals [56,57] and national and international agencies [58–60], are collectively the set of measures which form the combined mortality (IQI 91), inpatient safety (PSI 90), and prevention (PQI 90) indices respectively [61–64], that have currently maintained endorsement by the National Quality Forum through 2015 either individually or as a part of an index. AHRQ software was used to calculate raw mortality, safety, and inpatient admissions rates using HCUP data following AHRQ technical specifications [65].

### Risk adjustment factors and data sources

**Fig 2:**
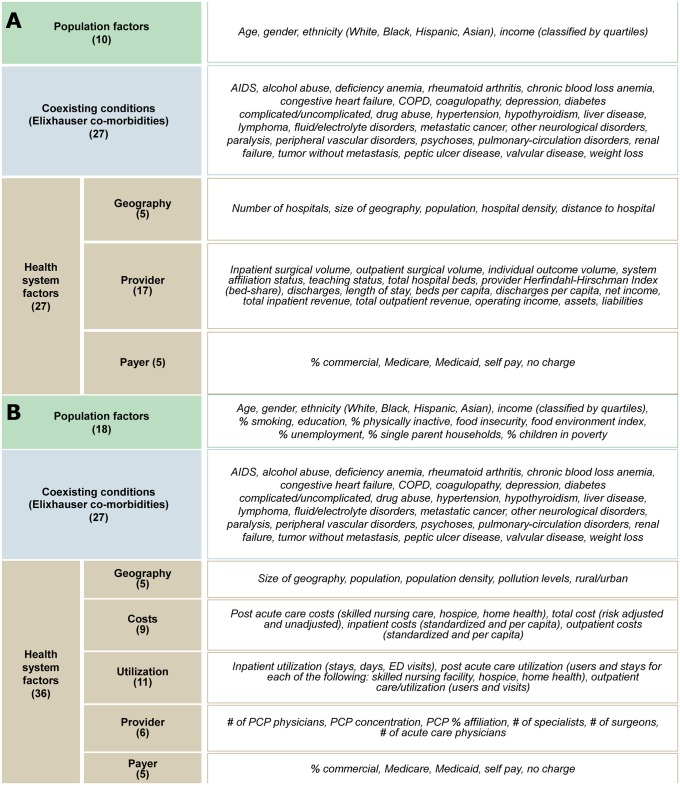
Overview of 64 factors used to risk-adjust inpatient mortality (IQI) and safety (PSI) by hospital and 81 factors used to risk-adjust prevention quality indicators (PQIs) by county. (A) Summary of the 64 factors investigated for IQIs and PSIs including population factors, co-morbidities and health system factors. Each factor was linked to the outcomes at the hospital level. (B) Summary of the 81 factors investigated for PQIs including population factors, co-morbidities and health system factors. Each factor was linked to the outcomes at the county level.

In order to analyze potential factors accounting for variability in outcomes (Fig 2), we assembled two databases containing population, co-morbidities, and health-systems factors. One database was assembled for inpatient mortality (IQIs) and inpatient safety (PSIs) outcomes to measure variation at the hospital level. A second database was assembled for the prevention (PQIs) outcomes to measure variation at the county level. Factors included variables pulled directly from the HCUP database (e.g. demographics, co-morbidities) and factors aggregated from 16 publicly available sources (Figure B in S1 File). Of the sources used, 13 sources are government sources such as the US Census, CMS, and USDA; 3 are academically well-cited and respected private sources [66–68]. From these sources we selected well cited factors identified in the literature as potentially associated with health care outcomes (e.g., demographics, socioeconomics, lifestyle, co-morbidities, utilization, etc.). We then limited the analysis to factors for which we had data for over 95% of hospitals/counties examined. To build the IQI/PSI database, encounter-level HCUP data was aggregated to the hospital level. This hospital level data was then linked to additional sources containing population and system factors for a total of 64 risk-adjustment factors for IQI and PSIs (Figure C in S1 File). Analogously, to build the PQI database, encounter-level HCUP data was aggregated to the county level. This county level data was then linked to additional sources containing population and systems factors for a total of 81 risk-adjustment factors for PQIs (Figure D in S1 File). All told, 10 population factors, 27 co-morbidities and 10 health system factors were consistent across all IQIs, PSIs, and PQIs. An additional 17 health system factors were matched at the hospital level for the IQI/PSI database. An additional 8 population factors and 26 health system factors were matched at the county level for the PQI database.

### Analytic methods

We used AHRQ SAS software to calculate raw un-adjusted outcomes across each of the 24 measures spanning inpatient mortality, inpatient safety, and prevention outcomes. We calculated IQI and PSI outcomes at the hospital level. We then mapped hospital-level outcomes to HSA, HRR, and state regions for further analysis. Those HRRs in which data was only available for less than 50% of their component HSAs were excluded from the HRR level analysis. This was often the case for HRRs that spanned across two states for which HCUP only made available geographically identified data for one of the two states. PQI outcomes were calculated at the county level and geographically mapped to states. The burden of co-morbidities was estimated using the Elixhauser Comorbidity Index [69], which is shown to outperform the Charlson Comorbidity Index [70], by using the Comorbidity Elements included in the HCUP database.

**Fig 3:**
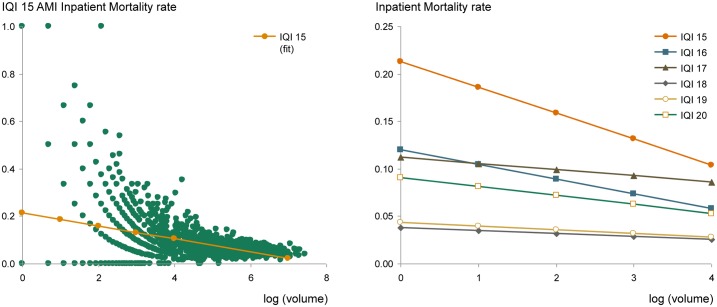
Volume-outcome relationship for inpatient mortality (IQI). We assessed the relationship between outcomes and hospital case volume by modeling the mortality (M) as M = -α*ln(V)+β, where V is hospital case volume, and α and β are constants for each of the inpatient mortality outcomes. As case volume increases in a hospital, mortality decreases across all IQI measures.

We examined the relationship between a hospital's case volume and that hospital's outcomes for the IQIs and PSIs (See Section A in S2 File: Volume-outcome relationship and Fig 3). We used a Bayesian Shrinkage method initiated by Clayton and Kaldor [71] and developed by Dimick and Birkmeyer [72,73]to correct for low-volume hospital noise for all IQI and PSI measures before reporting observed values. The method places more weight on a hospital’s mortality or complication rate when it is measured more reliably, due to a large number of patients, but shrinks back toward the mean complication rate when a hospital’s rate cannot be measured with high reliability due to a low number of events [72–74]. The shrinkage-adjusted IQI and PSI rates were used for subsequent analysis. The volume-outcome relationship was not calculated for PQI events as they are measured as the number of preventable admissions over the total population of a county (i.e., whereas higher procedural volume within a hospital is associated with lower mortality, more populous counties were not expected to show improved preventable admissions rates). Hospitals or counties that were major outliers >5 standard deviations away from the mean were excluded from the analysis (~0.35% of all data points).

To understand the impact of risk-adjustment, we calculated three sets of risk-adjusted outcomes. The first set was risk-adjusted only for population factors, the second for both population factors and co-morbidities and the third for population factors, co-morbidities, and health system factors. After each step we examined the relationship between these factors and each of the outcomes at the hospital level (for IQIs and PSIs) or county level (for PQIs). The analysis was done as a series of unweighted linear regression models. The method was chosen as it (a) allows for an intuitive representation of the R^2^ measure, (b) is the statistical methodology generally used to risk-adjust AHRQ and other similar measures (e.g., normal distribution based risk-adjustment is used by the US Government Center for Medicare & Medicaid Services to adjust mortality and re-admission rates [75]) and (c) is used in a large number of academic publications [19,56,76–81]. Our analysis was conducted step-wise, first, a model with only population variables was used, then a model with population variables and co-morbidities, and finally a full model with population variables, co-morbidities, and system factors. To select key factors from the full set of 64 or 81 factors, dimension reduction was performed using elastic net penalized regression models. The elastic-net method uses both the ridge and lasso penalties, and has the advantages of both approaches. The elastic-net method automatically selects key factors, and thus efficiently resolves the problem caused by multicollinearity [82]. We adjusted the tuning parameter (λ) to select factors such that the model would produce the lowest error. After defining the optimal λ, this value was used to select appropriate population and co-morbidity factors in the model [83],[84]. Lastly, we used a bootstrapping methodology to determine the 95% confidence interval for each model's R-squared value describing the effects of the investigated factors [85]. Bootstrapping uses a resampling with replacement methodology in order to estimate the variance within the sample; in this analysis, the data was resampled 10,000 times.

The AHRQ data fundamentally represent counts. The Poisson distribution is mathematically appropriate for this type of data, and has recently been used in a few publications related to outcomes measures [86–88] Therefore, the analysis was repeated leveraging a Poisson model to confirm results were robust to choice of distribution. Results of this analysis can be found in Section B in S2 File.

Finally, to visualize geographic variation, we created heat maps using ArcGIS and Alteryx. The rate in each HSA was calculated as a weighted average of hospitals in that area. The weighting was done separately for each outcome and was based on the total number of relevant cases—for example, in AMI mortality, the weighting was done based on a total number of AMI patients admitted. Most of the HSAs contain only one hospital, but HCUP data-use restrictions prohibit plotting regions that contain only one hospital. In order to comply with these restrictions, HSAs with a single hospital were merged with the adjacent HSA that had the most closely matched outcome rate and the rate in the new area was calculated based on discharge weighted average of all hospitals in that area.

## Results

**Fig 4:**
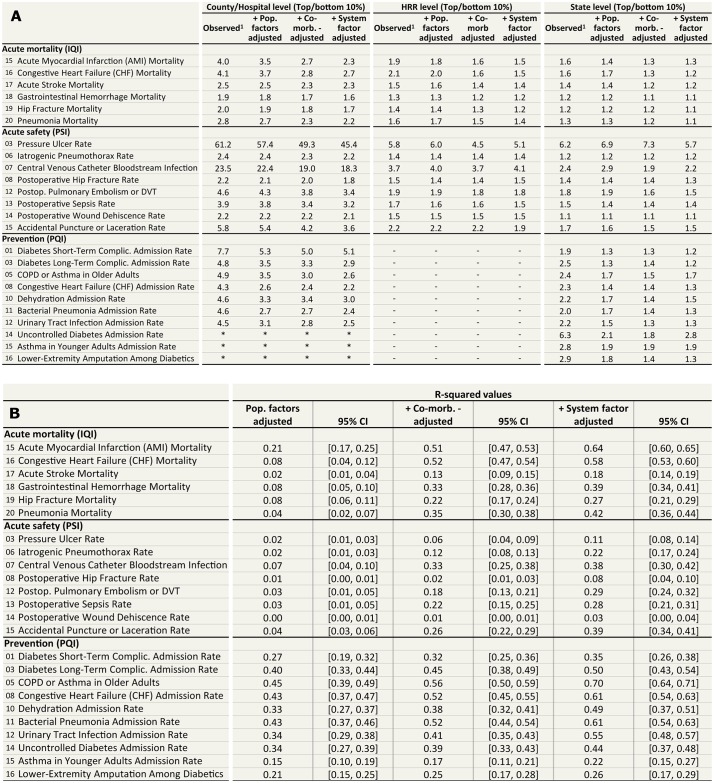
Table quantifying variation in US outcomes. (A) Significant geographic variation exists across all outcomes measures both before and after risk-adjustment. The values in the table quantify the extent of outcomes variation between the top decile and bottom decile geographies. For example, for IQI 15 AMI inpatient mortality, we observe a 4.0-fold difference in outcomes between the top 10% and bottom 10% of hospitals. For IQIs and PSIs, "observed" refers to values that were adjusted for low-volume noise using Bayesian shrinkage method but did not risk adjust for any other factors. For PQIs, the unit of analysis was at the county level, and therefore PQIs did not need to be shrunk. Risk-adjustments are performed incrementally. "+ pop. factors adjusted" values are shrunk and adjusted for populations factors. "+ co-morb. adjusted" values are shrunk, adjusted for population factors, and adjusted for co-morbidities. Lastly, "+ system adjusted" values are shrunk, population adjusted, co-morbidities adjusted, and system factors adjusted. For IQI 15 AMI inpatient mortality, we observe a 2.3-fold difference in outcomes between the top 10% and bottom 10% of hospitals after risk-adjustment for demographic, co-morbidities, and health system factors. Dash (-) indicates numbers that were not calculated. Counties were not mapped to HSA or HRR, and therefore PQI ratios were not determined. Star (*) indicates a D1 (top decile) value of 0, such that it was not possible to calculate a ratio. (B) R-squared values with 95% confidence intervals are shown. For the confidence intervals [X, Y], X refers to the lower bound of a given R-squared value; Y refers to the upper bound of a given R-squared value. For example, for IQI 15 AMI inpatient mortality, we are able to account for 64% of the variability in outcomes after risk-adjusting for demographics, co-morbidities, and health system factors.

**Fig 5:**
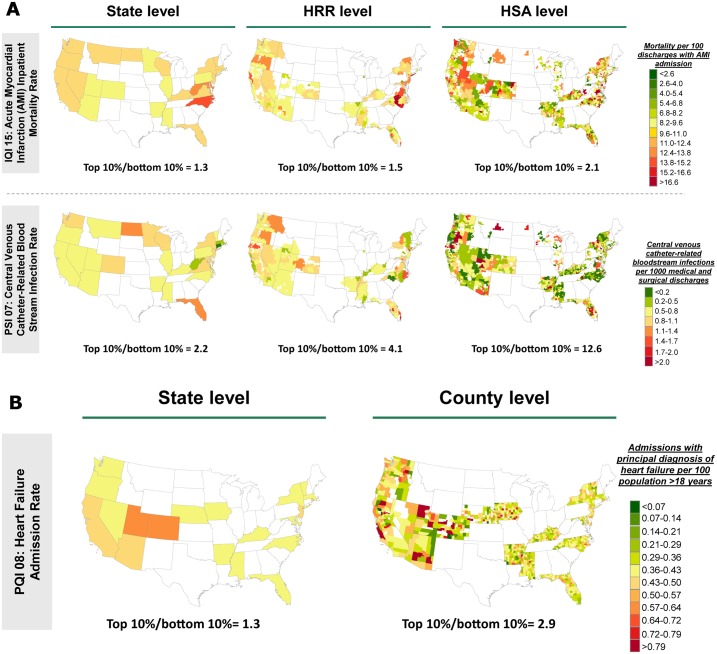
Fully risk-adjusted geographic distributions of select outcomes (IQI 15, PSI 07, PQI 08). Large variation in outcomes is present both between and within US states. Substantially different performances highlight the variation in outcomes across the US. This variation is observed across all outcomes plotted. (A) IQI 15 Acute Myocardial Infarction (AMI) Mortality Rate and PSI 07 Central Venous Catheter-Related Blood Stream Infection Rate are adjusted for low-volume noise using a Bayesian shrinkage methodology and are adjusted for population, co-morbidities, and health system factors. After risk-adjustment, there is 2.1-fold variation in IQI 15 between the top and bottom decile HSAs. After risk-adjustment, there is 12.6-fold variation in PSI 07 between the top and bottom decile HSAs. (B) PQI 08 Heart Failure Admission Rate data has been adjusted for population, co-morbidities, and health system factors. After risk-adjustment, there is 2.2-fold variation in PQI 08 between the top and bottom decile counties. Areas shown in white are due to HCUP not making geographically identifiable data on hospital or county performance available.

Large variation in health outcomes exists at the hospital level for IQIs and PSIs; and at the county level for PQIs (Fig 4 and Figure E in S1 File). Variability decreases as outcomes are risk-adjusted for population, co-morbidities, and health system factors (Fig 4). As expected, variability also decreases as outcomes are aggregated into larger geographic units from the hospital to HSA to HRR to State level (Fig 5).

For inpatient mortality measures, combined population, co-morbidities, and health systems factors account for a range of R^2^ between 18% and 64% of variability depending on the outcome, with an average of 41%. For patient safety measures, combined population, co-morbidities, and health systems factors account for a range of 3% to 39% of safety measure variability, with an average of 22%. Finally, for prevention measures, combined population, co-morbidities, and health systems factors account for a range of 22% to 70% of prevention measure variability, with an average of 47%. For example, consider acute myocardial infarction: population factors accounted for 21% of variation, adding co-morbidities accounted for an incremental 30% of variation, and adding system factors accounted for an incremental 13% of variation.

The specific variability of IQIs, PSIs, and PQIs at the hospital, county, HSA, HRR, and state level are outlined below. Results are listed both before and after risk-adjustment.

### IQIs

At the hospital level, IQIs have 90th to 10th percentile observed ratios (top 10%/bottom 10%) of between 1.9 and 4.1, with an average of 2.9. At the HSA level, IQIs have observed ratios (top 10%/bottom 10%) of between 1.7 and 3.6, with an average of 2.5. At the HRR level, IQIs have observed ratios (top 10%/bottom 10%) of between 1.3 and 2.1, with an average of 1.6. And lastly, at the state level, IQIs have observed ratios (top 10%/bottom 10%) of between 1.2 and 1.6, with an average of 1.4. Variation in health outcomes decreases as data is aggregated to larger geographies. Among IQIs, Gastrointestinal Hemorrhage Mortality Rate had the lowest amount of variation, and Congestive Heart Failure Mortality rate had the highest amount of variation.

Variation decreases after risk-adjustment. At the hospital level, IQIs have adjusted ratios (top 10%/bottom 10%) of between 1.6 and 2.6, with an average of 2.1. At the HSA level, they have adjusted ratios between 1.5 and 2.4, with an average of 2.0 and at the HRR level, of between 1.2 and 1.5, with an average of 1.4. Finally, at the state level, IQIs have adjusted ratios (top 10%/bottom 10%) of between 1.1 and 1.3, with an average of 1.2.

Consider IQI 15: Acute Myocardial Infarction Inpatient Mortality Rate. Before risk-adjustment, we observed a 4.0 fold variation in AMI inpatient death rates between the top decile and bottom decile of hospitals. After risk-adjustment for population, co-morbidity and health system factors, we observed a 2.3 fold variation in AMI inpatient death rate between the top decile and bottom decile of hospitals.

### PSIs

At the hospital level, PSIs have observed ratios (top 10%/bottom 10%) of between 2.2 and 61.3, with an average of 13.2. At the HSA level, they have observed ratios (top 10%/bottom 10%) of between 1.8 and 32.6, with an average of 8.2 and at the HRR level, between 1.4 and 5.8, with an average of 2.5. And lastly, at the state level, PSIs have observed ratios (top 10%/bottom 10%) of between 1.1 and 6.2, with an average of 2.2. Variation in health outcomes decreases as data is aggregated to larger geographies. Among PSIs, Postoperative Hip Fracture rate had the lowest amount of variation, and Pressure Ulcer Rate had the highest amount of variation.

Similarly to IQIs, variation decreases after risk-adjustment. At the hospital level, PSIs have adjusted ratios (top 10%/bottom10%) of between 1.8 and 46.9, with an average of 10.2. At the HSA level, PSIs have adjusted ratios (top 10%/bottom 10%) of between 1.6 and 29.8, with an average of 7.1. At the HRR level, PSIs have adjusted ratios (top 10%/bottom 10%) of between 1.4 and 5.1, with an average of 2.3. At the state level, PSIs have adjusted ratios (top 10%/bottom 10%) of between 1.1 and 5.7, with an average of 2.0.

Consider PSI 07: Central Venous Catheter Bloodstream Infection. Before risk-adjustment, we observed a 23.9 fold variation in CVC infection rates between the top decile and bottom decile of hospitals. After risk-adjustment for population, co-morbidity and health system factors, we observed an 18.7 fold variation in CVC infection rates between the top decile and bottom decile of hospitals.

### PQIs

At the county level, PQIs have observed ratios (top 10%/bottom 10%) of between4.3 and 7.7, with an average of 5.1. At the state level, PQIs have observed ratios (top 10%/bottom 10%) of between 1.9 and 6.3, with an average of 2.8. Variation in health outcomes decreases as data is aggregated to larger geographies. Among PQIs, Bacterial Pneumonia Admission Rate had the lowest amount of variation, and Uncontrolled Diabetes Admission Rate had the highest amount of variation.

Again, variation decreases after risk-adjustment. At the county level, PQIs have adjusted ratios (top 10%/bottom 10%) of between 2.2 and 5.1, with an average of 3.0. At the state level, PQIs have adjusted ratios (top 10%/bottom 10%) of between 1.2 and 2.8, with an average of 1.6. Counties do not naturally map to HSA and HRR. Therefore, PQIs were not aggregated to HSA and HRR; PQIs were analyzed at the county and state level only.

Consider PQI 08: Congestive Heart Failure (CHF) Admission Rate. Before risk-adjustment, we observed a 4.3 fold variation in CHF admission rates between the top decile and bottom decile of counties. After risk-adjustment for population, co-morbidity and health system factors, we observed a 2.2 fold variation in CHF admission rates between the top decile and bottom decile of counties.

### Correlations

**Fig 6:**
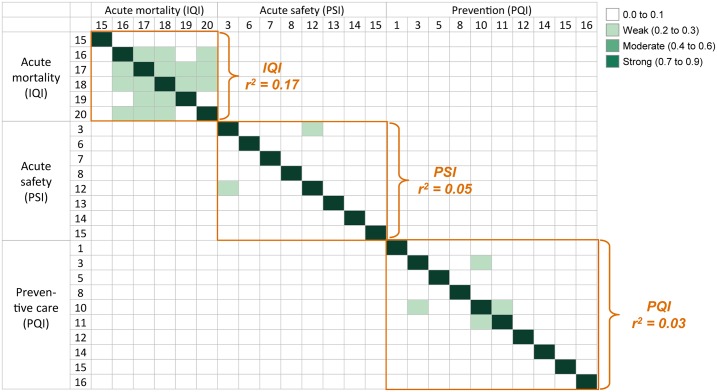
Correlations among outcomes after adjustment for population factors and co-morbidities and system factors. Inpatient mortality measures are weakly correlated with each other. Inpatient safety measures show little to no correlation with each other. Prevention quality measures show little to no correlation with each other. Correlation categorization after Dancey and Reidy (2004), analysis following low denominator number outlier removal and risk-adjustment based on the identified population factors, co-morbidities and system factors.

We examined the correlation between risk-adjusted outcomes (Fig 6). To understand the degree of correlation between outcomes, we analyzed cross correlations between each outcome pair after full risk correction (including population factors, co-morbidities and system factors). There exists little or no correlation between IQIs and PSIs at the hospital level. IQIs have an average correlation coefficient of 0.17. PSIs have an average correlation coefficient 0.05, and PQIs have an average correlation coefficient of 0.03. Additionally, there exists effectively no correlation between IQIs, PSIs, and PQIs at the county level.

## Discussion

Extensive prior research has provided insight into geographic variation [14] in healthcare cost. However, geographic variation in outcomes had not been quantified at a similar level of rigor or granularity.[20,89] The timeliness of this question has been underscored by CMS recently publishing an online tool that maps Medicare-only disparities in cost, prevalence and select outcome measures such as readmission rates [90]. In our study, which covers the all-payer population, we demonstrate that the magnitude of HSA and county level variability in US health care outcomes is large and exceeds the variability observed in US health care costs. Specifically, a 1.7 to 32.6-fold difference between HSAs and counties in the 90th percentile and those in the 10th percentile across 24 non-risk-adjusted AHRQ outcomes was observed. Each outcome examined has larger variability than the 1.7-fold variability in health care costs observed between the top and bottom decile HSAs [14].

As expected, variability decreases as outcomes are aggregated over larger geographies. These results are consistent with prior research suggesting that analysis averaging across large geographies masks the true extent of variation^51^. In short, a tremendous amount of variability exists *within* the 27 states and *within* the 201 HRRs, which is only identified by examining the 1,295 HSAs. Studies conducted at the state level or HRR level are inadequate for characterizing the extent of geographic variation in the quality of US health care delivery.

The population, co-morbidity, and health system factors accounted for a range of R^2^ between 18–64% of variability in mortality outcomes, 3–39% of variability in patient safety outcomes, and 22–70% of variability in prevention outcomes. Significant amounts of variability in outcomes can be accounted for by the population, co-morbidity, and health system factors, but, as expected, meaningful variation in outcomes remains even after completing risk-adjustment with 61 to 84 factors. The demographic and co-morbidity factors used are standardized and well developed. The health system factors used are externally observable and publically available factors such as hospital size, length of stay, and case volume.

Through the comprehensive risk adjustment of this study, we find select US hospitals serving complex and disadvantaged patient populations that deliver outstanding risk-adjusted outcomes. Conversely, we find select US hospitals serving relatively healthy and wealthy patients that deliver lagging risk-adjusted outcomes. Still, the study demonstrates a significant residual outcomes variation after risk-adjustment.

The existence of meaningful residual outcomes variation after risk-adjustment with publically available demographic, co-morbidity, and health system factors implies that other factors impact outcomes. Said differently, there is meaningful outcomes variation when different hospitals treat what appear to be similar acute myocardial infarction patients, or indeed when they treat patients with any of the measured diagnoses. We hypothesize that the residual unexplained variability is likely to be driven by factors *inside* the hospitals (e.g., department specific care protocols, culture, and experience of the clinical teams) which are not publically observable. For example, recently published in-depth site visits and interviews of US hospitals in the top and bottom 5% of risk-standardized AMI mortality found that performance results are centered on a supportive organizational culture that encourages engagement in quality, strong communication and coordination among groups; and the capacity for problem solving and learning in the organization.[91] Culture, communication, and similar factors are not publically reported, but they likely account for a portion of the residual variation in our study.

Publicly available risk-adjusted outcomes data would enable patients, physicians, and policymakers to challenge past assumptions. Without outcomes data transparency, patients cannot make informed decisions, hospitals may not know where to focus quality improvement initiatives, and policy makers are stuck measuring adherence to process measures. Quantifying the geographic variation in risk-adjusted US health outcomes is the first step toward improving outcomes for patients and enabling meaningful improvements in health care productivity.

This paper has several limitations. First, the analysis is limited to factors for which publically reported data is currently available. Additional factors for which publically reported data is not available are not accounted for in this analysis [92]. Potential factors accounting for outcomes variation which are not captured in this study include: degree of health system integration, physician skill, hospital care protocols, differences in clinical practice, culture, communication, approach to behavioral health in provider treatment routines, and many others. Further research that incorporates data from "within" the hospital is required in order to quantify remaining drivers of residual outcome variability. Identifying the drivers of the variability, and also understanding root causes of outcome variation, is critical to minimizing outcome variability and improving US health outcomes.

Second, while we used 16 robust data sources, none of the administrative datasets have the clinical richness found in electronic medical records. Furthermore, the administrative data we used is limited to the inpatient setting, such that we were not able to assess care delivered in ambulatory settings, skilled nursing facilities, or other sites of care. Additionally, while HCUP SID data provided the ability to examine an all-payer population, it is limited by inability to track patients longitudinally. As such we are unable to identify if a patient had more than one admission in a given year (although direct hospital-to-hospital transfers are excluded from this study). The administrative data limitation affects the accuracy of the acute mortality, acute safety, and prevention outcomes measures in different ways. For PSI inpatient safety, the administrative nature of the dataset is particularly limiting, as it relies on subjective reporting (and coding) of these complications. PSI data may suffer from reporting bias, as has been suggested to exist for pressure ulcers.[93,94] For PQI prevention measures, administrative data will have higher accuracy given that inpatient admissions rates are objectively observable. However, one can only measure failures of prevention when a patient appears in the hospital. We are unable to assess failures of prevention where a sick patient is never admitted to the hospital, such as a Congestive Heart Failure patient who dies at home. For IQI inpatient mortality, we are highly confident in the accuracy of the outcomes, as death in the hospital is observable and reliable. Still, in this study, a patient who is discharged to hospice or to die at home following an acute hospitalization would be indistinguishable from one who returned home in full health. Our confidence in the IQI outcomes data is further strengthened by observing a significant inverse logarithmic relationship between inpatient mortality outcomes and hospital case volume, similar to what has been previously observed in the literature [95–98]. (See Section A in S2 File: Volume-outcome relationship and Fig 3). As case volume increases in a given hospital, the IQI mortality rate decreases. For example, consider acute myocardial infarction: for hospitals with 50 or fewer AMI cases, the average inpatient mortality rate is 13%, while for hospitals with more than 200 AMI cases it is 5% (p<0.001).

Third, our study is limited by the fundamental lack of geographic outcomes data transparency in the US. We examined the complete set of 2011 publically available SID and NIS data through HCUP, but the available data covers only 50% of the United States population and was limited to a single year. In 2011, not all states participated in SID, and only a subset of states chose to disclose patient or hospital information down to the county level. To investigate how representative data from a single year is, a supplemental longitudinal analysis of outcomes using SID data from the State of New York over the 11-year period from 2002 to 2012 was performed (See Section C in S2 File: Longitudinal Analysis and Figure G in S1 File). This analysis demonstrated that hospital performance showed similar large levels of variation each year during the 11-year period. When the State of New York data was aggregated over the entire period, each individual hospital showed meaningful persistence in performance from year to year. Therefore, despite these limitations, we believe that the data assembled is effective and has led to several important conclusions.

Fourth, the study is limited by the reductions in HCUP data transparency which occurred after 2011. Since 2011, changes in the design of the Healthcare Cost and Utilization Project (HCUP) database dramatically *decreased* the number of records where geographic identification was possible, and the number of State Inpatient Databases (SID) that include geographic data has been reduced significantly [99],[100]. Without access to comprehensive longitudinal data sets, further research investigating the temporal trends of outcomes variation is impossible. While we found persistence in performance through a supplemental longitudinal analysis (text C in S2 File), additional research using HCUP data in years past 2011 is not feasible due to changes in the design of the database after 2011. HCUP/NIS/SID should increase geographic outcomes data transparency by reverting back to the 2011 disclosure level. Recent changes which dramatically reduced the number of records where geographic identification was possible are a step in the wrong direction.

Despite these limitations, this study sheds light on the magnitude of health care outcomes variation across the United States and highlights the importance of increased outcomes data transparency and further research on outcomes variation.

## Conclusion

The amount of variability in health outcomes in the United States is large and exceeds that of cost variability. This variability persists even after adjusting for differences in population, co-morbidities, and health system factors. Population factors, co-morbidities, and health system factors play a meaningful role in accounting for a portion of this variation; however, a large amount of variation remains unaccounted for. The geographic variability in healthcare outcomes has implications for all health care stakeholders—patients, physicians, hospitals, payers, policymakers, pharmaceutical companies, and medical technology companies. These findings suggest that: 1) additional examination of regional and local variation in risk-adjusted outcomes should be a priority; 2) assumptions of uniform hospital quality that underpin rationale for policy choices (such as narrow insurance networks or antitrust enforcement) should be challenged; and 3) there exists substantial opportunity for outcomes improvement in the US healthcare system.

## Supporting Information

S1 File **Figure A**: Summary and definitions of the 24 AHRQ outcomes measures investigated. Each IQI represents the number of hospital deaths per 1,000 hospital discharges with a specific condition (e.g., Acute Myocardial Infarction (AMI)) as principal diagnosis for patients. PSIs describe the rate of surgical complications (e.g., wound dehiscence) following applicable interventions. PQIs provide a ratio of the number of hospital admissions for a specific disease (e.g., Congestive Heart Failure) to the total number of eligible residents in a given county. **Figure B: Overview of data sources used.** Summary of the 16 data sources used to assemble a database of 64 population, co-morbidities, and systems factors for IQIs and PSIs, and a database of 81 population, co-morbidities, and systems factors for PQIs. 13 are government sources; 3 are highly respected private sources^65–67^. The year of the data and the data assembled from each data source is listed. All sources contain data for >95% of hospitals/counties investigated. **Figure C: Overview of 64 potential factors investigated for IQIs and PSIs.** Summary and definitions of 64 potential factors investigated for IQIs and PSIs. We assembled a database from 6 sources of potential factors, including population factors such as demographics, lifestyle, and socioeconomics, as well as co-morbidities and health system factors (such as physician supply and hospital bed supply). Each factor was linked at the hospital level. **Figure D: Overview of 81 potential factors investigated for PQIs.** Summary and definitions of 81 potential factors investigated for PQIs. We assembled a database from 14 sources of potential factors, including population factors such as demographics, lifestyle, and socioeconomics, as well as co-morbidities and health system factors (such as physician supply and hospital bed supply). Each factor was linked at the county level. **Figure E: Comparison of outcomes variability, measured via D9/D1 ratio between risk adjustments conducted with a Gaussian distribution and Poisson distribution.** Fields marked in red indicate that the two results are meaningfully (>25%) different. **Figure F: Maps of geographic variation in the United States.** These maps show geographic variability in each of the 24 outcomes studied. All values on the map are adjusted for low-volume noise using empirical Bayesian shrinkage method, however they are not risk adjusted for population factors, co-morbidities and health system factors to enable the reader to see the variation before the risk adjustment. Additionally, all HSAs with only one hospital were merged with adjacent HSA so that the resulting region contains two hospitals as required by HCUP's data use agreement. **Figure G: Persistence of hospital/county performance over 11-years.** All outcomes measures show a high degree of persistence. Inpatient mortality has 69% persistence, inpatient safety has 67% persistence, and prevention has 85% persistence. To calculate, inpatient mortality and inpatient safety measures were first shrunk using Bayesian shrinkage. Then the variation each year was assessed by calculating Top 10%/bottom 10% ratio. Persistence in hospital performance was evaluated by ranking each hospital every year into deciles, as well as ranking each hospital based on its 11-year cumulative performance. Percent of time (years) in which a hospital was within two deciles of its 11-year rank was defined as persistence.
(PDF)

S2 File Summary of additional analyses. (PDF)
