# Supplementary material for: Quantifying Geographic Variation in Health Care Outcomes in the United States before and after Risk-Adjustment
Source: PLoS One. 2016 Dec 14;11(12):e0166762. doi: 10.1371/journal.pone.0166762 (PMC5156342; doi:10.1371/journal.pone.0166762)
Supplement: S1 File — Figure A: Summary and definitions of the 24 AHRQ outcomes measures investigated. Each IQI represents the number of hospital deaths per 1,000 hospital discharges with a specific condition (e.g., Acute Myocardial Infarction (AMI)) as principal diagnosis for patients. PSIs describe the rate of surgical complications (e.g., wound dehiscence) following applicable interventions. PQIs provide a ratio of the number of hospital admissions for a specific disease (e.g., Congestive Heart Failure) to the total number of eligible residents in a given county. Figure B: Overview of data sources used. Summary of the 16 data sources used to assemble a database of 64 population, co-morbidities, and systems factors for IQIs and PSIs, and a database of 81 population, co-morbidities, and systems factors for PQIs. 13 are government sources; 3 are highly respected private sources65–67. The year of the data and the data assembled from each data source is listed. All sources contain data for >95% of hospitals/counties investigated. Figure C: Overview of 64 potential factors investigated for IQIs and PSIs. Summary and definitions of 64 potential factors investigated for IQIs and PSIs. We assembled a database from 6 sources of potential factors, including population factors such as demographics, lifestyle, and socioeconomics, as well as co-morbidities and health system factors (such as physician supply and hospital bed supply). Each factor was linked at the hospital level. Figure D: Overview of 81 potential factors investigated for PQIs. Summary and definitions of 81 potential factors investigated for PQIs. We assembled a database from 14 sources of potential factors, including population factors such as demographics, lifestyle, and socioeconomics, as well as co-morbidities and health system factors (such as physician supply and hospital bed supply). Each factor was linked at the county level. Figure E: Comparison of outcomes variability, measured via D9/D1 ratio between risk adjustments condu [file pone.0166762.s001.pdf]

Quantifying geographic variation in health care outcomes in the United States before and after risk-adjustment

Supplementary Figures

B Rosenberg et al.

November 8, 2016

Figure A: Overview of outcomes investigated

**Acute mortality (IQI)**	
15 AMI Mortality	In-hospital deaths per number of hospital discharges with AMI as a principal diagnosis for patients ages >18 years
16 Congestive Heart Failure (CHF) Mortality	In-hospital deaths per number of hospital discharges with CHF as a principal diagnosis for patients ages >18 years
17 Acute Stroke Mortality Rate	In-hospital deaths per number of hospital discharges with acute stroke as a principal diagnosis for patients ages >18 years
18 Gastrointestinal Hemorrhage Mortality	In-hospital deaths per number of hospital discharges with GI hemorrhage as a principal diagnosis for patients age >18 yrs
19 Hip Fracture Mortality Rate	In-hospital deaths per number of hospital discharges with hip fracture as a principal diagnosis for patients ages >65 years
20 Pneumonia Mortality Rate	In-hospital deaths per number of hospital discharges with pneumonia as a principal diagnosis for patients ages >18 years
**Acute safety (PSI)**	
03 Pressure Ulcer	Stage III or IV pressure ulcers (secondary diagnosis) among patients ages >18 years
06 Latrogenic Pneumothorax	Iatrogenic pneumothorax cases (secondary diagnosis) among surgical & medical discharges for patients ages >18 years
07 Central Venous Catheter Bloodstream Infection	Central venous catheter-related bloodstream infections (secondary diagnosis) among medical and surgical discharges for patients ages >18 years or obstetric cases.
08 Postoperative Hip Fracture	Postoperative hip fracture (secondary diagnosis) per 1,000 surgical discharges for patients ages >18 years
12 Periop. Pulmonary Embolism or DVT	Periop pulmonary embolism or deep vein thrombosis (secondary diagnosis) per 1,000 surgical discharges for patients >18 yr
13 Postoperative Sepsis Rate	Postoperative sepsis cases (secondary diagnosis) per 1,000 elective surgical discharges for patients ages >18 years
14 Postoperative Wound Dehiscence Rate	Postoperative re-closures of the abdominal wall per 1,000 abdominopelvic surgery discharges for patients ages >18 years
15 Accidental Puncture or Laceration Rate	Accidental punctures or lacerations (secondary diagnosis) during procedure per 1,000 discharges for patients ages >18 yrs
**Prevention (PQI)**	
01 Diabetes Short-Term Complic. Admissions	Admissions with principal diagnosis of diabetes with short-term complications (ketoacidosis, hyperosmolarity, coma) >18 yr
03 Diabetes Long-Term Complic. Admissions	Admissions with principal diagnosis of diabetes with long-term complications (renal, eye, neurological, circulatory, or complications not otherwise specified), ages >18 years
05 COPD or Asthma in Older Adults	Admissions with principal diagnosis of COPD or asthma, ages >40 years
08 Congestive Heart Failure (CHF) Admission	Admissions with principal diagnosis of heart failure, ages >18 years
10 Dehydration Admission Rate	Admissions with principal diagnosis of dehydration, ages >18 years
11 Bacterial Pneumonia Admission Rate	Admissions with principal diagnosis of bacterial pneumonia, ages >18 years
12 Urinary Tract Infection Admission Rate	Admissions with principal diagnosis of urinary tract infection, ages >18 years
14 Uncontrolled Diabetes Admission Rate	Admissions with principal diagnosis of diabetes without mention of short or long-term complications, > 18yrs
15 Asthma in Younger Adults Admission Rate	Admissions with principal diagnosis of asthma, ages 18 to 39 years.
16 Lower-Extremity Amputation Among Diabetics	Admissions with any-listed diagnosis of diabetes and procedure of lower-extremity amputation, ages >18 years

Copyright © 2014 by The Boston Consulting Group, Inc. All rights reserved.

Draft—for discussion only

Figure A : Footnote and methods

Overview of outcomes investigated

Summary and definitions of the 24 AHRQ outcomes measures investigated. Each IQIs represent the number of hospital deaths per 1,000 hospital discharges with a specific condition (e.g., Acute Myocardial Infarction (AMI)) as principal diagnosis for patients. PSIs describe the rate of surgical complications (e.g., wound dehiscence) following applicable interventions. PQIs provide a ratio of the number of hospital admissions for a specific disease (e.g., Congestive Heart Failure) to the total number of eligible residents in a given county.

Copyright © 2014 by The Boston Consulting Group, Inc. All rights reserved.

Draft—for discussion only

Figure B: Overview of data sources used

Source	Government Affiliation	Data used	Year
HCUP/NIS	Yes	Age, gender, ethnicity, income, co-morbidities, payer type, outcome volume, length of stay	2011
US Census	Yes	Population, income, education levels	2010
CDC/BRFSS	Yes	% smoking, % physically inactive	2006-2012
Bureau of Labor Statistics	Yes	% unemployment	2012
American Community Survey, 5-year estimates	Yes	% children in single parent households	2008-2012
Small Area Income and Poverty Estimates	Yes	% children in poverty	2012
USDA Economic Research Services	Yes	Rural/urban	2013
CMS	Yes	Costs, utilization	2012
Hospital Compare (CMS)	Yes	# of hospitals, distance to hospital	2011
AHA Survey	Yes	# hospital beds, provider HHI, # discharges	2012
USDA Food Environment Atlas	Yes	Food environment index	2010-2011
Physician Compare (CMS)	Yes	# of providers, provider affiliation status	2013-2014
Safe Drinking Water Information System (EPA)	No	Water violations	2012-2013
Dartmouth Atlas	No	# of hospitals in geography, distance to hospital	2011
Map the Meal Gap	No	Food insecurity	2011
American Hospital Directory	No	Revenue, volume, hospital affiliation status, hospital teaching status	2012

Copyright © 2014 by The Boston Consulting Group, Inc. All rights reserved.

Figure B : Footnote and methods

Overview of data sources used

Summary of the 16 data sources used to assemble a database of 64 population, co-morbidities, and systems factors for IQIs and PSIs, and a database of 81 population, co-morbidities, and systems factors for PQIs. 13 are government sources; 3 are highly respected private sources[65–67]. The year of the data and the data assembled from each data source is listed. All sources contain data for >95% of hospitals/counties investigated.

Copyright © 2014 by The Boston Consulting Group, Inc. All rights reserved.

Figure C: Overview of 64 potential drivers investigated for IQIs and PSIs (I of II)

**Population factors**	
Age	Age in years at admission 0-124yr. Calculated from the birth date (DOB) and the admission date (ADATE). AGE is set to the supplied age if the age cannot be calculated (ADATE and/or DOB missing or invalid).
Gender	Gender (0) male, (1) female. Provided by the data source. All non-male, non-female (e.g., "other") values are set to missing. If FEMALE is inconsistent with diagnoses (EDX03) or procedures (EPR03), FEMALE is set to inconsistent
Ethnicity (4)	(1) White, (2) black, (3) Hispanic, (4) Asian or Pacific Islander
Income (4)	Quartile classification of the estimated median household income of residents in the patient's ZIP Code<br>1: $1 - $38,999<br>2: $39,000 - $47,999<br>3: $48,000 - $63,999<br>4: 64,000+
**Co-morbidities**	
AIDS	Co-morbidity measures are assigned using the AHRQ co-morbidity software. The AHRQ co-morbidity measures identify coexisting medical conditions that are not directly related to the principal diagnosis, or the main reason for admission, and are likely to have originated prior to the hospital stay. Co-morbidities are identified using ICD-9-CM diagnoses and the Diagnosis Related Group (DRG) in effect on the discharge date. The prefix "CM_" has been added to the AHRQ co-morbidity software data element names to distinguish the co-morbidity measures from other HCUP data elements. For more information, please refer to the materials available on the Tools and Software page of the HCUP User Support Website (http://www.hcup-us.ahrq.gov/tools_software.jsp).
Alcohol	
Rheumatoid arthritis/collagen vascular diseases	
Chronic blood loss anemia	
Congestive Heart Failure	
Chronic pulmonary disease	
Coagulopathy	
Depression	
Diabetes, uncomplicated	
Diabetes with chronic complications	
Drug abuse	
Hypertension (complicated/uncomplicated)	
Hypothyroidism	
Liver disease	
Lymphoma	
Fluid and electrolyte disorders	
Metastatic cancer	
Other neurological disorders	
Paralysis	
Peripheral vascular disorders	
Psychoses	
Pulmonary-circulation disorders	
Renal failure	

Copyright © 2014 by The Boston Consulting Group, Inc. All rights reserved.

Figure C: Overview of 64 potential drivers investigated for IQIs and PSIs (II of II)

**Co-morbidities (cont'd)**	
Solid tumor without metastasis	
Peptic ulcer disease (excluding bleeding)	
Valvular disease	
Weight loss	
**Health system factors**	
Number of hospitals in geography	Count of hospitals that fall in particular geography
Size of geography	Square miles
Population of geography	Use ZIP to geography conversion to account for all ZIPs encompassed within geography
Population density	Population of geography / size of geography
Average distance to hospital	Calculate a "flight" straight line distance from each population block to the geographical center of geography and calculates weighted average of all distances based on the population in this block. Divide this by the number of hospitals in this HSA
Number of procedures/volume	Number of procedures specific to each IQI/PSI calculated by looking at the denominator for each measure
IP surgical volume	Estimated IP surgeries
OP surgical volume	Estimated OP surgeries
Affiliation status	Binary if affiliated with system
Teaching status	Binary if teaching hosp
Total hospital beds	Number of beds regularly maintained (set up and staffed for use) for inpatients as of the close of the reporting period. Excludes newborn bassinets
Provider HHI (bed-share)	Herfindahl–Hirschman Index based on hospital total beds (i.e. share of beds in the HSA) Calculate HHI for all hospitals in this HSA but use number of beds rather than market share
Discharges	Total number of hospital discharges
LOS	Length of stay (LOS) is calculated by subtracting the admission date (ADATE) from the discharge date (DDATE). Same-day stays are therefore coded as 0. Leave days are not subtracted.
Beds per capita	Number of beds divided by population in each HSA
Discharges per capita	Number of discharges divided by population in each HSA
Hospital net income	Net income (L968)<br>Net income (or loss) is taken from a hospital's most recent Medicare Cost Report (W/S G-3, line 29, column 1)
Hospital revenue (2)	• Total Inpatient Revenue<br>• Total Outpatient Revenue
Hospital operating income	Operating income
Hospital assets/liabilities (2)	• Total assets<br>• Total liabilities
Payer type (5)	Percent of records at hospital with each of the primary payers: Medicare, Medicaid, private, self pay, no charge

Copyright © 2014 by The Boston Consulting Group, Inc. All rights reserved.

Figure C: Footnote and methods

Overview of 64 potential drivers investigated for IQIs and PSIs

Summary and definitions of 64 potential drivers investigated for IQIs and PSIs. We assembled a database from 6 reputable sources of potential drivers, including population factors such as demographics, lifestyle, and socioeconomics, as well as co-morbidities and health system factors (such as physician supply and hospital bed supply). Each factor was linked at the hospital level.

Copyright © 2014 by The Boston Consulting Group, Inc. All rights reserved.

Figure D: Overview of 81 potential drivers investigated for PQIs (I of III)

**Population factors**	
Age	Age in years at admission 0-124yr. Calculated from the birth date (DOB) and the admission date (ADATE). AGE is set to the supplied age if the age cannot be calculated (ADATE and/or DOB missing or invalid).
Gender	Gender (0) male, (1) female. Provided by the data source. All non-male, non-female (e.g., "other") values are set to missing. If FEMALE is inconsistent with diagnoses (EDX03) or procedures (EPR03), FEMALE is set to inconsistent
Ethnicity (4)	(1) White, (2) black, (3) Hispanic, (4) Asian or Pacific Islander
Smoking	Percent of adults that report smoking at least 100 cigarettes in their lifetime and that they currently smoke
Income (4)	Quartile classification of the estimated median household income by county
Education	Percent of adults (>25yr) with:<br>• Some college <1yr
Physical activity	Percent of adults (>20yr) reporting no leisure-time physical activity - defined as a "no" response to survey question, "During the past month, other than your regular job, did you participate in any physical activities or exercise, such as running, calisthenics, golf, gardening, or walking for exercise?"
Food quality (2)	Food insecurity is modeled by analyzing the relationship between food insecurity and indicators of food insecurity (poverty, unemployment, median income, etc.) at the state level. Then use the coefficient estimates from this analysis plus information on the same variables defined at the county level to generate estimated food insecurity rates at county level.
	Food Environment Index<br>Index of factors that contribute to a healthy food environment developed by County Health. This index ranges from 0 (worst) to 10 (best) which equally weights limited access to healthy food and food insecurity.
Employment	Percent of population (>16yr) unemployed but seeking work
Family and social support (2)	Children in single-parent households: Percent of children (0-17yr) that live in household headed by single parent
	Children in poverty: The number of children (0-17yr) who live below the poverty threshold. The percent is based on the number of children (0-17yr) for whom poverty status was determined.
**Co-morbidities**	
AIDS	Co-morbidity measures are assigned using the AHRQ co-morbidity software. The AHRQ co-morbidity measures identify coexisting medical conditions that are not directly related to the principal diagnosis, or the main reason for admission, and are likely to have originated prior to the hospital stay. Co-morbidities are identified using ICD-9-CM diagnoses and the Diagnosis Related Group (DRG) in effect on the discharge date. The prefix "CM_" has been added to the AHRQ co-morbidity software data element names to distinguish the co-morbidity measures from other HCUP data elements. For more information, please refer to the materials available on the Tools and Software page of the HCUP User Support Website (http://www.hcup-us.ahrq.gov/tools_software.jsp).
Alcohol	
Rheumatoid arthritis/collagen vascular diseases	
Chronic blood loss anemia	
Congestive Heart Failure	
Chronic pulmonary disease	
Coagulopathy	
Depression	

Copyright © 2014 by The Boston Consulting Group, Inc. All rights reserved.

Figure D: Overview of 81 potential drivers investigated for PQIs (II of III)

**Co-morbidities (cont'd)**	
Diabetes, uncomplicated	
Diabetes with chronic complications	
Drug abuse	
Hypertension (complicated/uncomplicated)	
Hypothyroidism	
Liver disease	
Lymphoma	
Fluid and electrolyte disorders	
Metastatic cancer	
Other neurological disorders	
Paralysis	
Peripheral vascular disorders	
Psychoses	
Pulmonary circulation disorders	
Renal failure	
Solid tumor without metastasis	
Peptic ulcer disease (excluding bleeding)	
Valvular disease	
Weight loss	
**Health system factors**	
Size of county	Square miles (ArcGIS by ESRI)
County population	Use ZIP to HSA conversion to account for all the ZIPs encompassed within HSA. Add up population of these ZIP codes based on 2010 Census data
County population density	County population/Size of the county
Pollution	Drinking water violations: Percent of population potentially exposed to water exceeding a violation limit during the past year
Rural/Urban	T2013 Rural-Urban Continuum Codes (http://www.ers.usda.gov/data-products/rural-urban-continuum-codes.aspx)
Post Acute Care costs (3)	Per capita post-acute care cost for each of the following:<br>• Skilled nursing care<br>• Home health<br>• Hospice
Total cost (2)	• Total Standardized Risk-Adjusted Costs<br>• Standardized Risk-Adjusted Per Capita Costs

Copyright © 2014 by The Boston Consulting Group, Inc. All rights reserved.

Figure D: Overview of 81 potential drivers investigated for PQIs (III of III)

**Health system factors cont'd**	
Inpatient costs (2)	• IP Standardized Costs<br>• IP Per Capita Standardized Costs
Outpatient cost (2)	• OP Standardized Costs<br>• OP Per Capita Standardized Costs
Inpatient utilization (3)	• IP Covered Stays Per 1000 Beneficiaries<br>• IP Covered Days Per 1000 Beneficiaries<br>• Emergency Department Visits per 1000 Beneficiaries
Post acute care utilization costs (6)	Utilization of SNF, hospice, home health for each of the following:<br>• Post acute users (with a covered stay)<br>• Covered Stays Per 1000 Beneficiaries
Outpatient care/utilization (2)	• # OP Users<br>• OP Visits Per 1000 Beneficiaries
PCP number	Sum of doctors with the following primary specialties: Family practice, General practice, Internal medicine, Preventative medicine, Pediatric medicine
PCP concentration	Number of PCPs per 100,000 of population in each county
PCP % Affiliated	Percent of total PCPs who belong to group practice in each county. If a physician has a Unique Group Practice ID assigned by PECOS to the Group Practice it will be counted as a physician who belongs to group practice.
Specialists - all	Sum of physicians with the following primary specialties : Cardiac electrophysiology, Cardiovascular disease (cardiology), Endocrinology, Dermatology, Gastroenterology, Gynecological Oncology, Hematology, Hematology/Oncology, Infectious Disease, Neurology, Medical Oncology, Nephrology, Pulmonary Disease, Rheumatology
Total number of surgeons	Sum of physicians with the following primary specialties: Cardiac Surgery, Colorectal Surgery (Proctology), General Surgery, Vascular Surgery, Thoracic Surgery, Surgical Oncology, Neurosurgery, Orthopedic Surgery, Urology, Obstetrics/Gynecology, Ophthalmology
Physicians Acute care	Total sum of Physicians in county
Payer Type (5)	Percent of records at hospital with each of the primary payers: Medicare, Medicaid, private, self pay, no charge

Copyright © 2014 by The Boston Consulting Group, Inc. All rights reserved.

Draft—for discussion only

Figure D: Footnote and methods

Overview of 81 potential drivers investigated for PQIs

Summary and definitions of 81 potential drivers investigated for IQIs and PSIs. We assembled a database from 14 reputable sources of potential drivers, including population factors such as demographics, lifestyle, and socioeconomics, as well as co-morbidities and health system factors (such as physician supply and hospital bed supply). Each factor was linked at the county level.

Copyright © 2014 by The Boston Consulting Group, Inc. All rights reserved.

Figure E: Comparison of ratios with Normal and Poisson distribution

	Gaussian Distribution County/Hospital level (Top/bottom 10%)				Poisson Distribution County/Hospital level(Top/bottom 10%)			
	Observed[1]	+ Pop. factors adjusted	+ Co-morb. -adjusted	+ System factor adjusted	Observed[1]	+ Pop. factors adjusted	+ Co-morb. -adjusted	+ System factor adjusted
**Acute mortality (IQI)**								
15 Acute Myocardial Infarction (AMI) Mortality	4.0	3.5	2.7	2.3	4	3.8	2.9	2.7
16 Congestive Heart Failure (CHF) Mortality	4.1	3.7	2.8	2.7	4.1	3.8	2.9	2.7
17 Acute Stroke Mortality	2.5	2.5	2.3	2.3	2.6	3.0	2.7	2.6
18 Gastrointestinal Hemorrhage Mortality	1.9	1.8	1.7	1.6	1.9	1.9	1.8	1.8
19 Hip Fracture Mortality	2.0	1.9	1.8	1.7	2	2.0	1.9	1.9
20 Pneumonia Mortality	2.8	2.7	2.3	2.2	2.8	2.6	2.4	2.3
**Acute safety (PSI)**								
03 Pressure Ulcer Rate	61.2	57.4	49.3	45.4	61.2	59.7	64.8	69.4
06 Iatrogenic Pneumothorax Rate	2.4	2.4	2.3	2.2	2.4	2.5	2.5	2.4
07 Central Venous Catheter Bloodstream Infection	23.5	22.4	19.0	18.3	23.5	21.6	22.4	21.7
08 Postoperative Hip Fracture Rate	2.2	2.1	2.0	1.8	2.2	2.3	3.3	3.2
12 Postop. Pulmonary Embolism or DVT	4.6	4.3	3.8	3.4	4.6	4.6	4.3	4.4
13 Postoperative Sepsis Rate	3.9	3.8	3.4	3.2	3.9	4.3	4.0	4.0
14 Postoperative Wound Dehiscence Rate	2.2	2.2	2.2	2.1	2.2	2.3	2.3	2.3
15 Accidental Puncture or Laceration Rate	5.8	5.4	4.2	3.6	5.8	5.5	4.7	3.9
**Prevention (PQI)**								
01 Diabetes Short-Term Complic. Admission Rate	7.7	5.3	5.0	5.1	7.7	5.5	5.4	5.2
03 Diabetes Long-Term Complic. Admission Rate	4.8	3.5	3.3	2.9	4.8	3.5	3.3	3.1
05 COPD or Asthma in Older Adults	4.9	3.5	3.0	2.6	4.9	3.1	2.8	2.4
08 Congestive Heart Failure (CHF) Admission Rate	4.3	2.6	2.4	2.2	4.3	2.7	2.5	2.2
10 Dehydration Admission Rate	4.6	3.3	3.4	3.0	4.6	3.3	3.2	2.8
11 Bacterial Pneumonia Admission Rate	4.6	2.7	2.7	2.4	4.6	2.6	2.4	2.3
12 Urinary Tract Infection Admission Rate	4.5	3.1	2.8	2.5	4.5	3.2	3.0	2.6
14 Uncontrolled Diabetes Admission Rate	*	*	*	*	*	*	*	*
15 Asthma in Younger Adults Admission Rate	*	*	*	*	*	*	*	*
16 Lower-Extremity Amputation Among Diabetics	*	*	*	*	*	*	*	*

1. For IQIs and PSIs all observed rates have Baysian noise correction (shrinkage)
*denominator effectively zero

Copyright © 2014 by The Boston Consulting Group, Inc. All rights reserved.

Figure E: Comparison of ratios with Normal and Poisson distribution

Comparison of outcomes variability, measured via D9/D1 ratio between risk adjustments conducted with a Gaussian distribution and Poisson distribution. Fields marked in red indicate that the two results are meaningfully (>25%) different

(Footnote and methods on a separate slide)

Copyright © 2014 by The Boston Consulting Group, Inc. All rights reserved.

Figure F: HSA level geographic variability in IQI 15 - Acute Myocardial Infarction (AMI) Mortality Rate

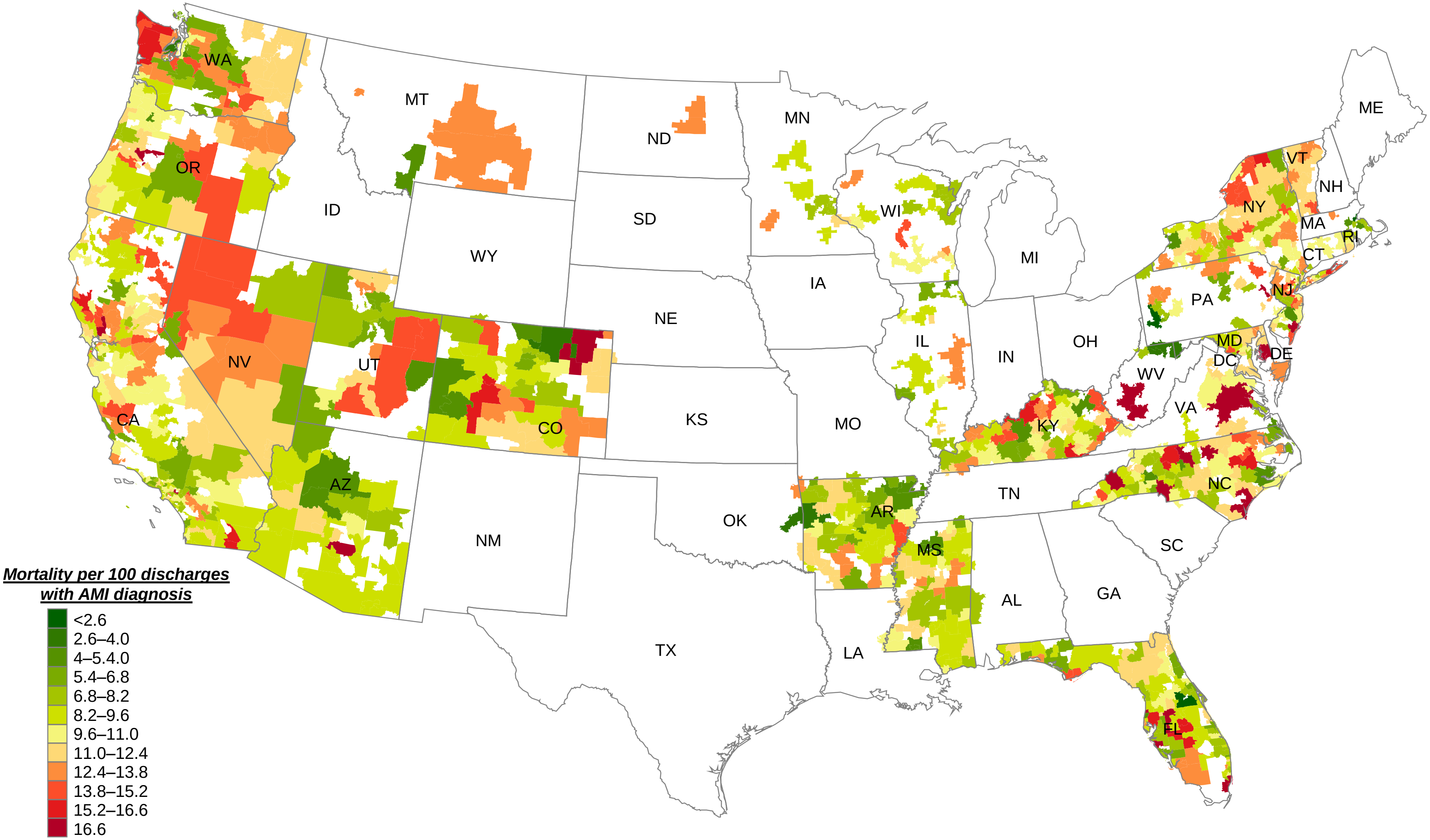

Copyright © 2014 by The Boston Consulting Group, Inc. All rights reserved.

Figure F: HRR level geographic variability in IQI 15 - Acute Myocardial Infarction (AMI) Mortality Rate

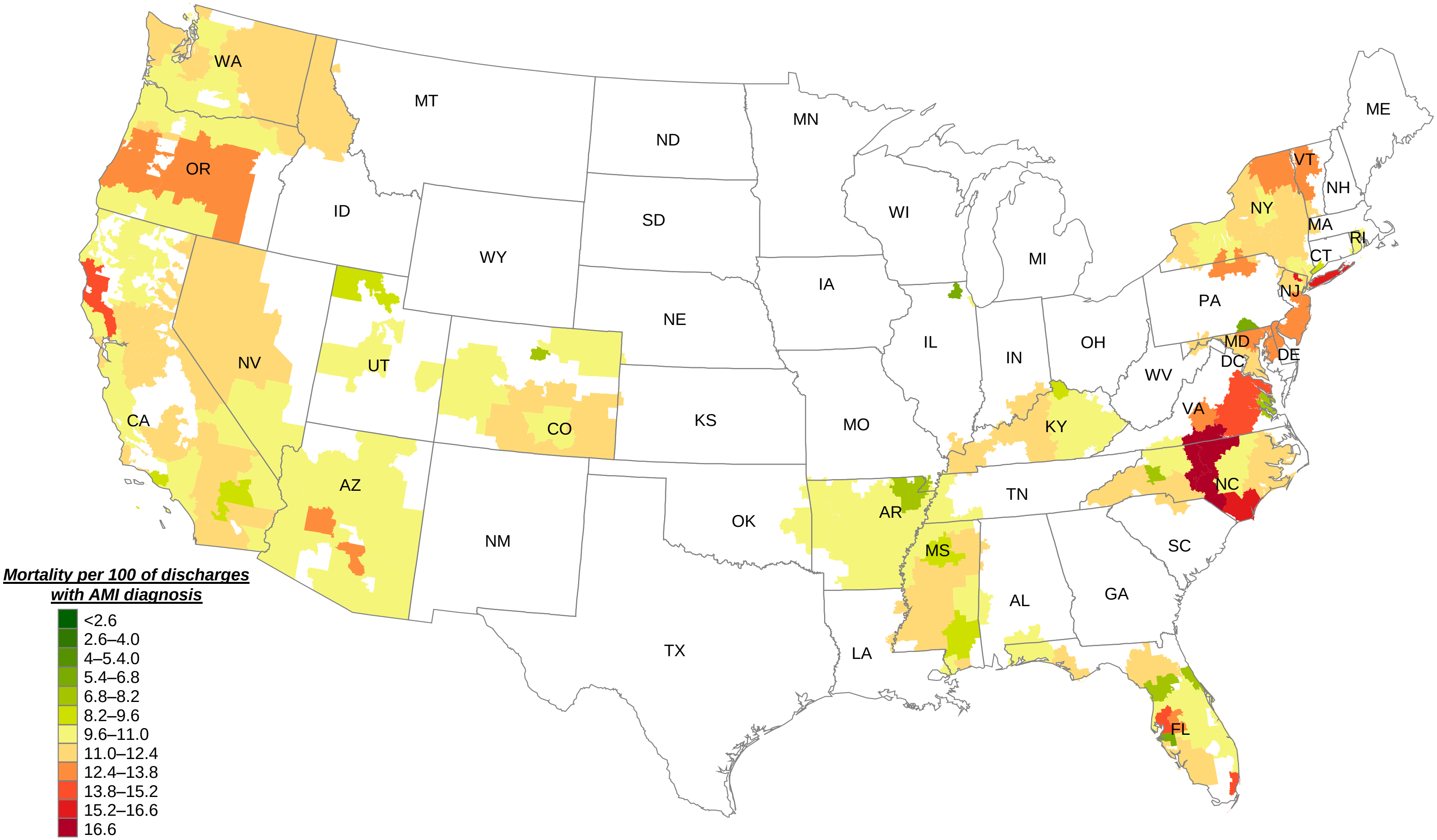

Copyright © 2014 by The Boston Consulting Group, Inc. All rights reserved.

Figure F: State level geographic variability in IQI 15 - Acute Myocardial Infarction (AMI) Mortality Rate

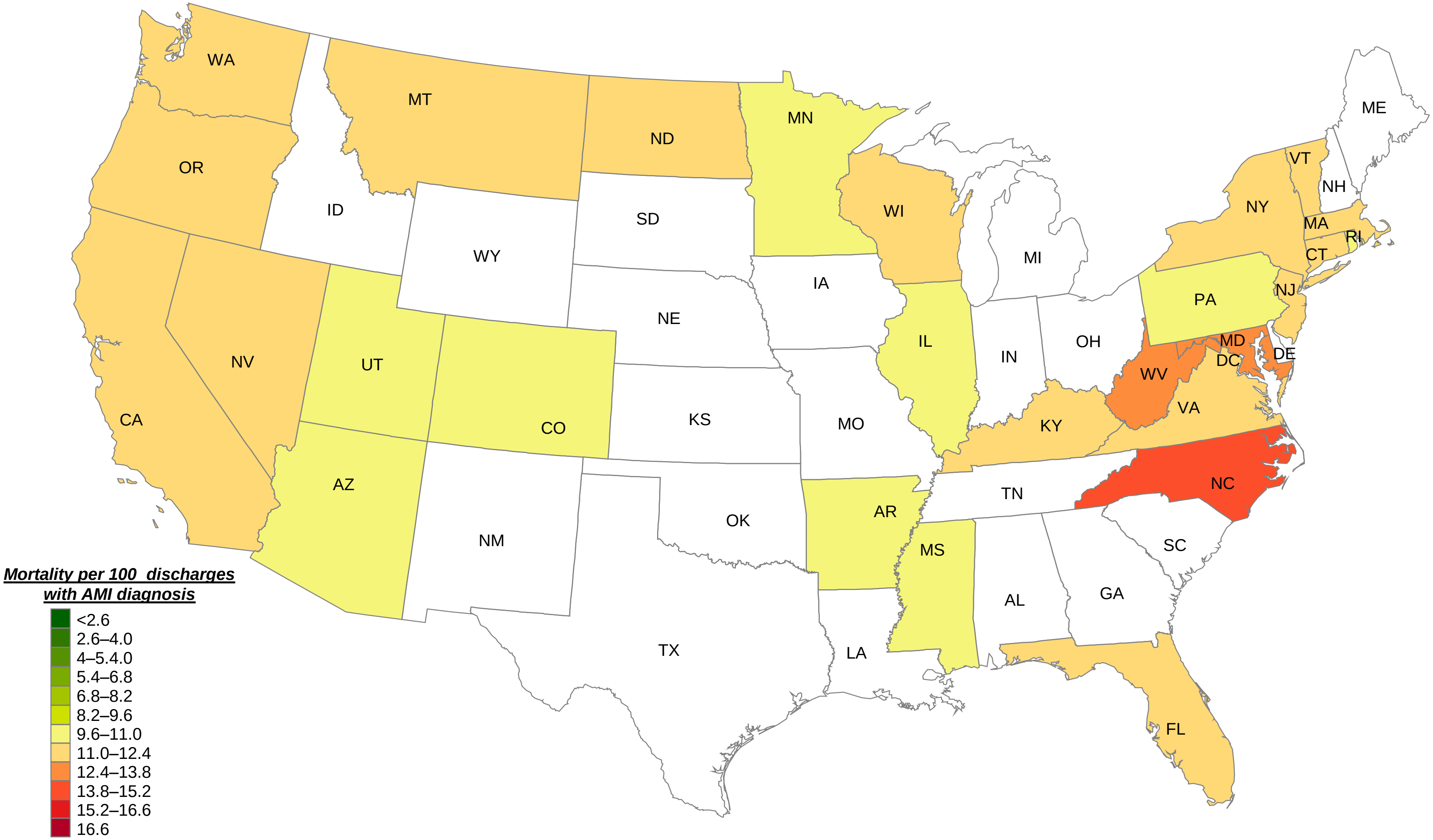

Copyright © 2014 by The Boston Consulting Group, Inc. All rights reserved.

Draft—for discussion only

Figure F: HSA level geographic variability in IQI 16 - Congestive Heart Failure (CHF) Mortality Rate

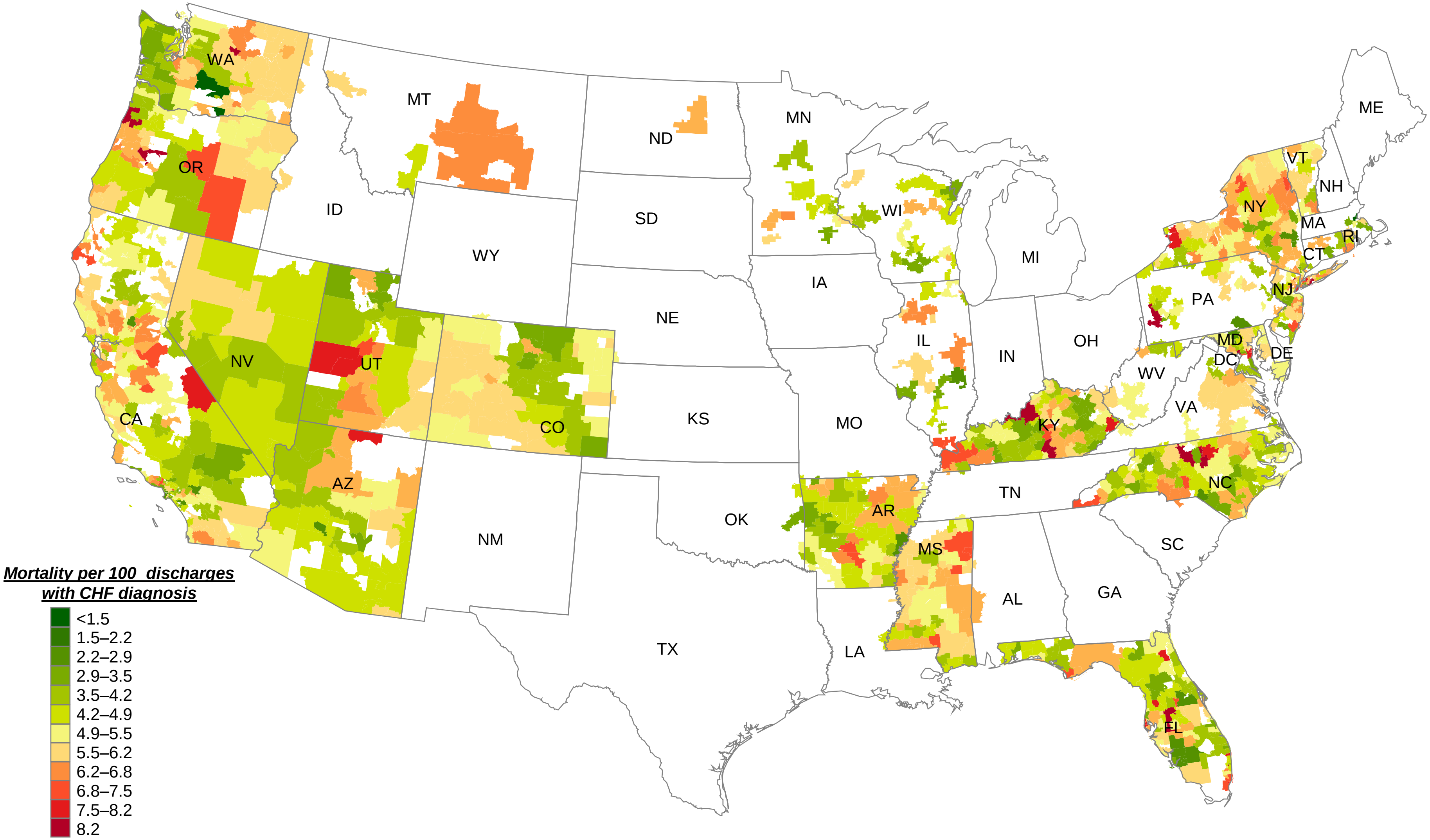

Copyright © 2014 by The Boston Consulting Group, Inc. All rights reserved.

Draft—for discussion only

Figure F: HRR level geographic variability in IQI 16 - Congestive Heart Failure (CHF) Mortality Rate

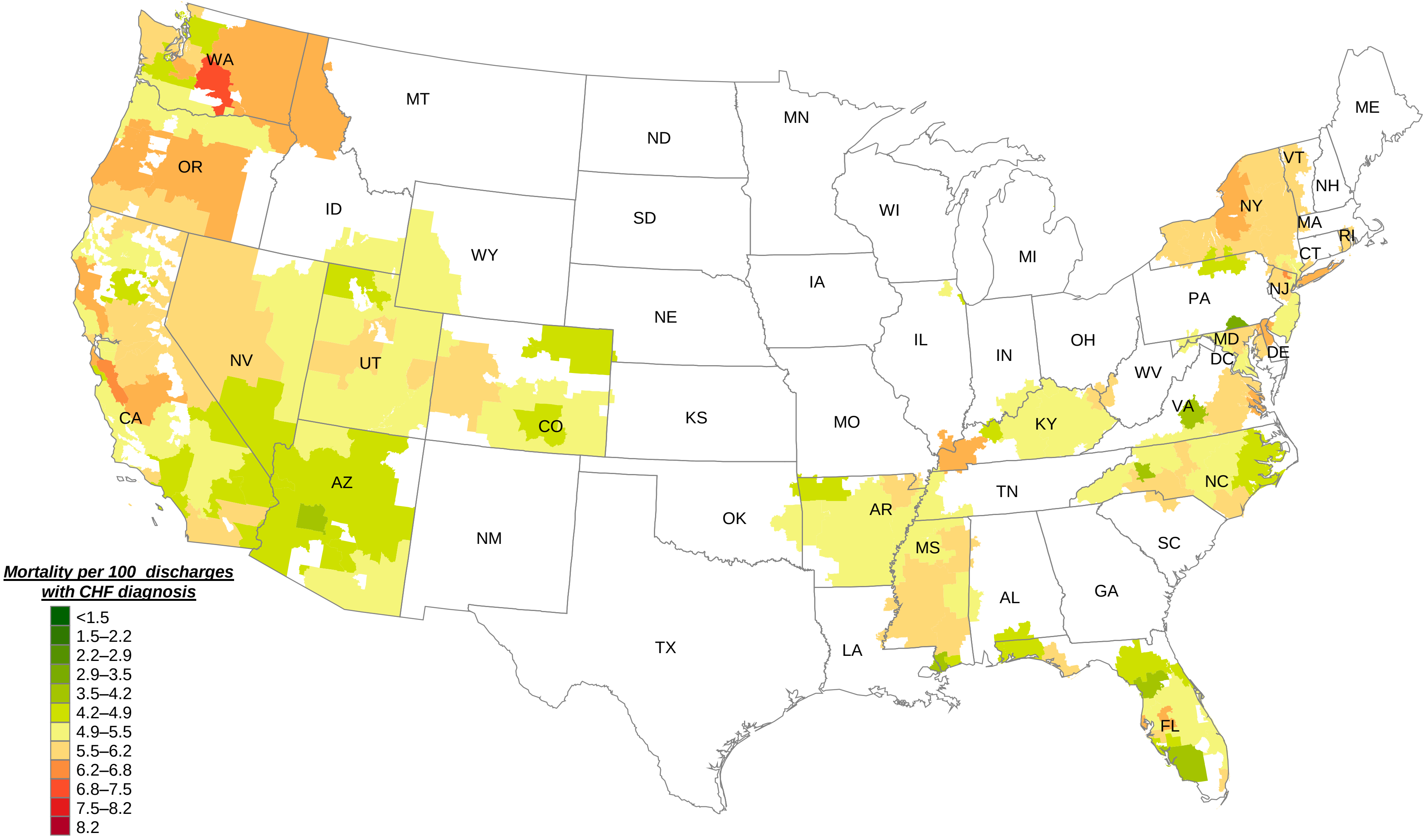

Copyright © 2014 by The Boston Consulting Group, Inc. All rights reserved.

Figure F: State level geographic variability in IQI 16 - Congestive Heart Failure (CHF) Mortality Rate

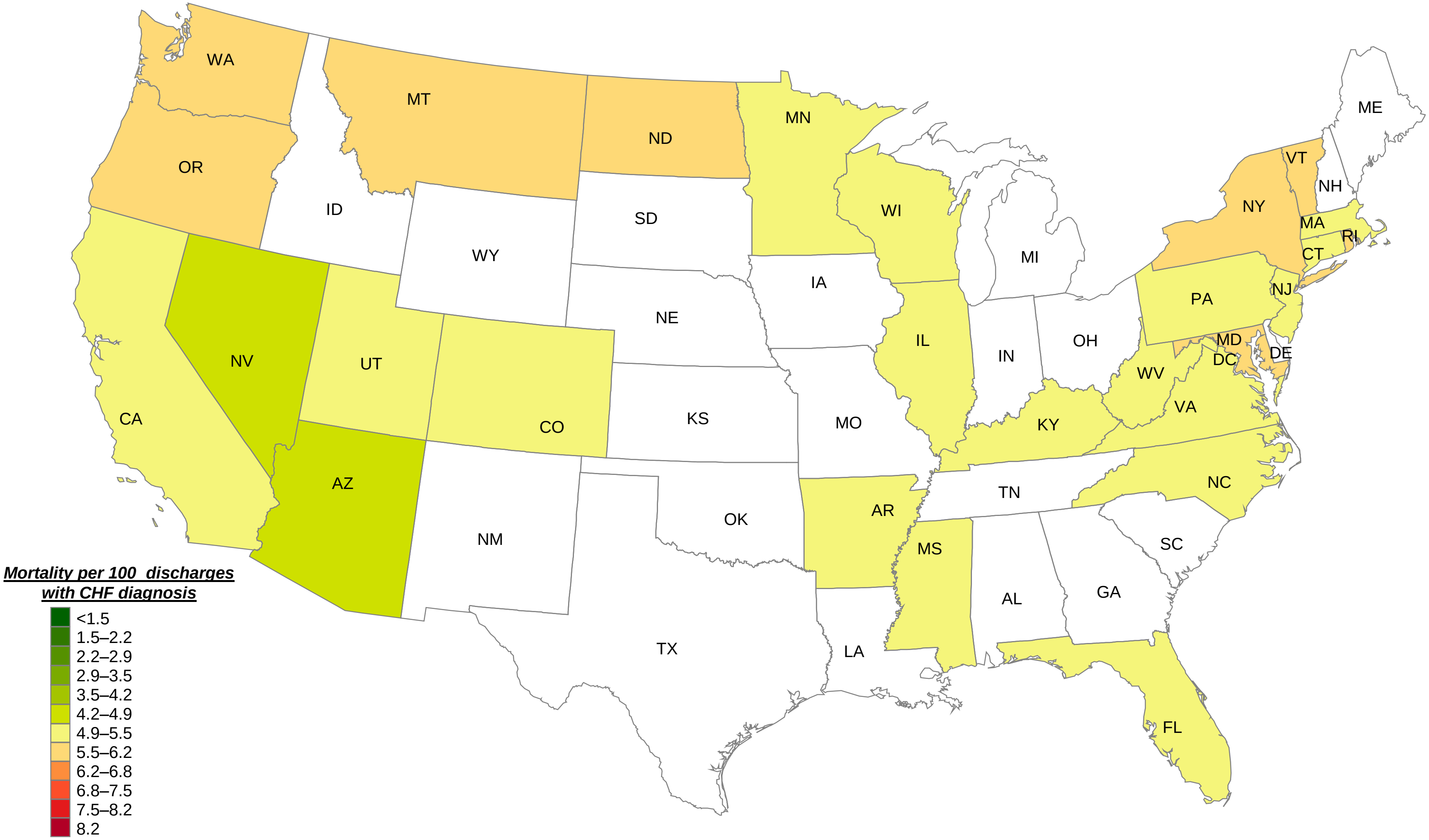

Copyright © 2014 by The Boston Consulting Group, Inc. All rights reserved.

Draft—for discussion only

Figure F: HSA level geographic variability in IQI 17 - Acute Stroke Mortality Rate

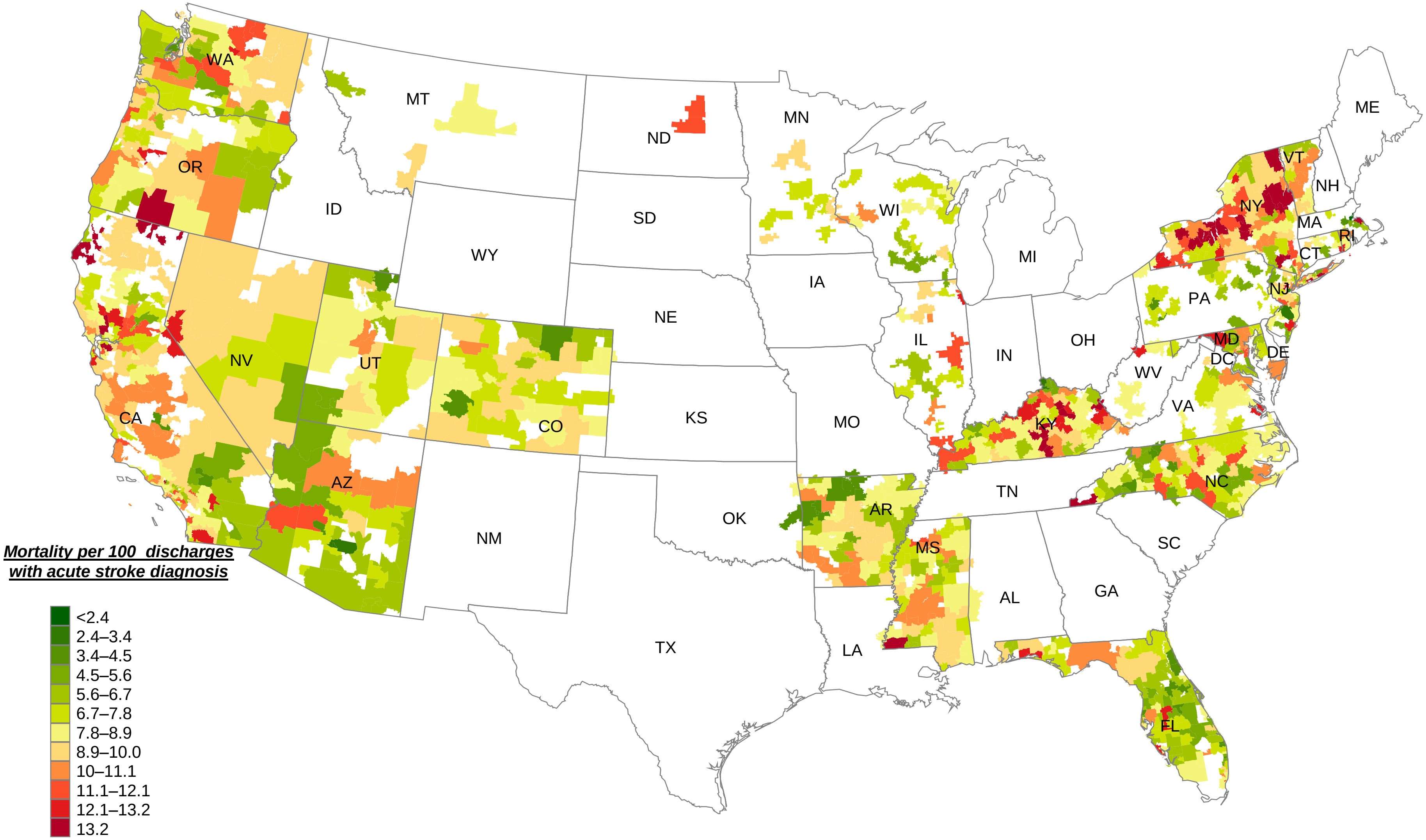

Copyright © 2014 by The Boston Consulting Group, Inc. All rights reserved.

Figure F: HRR level geographic variability in IQI 17 - Acute Stroke Mortality Rate

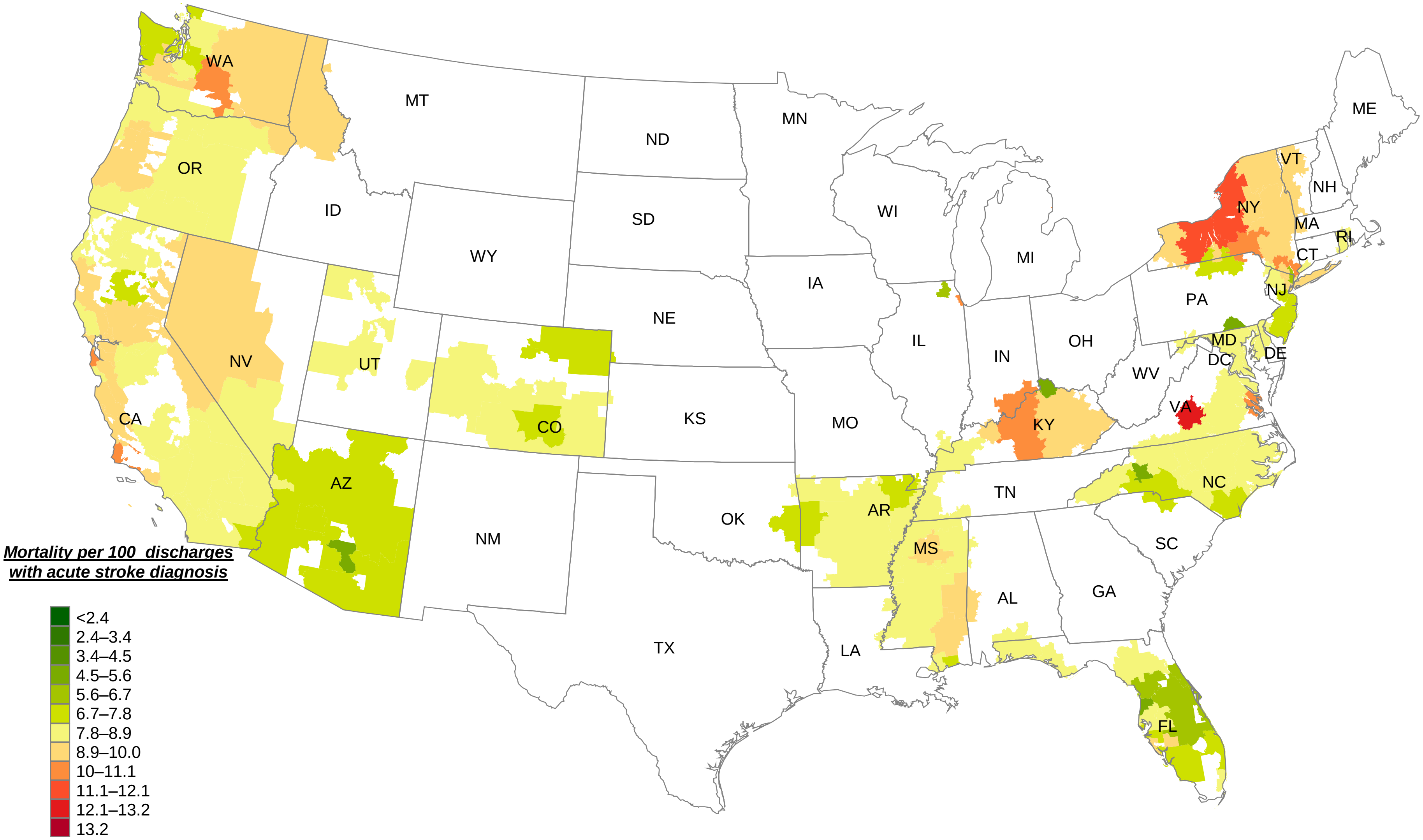

Copyright © 2014 by The Boston Consulting Group, Inc. All rights reserved.

Figure F: State level geographic variability in IQI 17 - Acute Stroke Mortality Rate

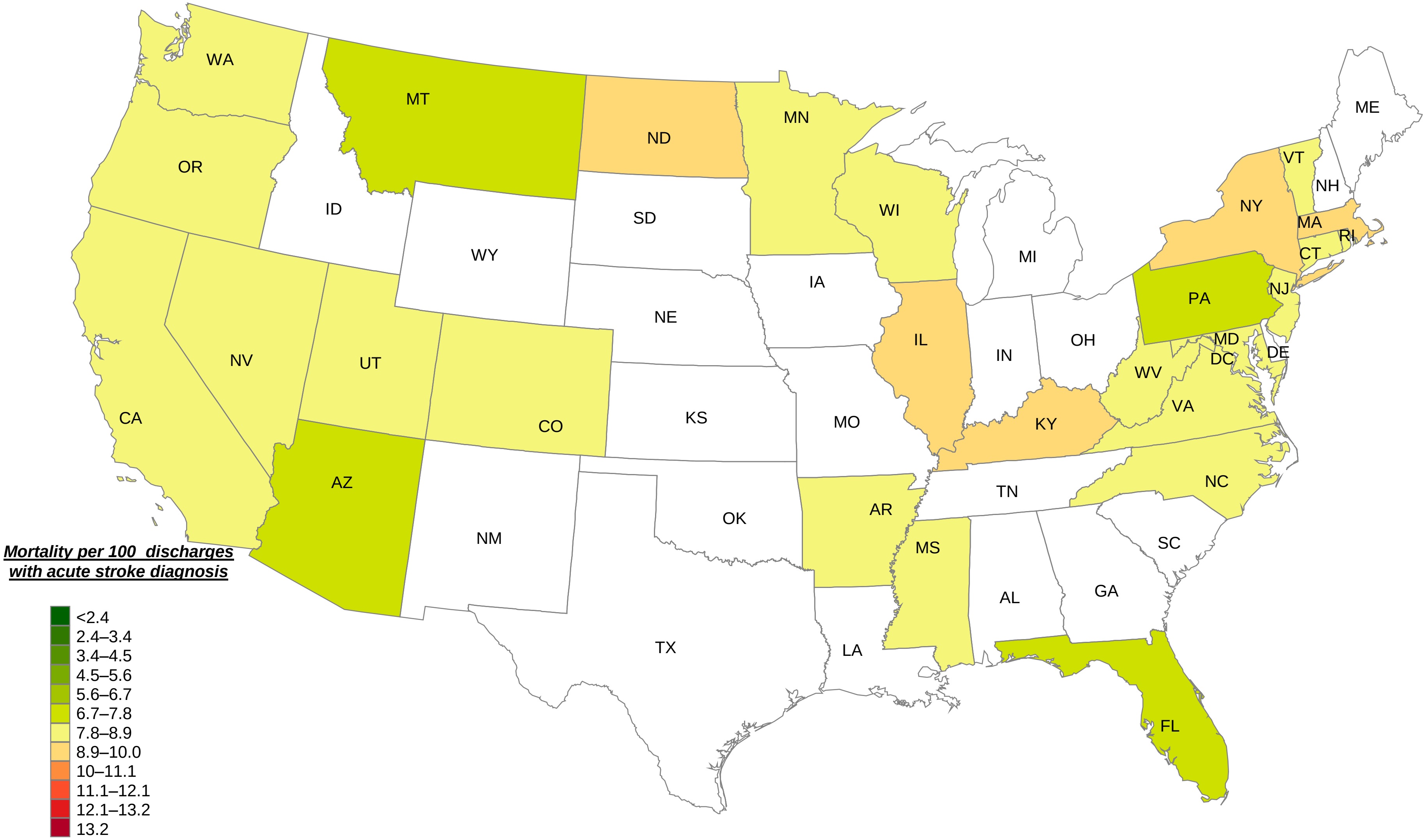

Copyright © 2014 by The Boston Consulting Group, Inc. All rights reserved.

Figure F: HSA level geographic variability in IQI 18 - Gastrointestinal Hemorrhage Mortality Rate

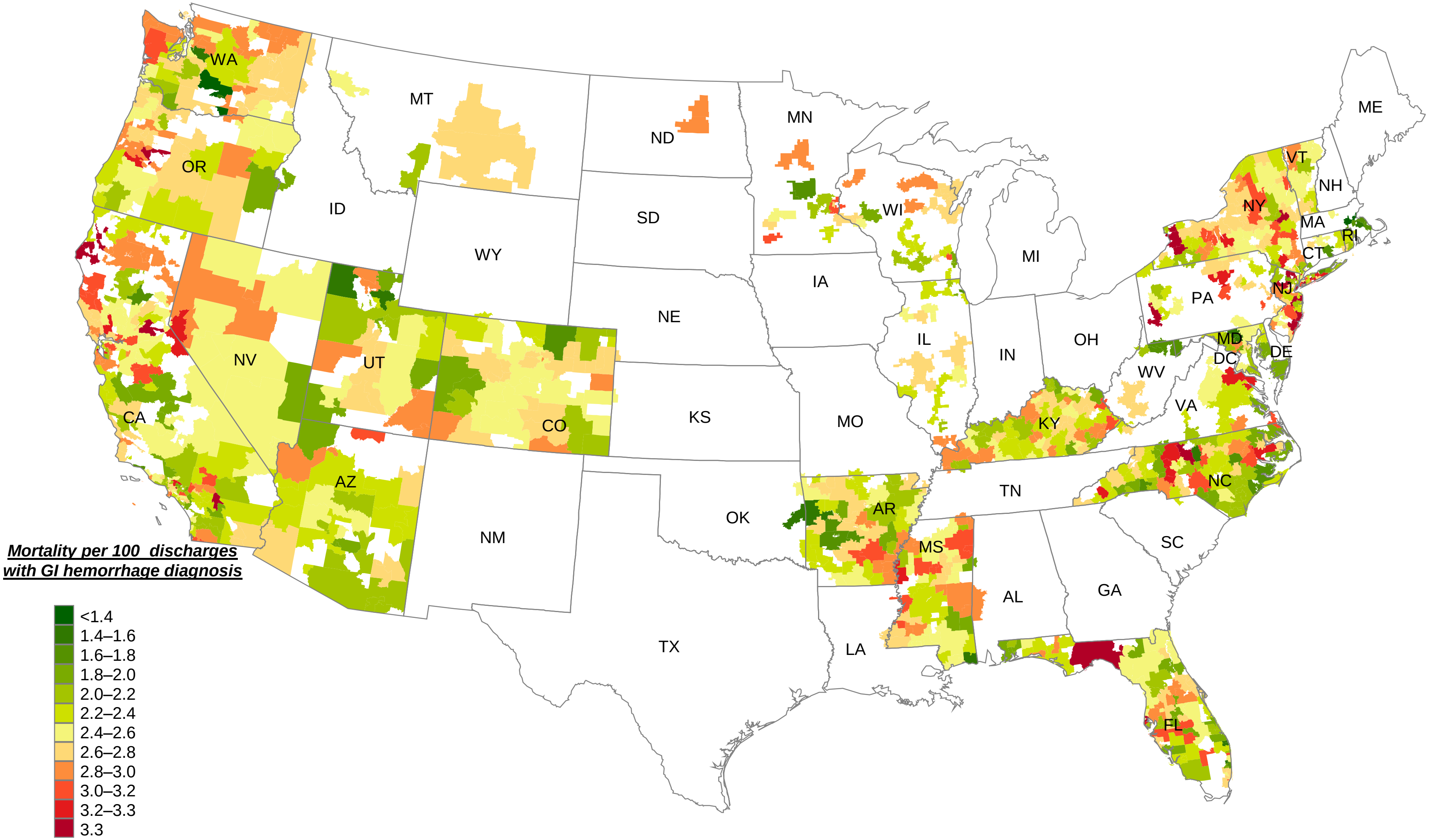

Copyright © 2014 by The Boston Consulting Group, Inc. All rights reserved.

Figure F: HRR level geographic variability in IQI 18 - Gastrointestinal Hemorrhage Mortality Rate

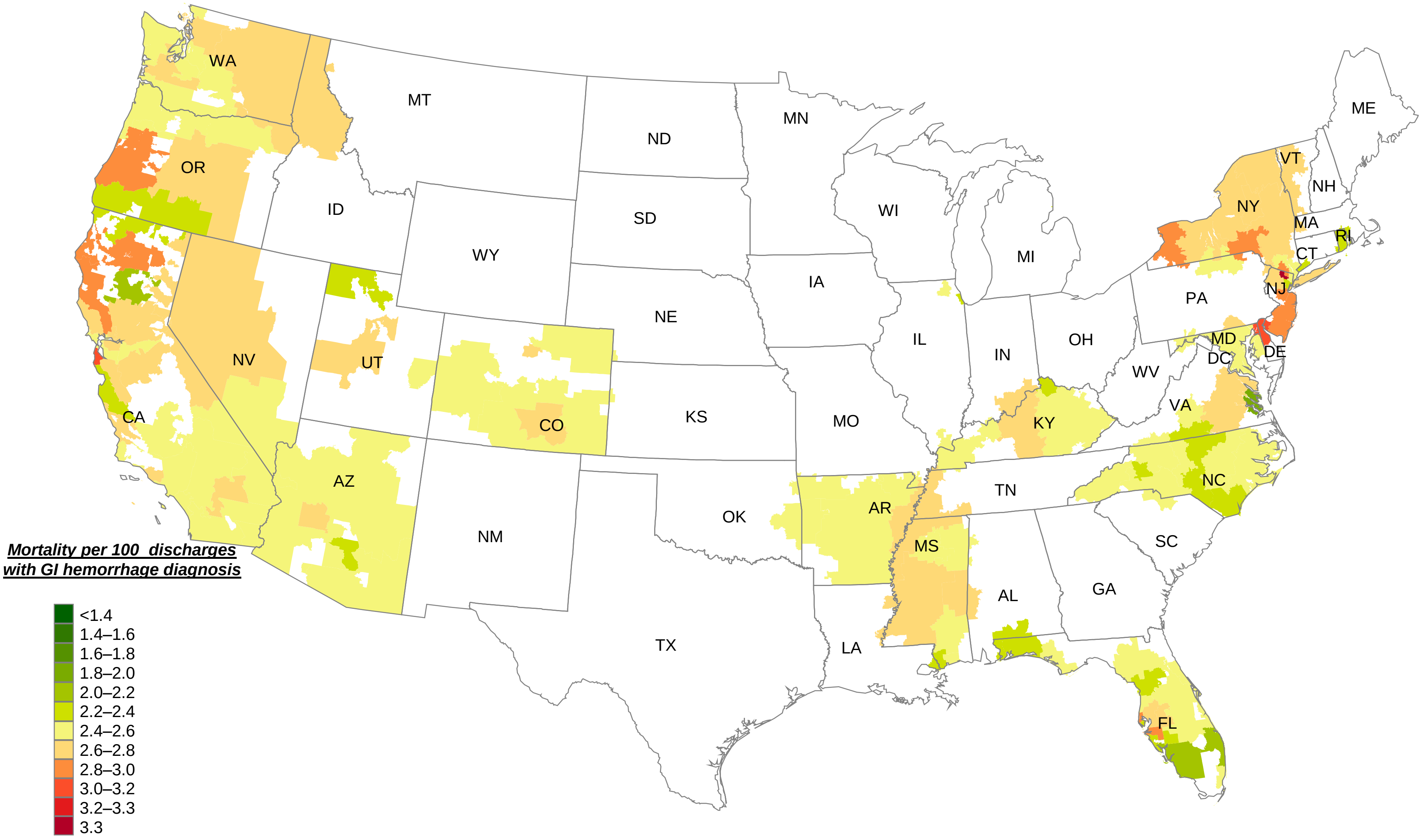

Copyright © 2014 by The Boston Consulting Group, Inc. All rights reserved.

Figure F: State level geographic variability in IQI 18 - Gastrointestinal Hemorrhage Mortality Rate

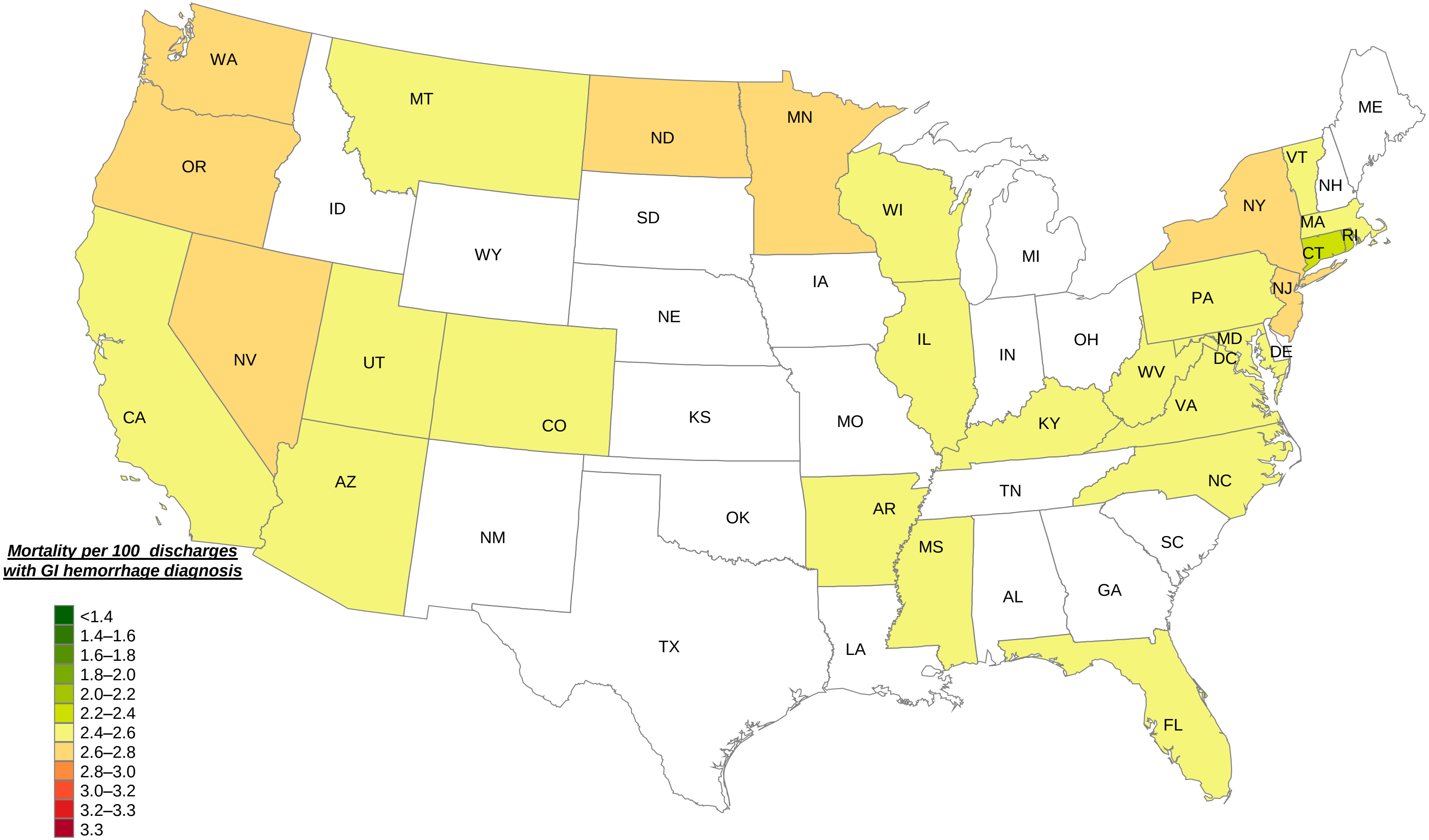

Copyright © 2014 by The Boston Consulting Group, Inc. All rights reserved.

Draft—for discussion only

Figure F: HSA level geographic variability in IQI 19 - Hip Fracture Mortality Rate

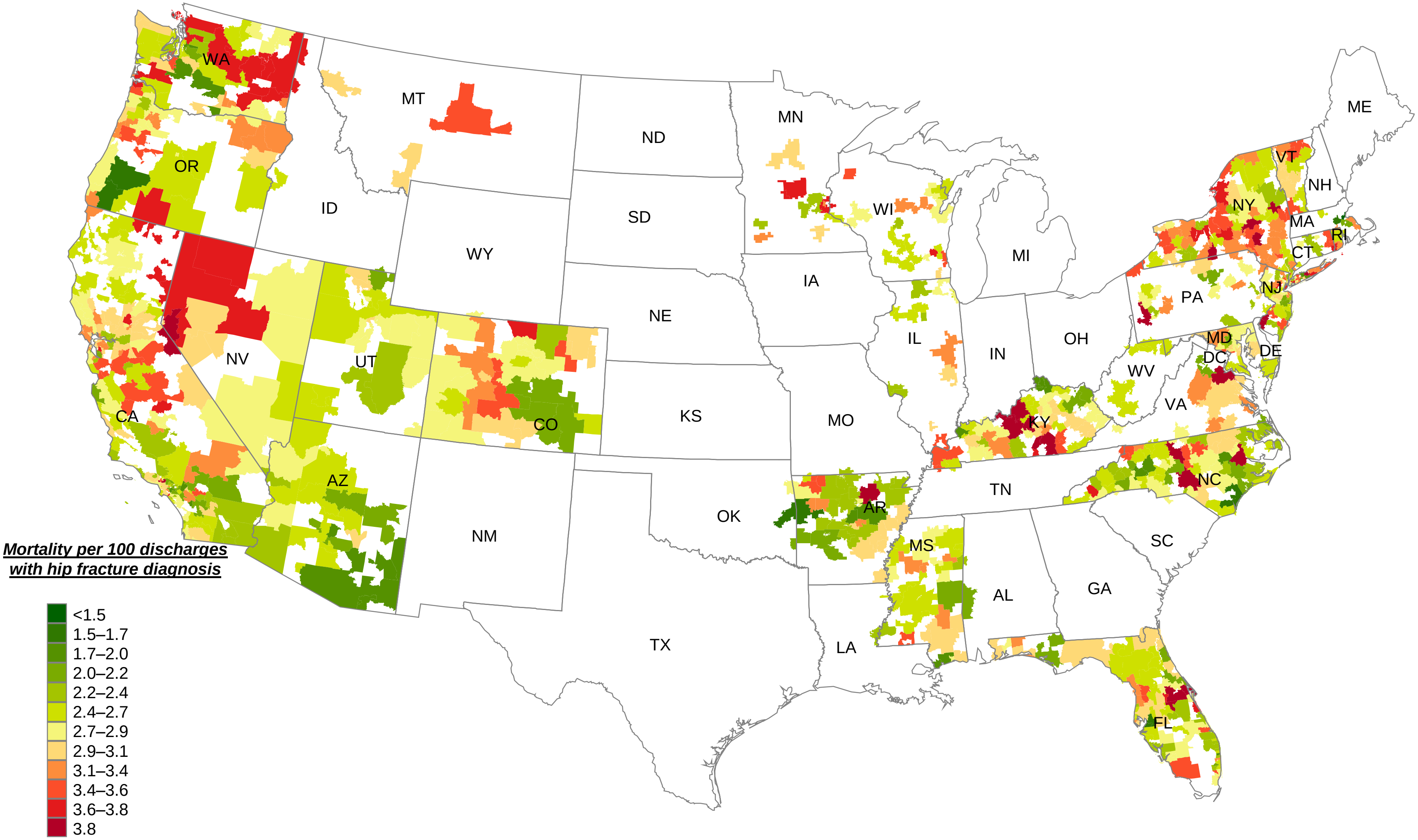

Copyright © 2014 by The Boston Consulting Group, Inc. All rights reserved.

Draft—for discussion only

Figure F: HRR level geographic variability in IQI 19 - Hip Fracture Mortality Rate

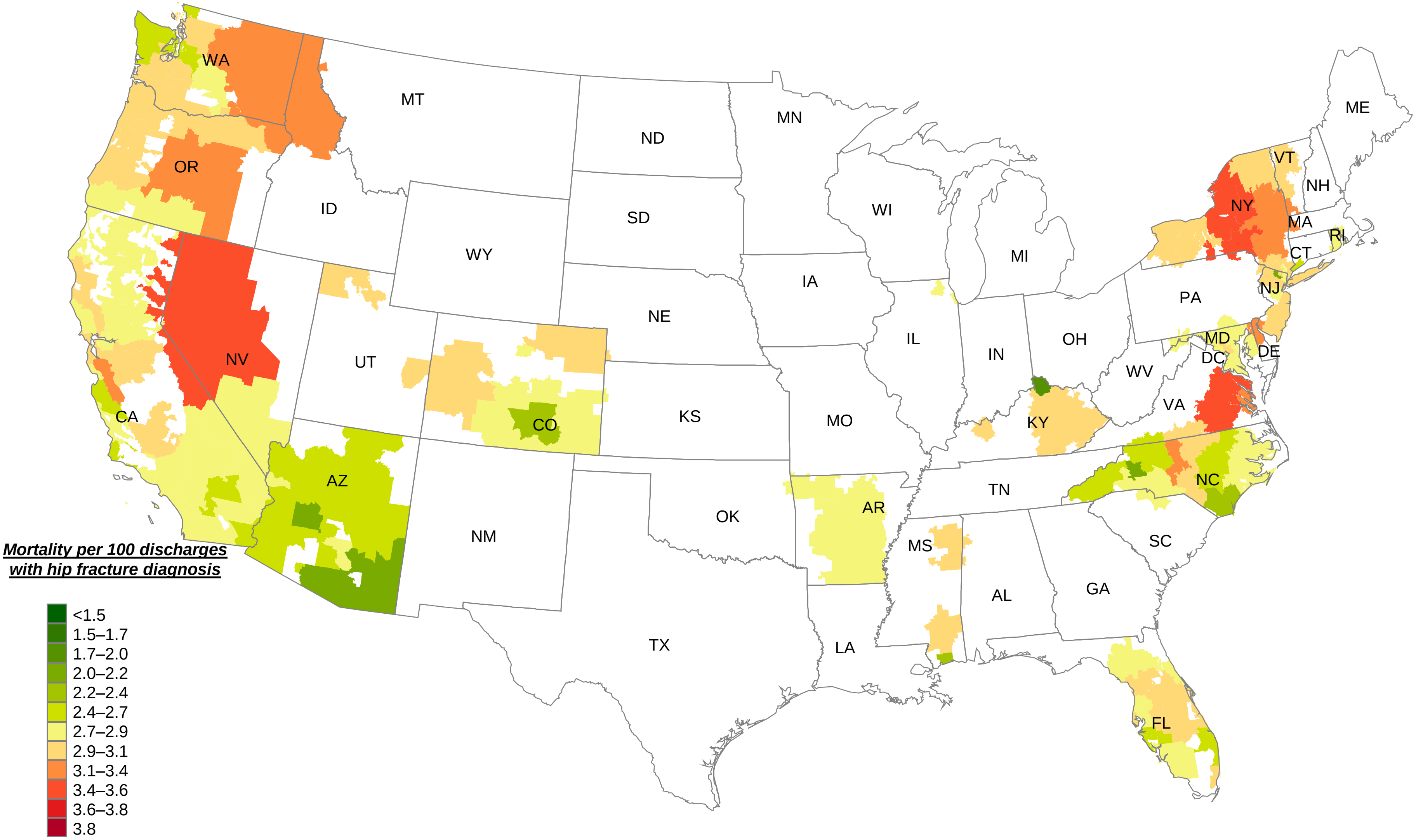

Copyright © 2014 by The Boston Consulting Group, Inc. All rights reserved.

Draft—for discussion only

Figure F: State level geographic variability in IQI 19 - Hip Fracture Mortality Rate

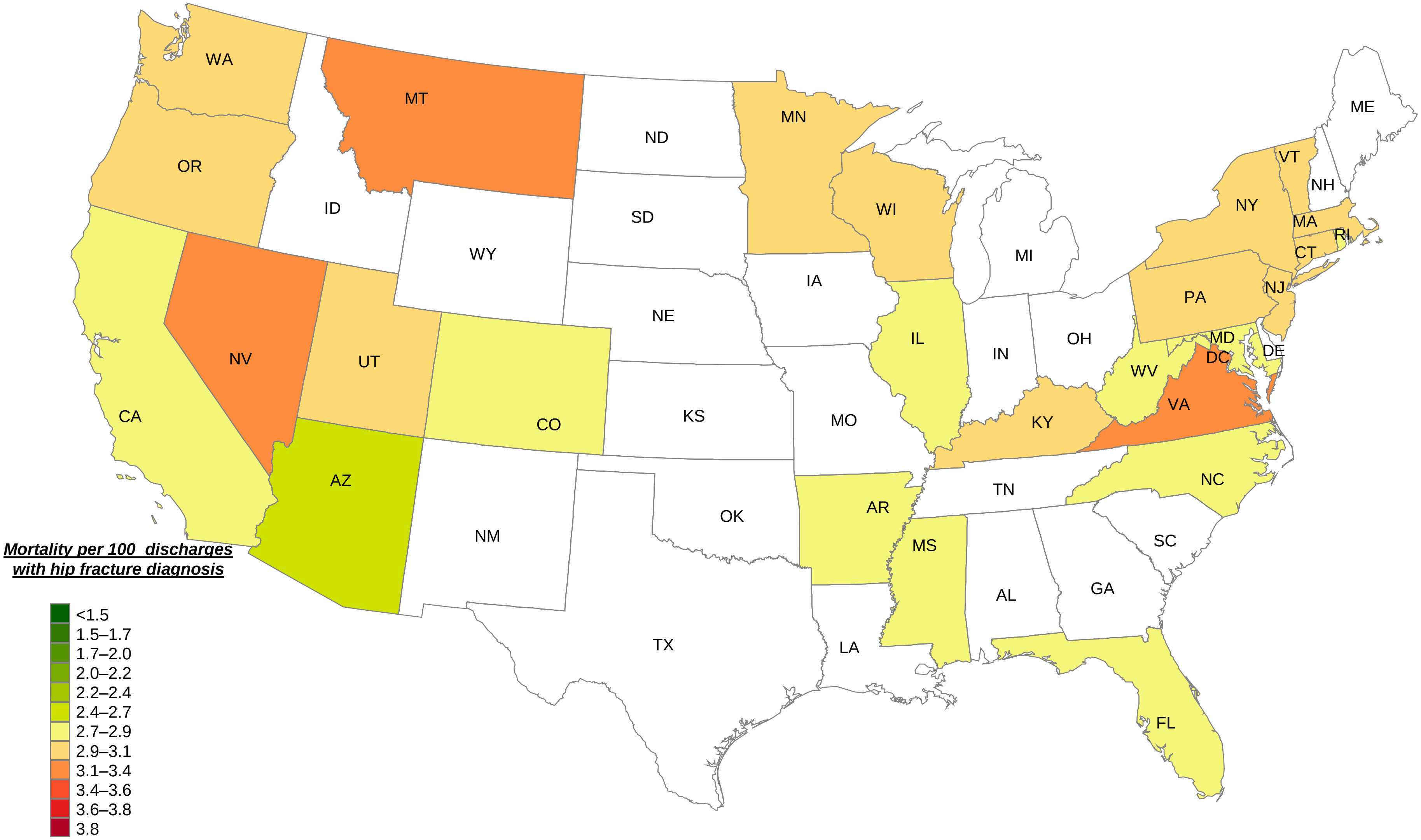

Copyright © 2014 by The Boston Consulting Group, Inc. All rights reserved.

Draft—for discussion only

Figure F: HSA level geographic variability in IQI 20 - Pneumonia Mortality Rate

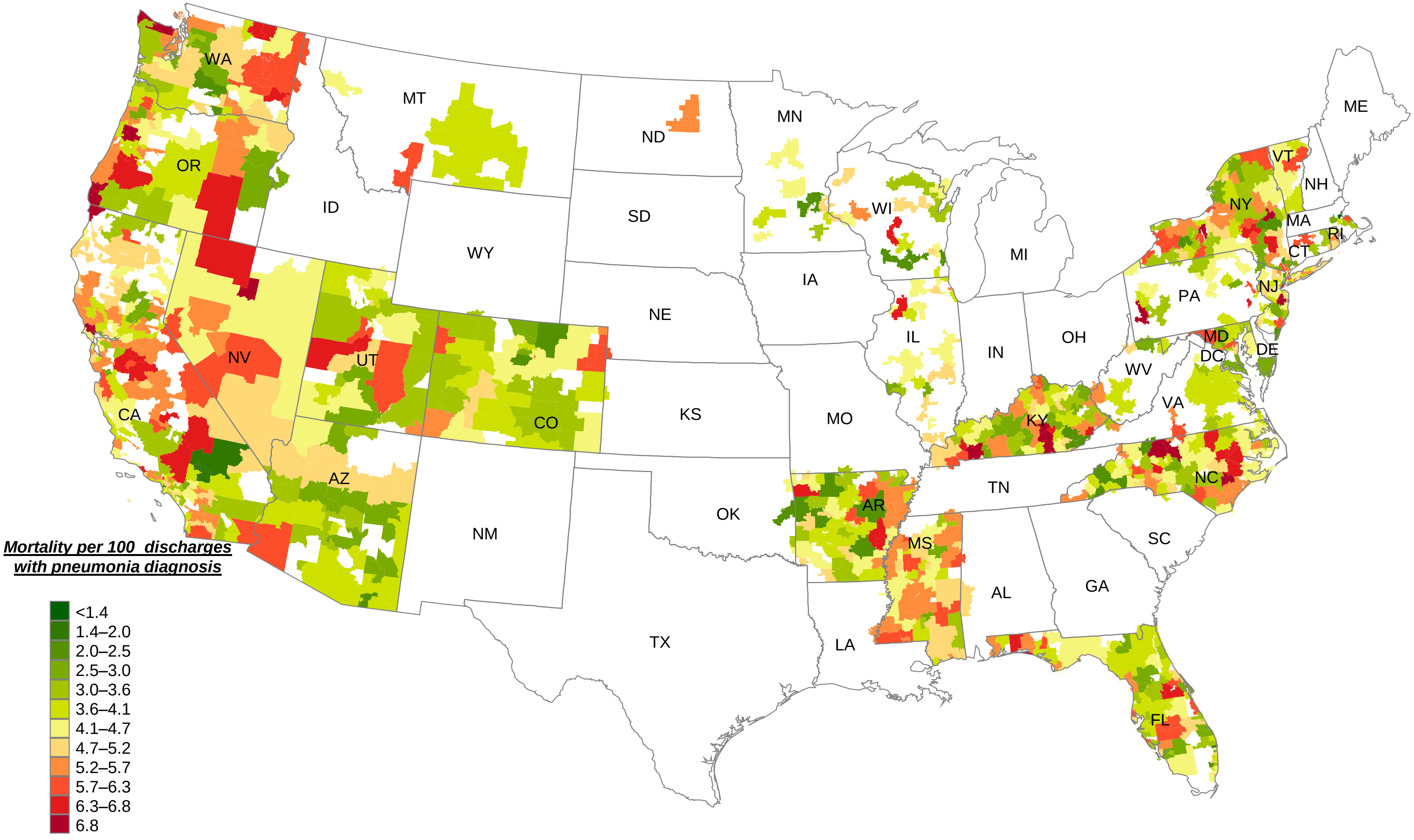

Copyright © 2014 by The Boston Consulting Group, Inc. All rights reserved.

Draft—for discussion only

Figure F: HRR level geographic variability in IQI 20 - Pneumonia Mortality Rate

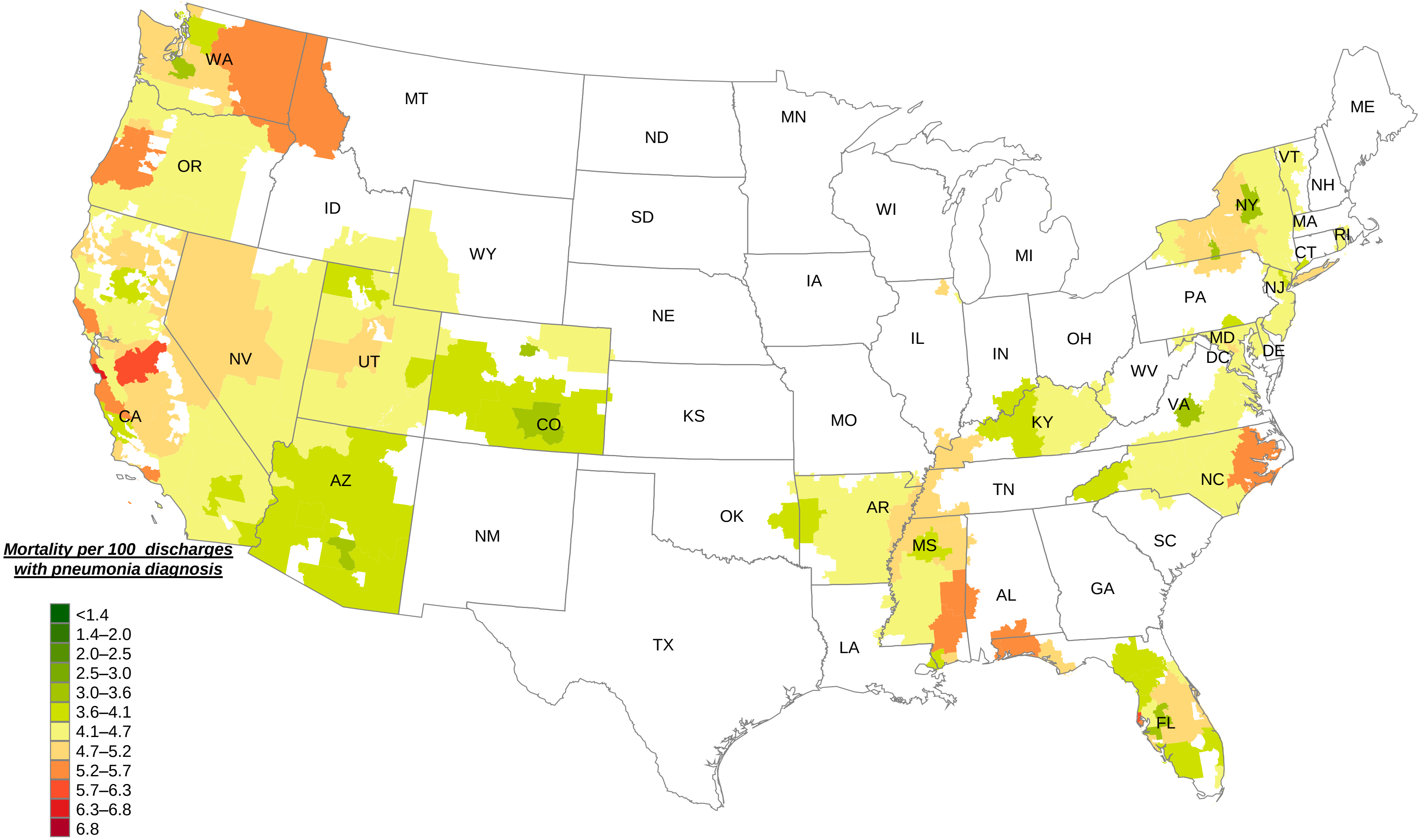

Copyright © 2014 by The Boston Consulting Group, Inc. All rights reserved.

Figure F: State level geographic variability in IQI 20 - Pneumonia Mortality Rate

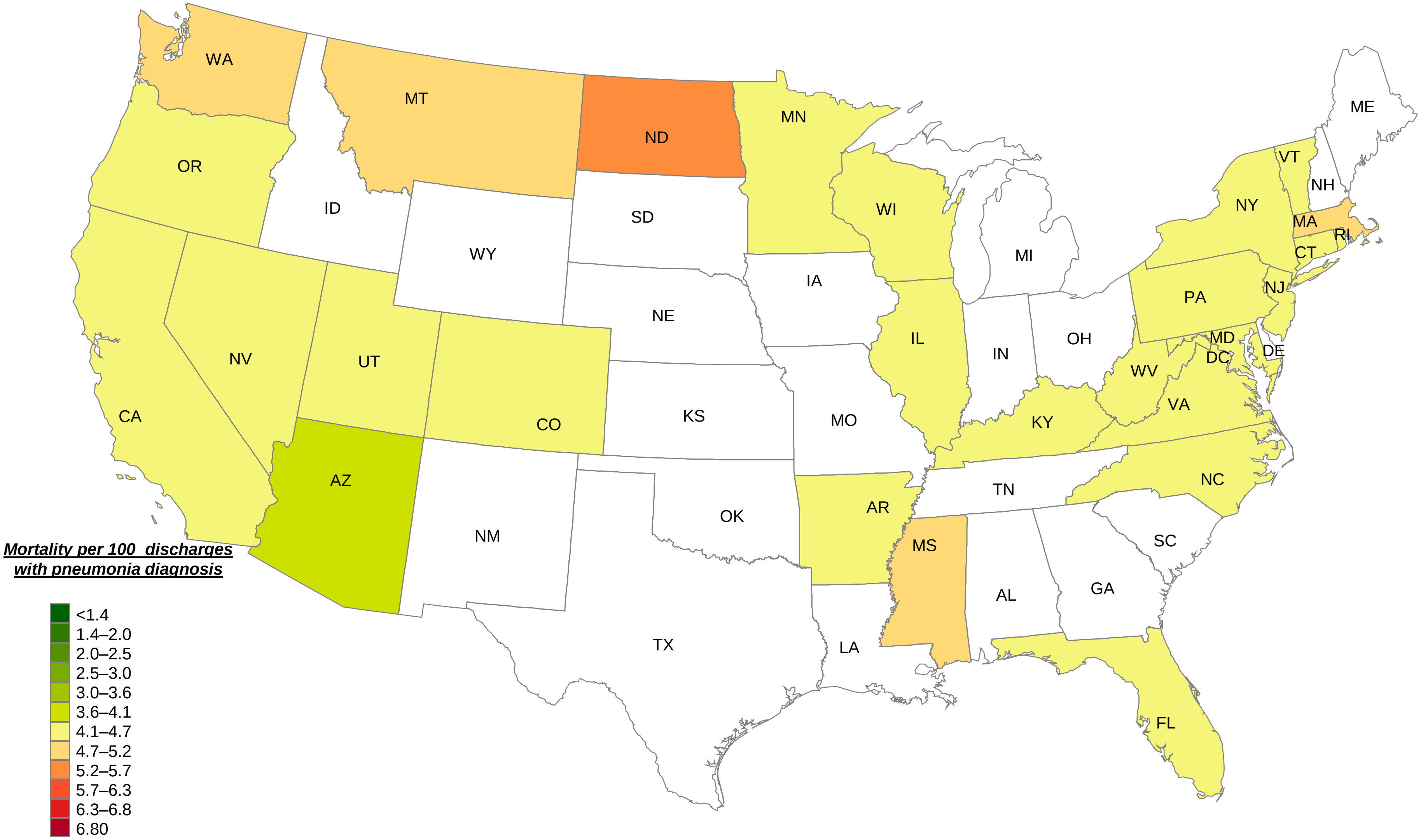

Copyright © 2014 by The Boston Consulting Group, Inc. All rights reserved.

Figure F: HSA level geographic variability in PSI 03 - Pressure Ulcer Rate

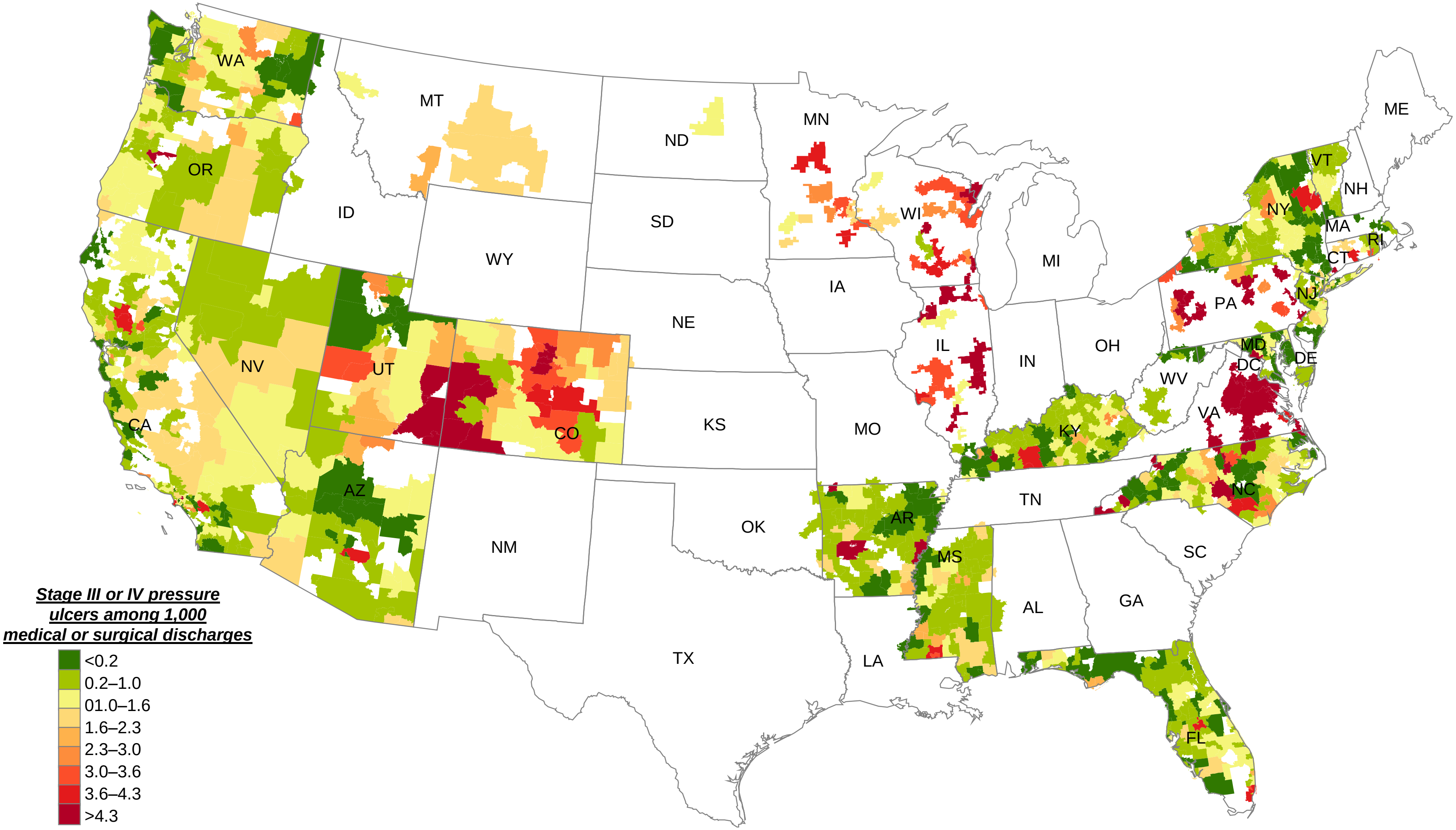

Copyright © 2014 by The Boston Consulting Group, Inc. All rights reserved.

Figure F: HRR level geographic variability in PSI 03 - Pressure Ulcer Rate

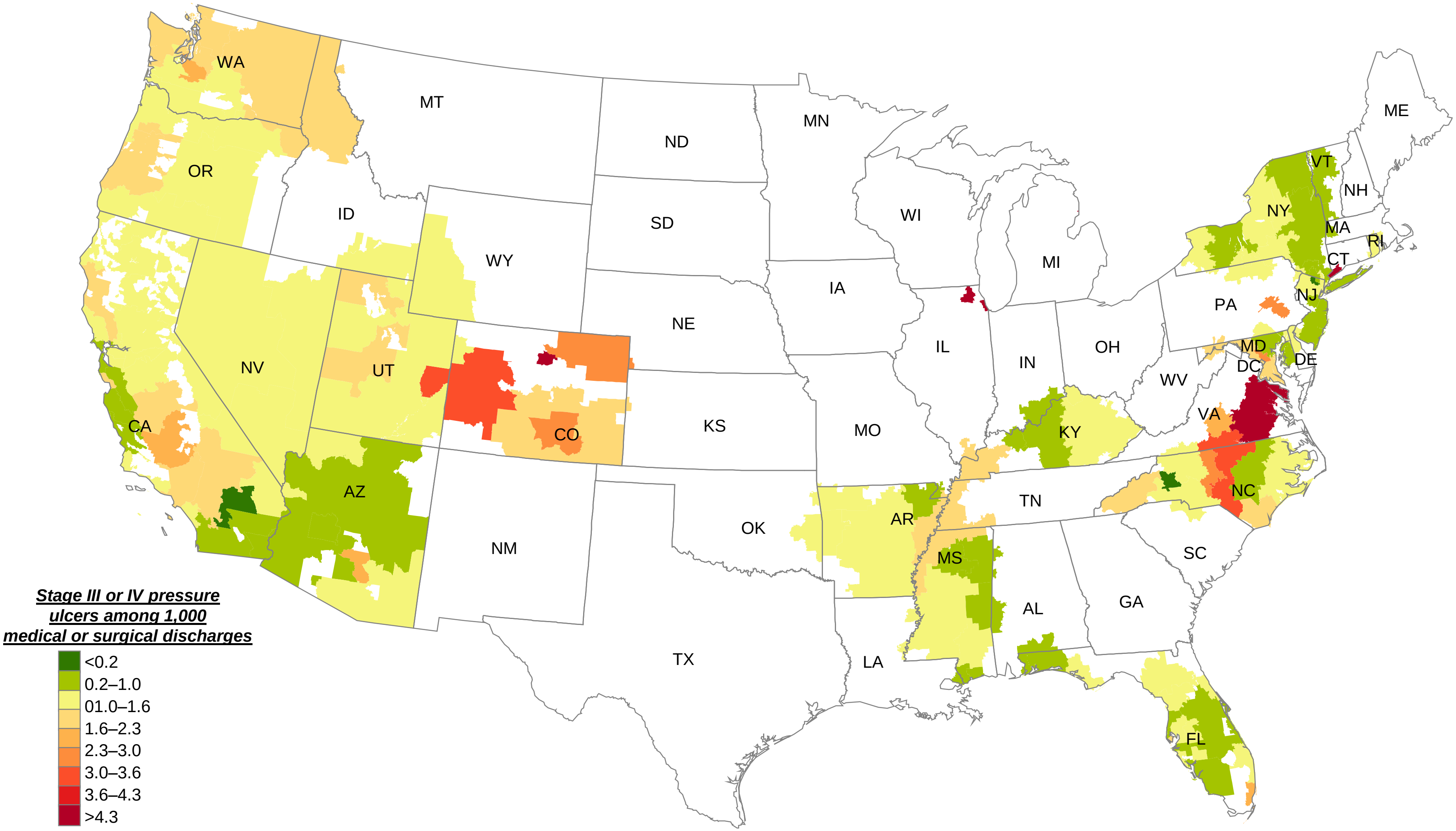

Copyright © 2014 by The Boston Consulting Group, Inc. All rights reserved.

Figure F: State level geographic variability in PSI 03 - Pressure Ulcer Rate

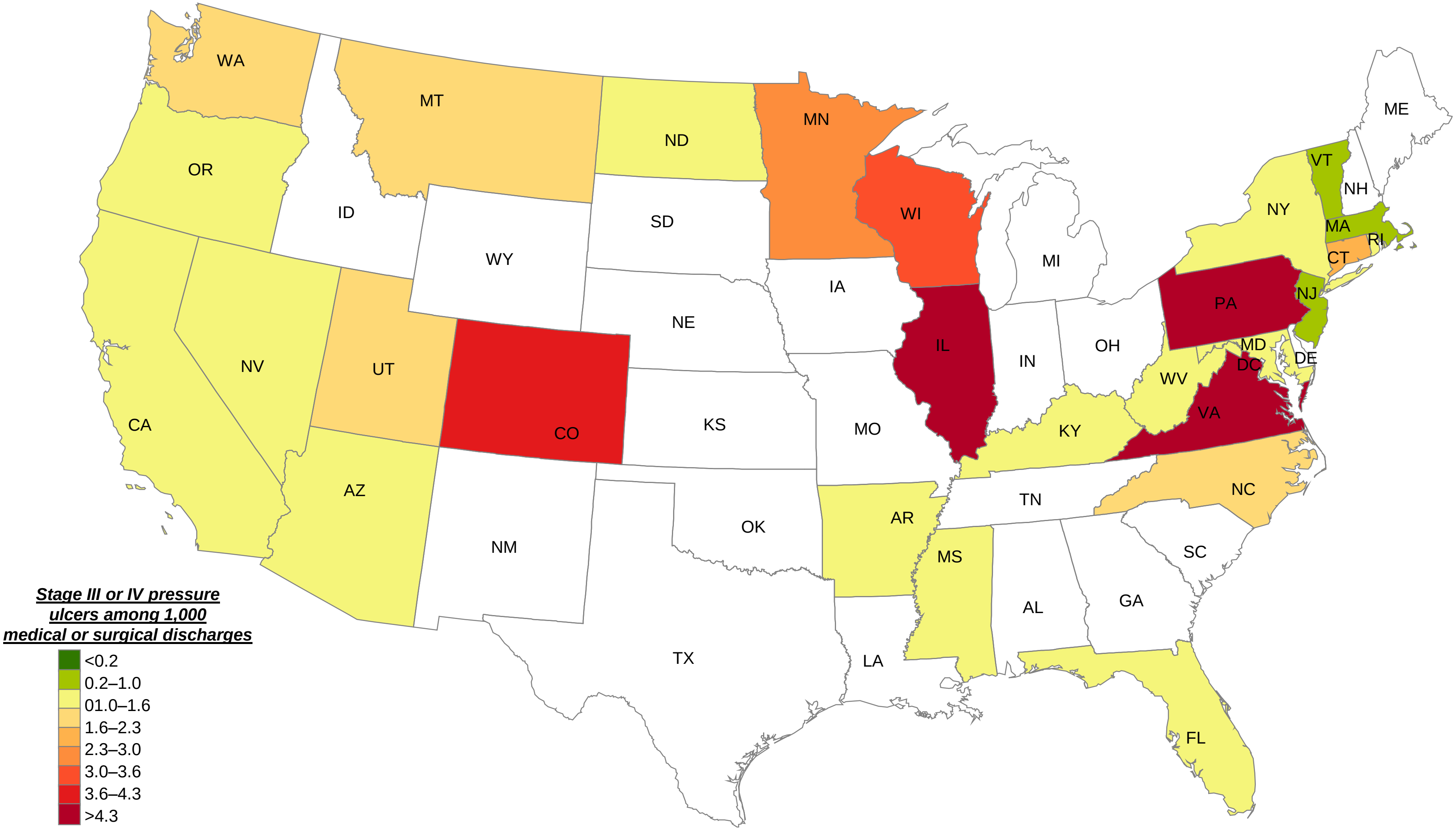

Copyright © 2014 by The Boston Consulting Group, Inc. All rights reserved.

Figure F: HSA level geographic variability in PSI 06 - Iatrogenic Pneumothorax Rate

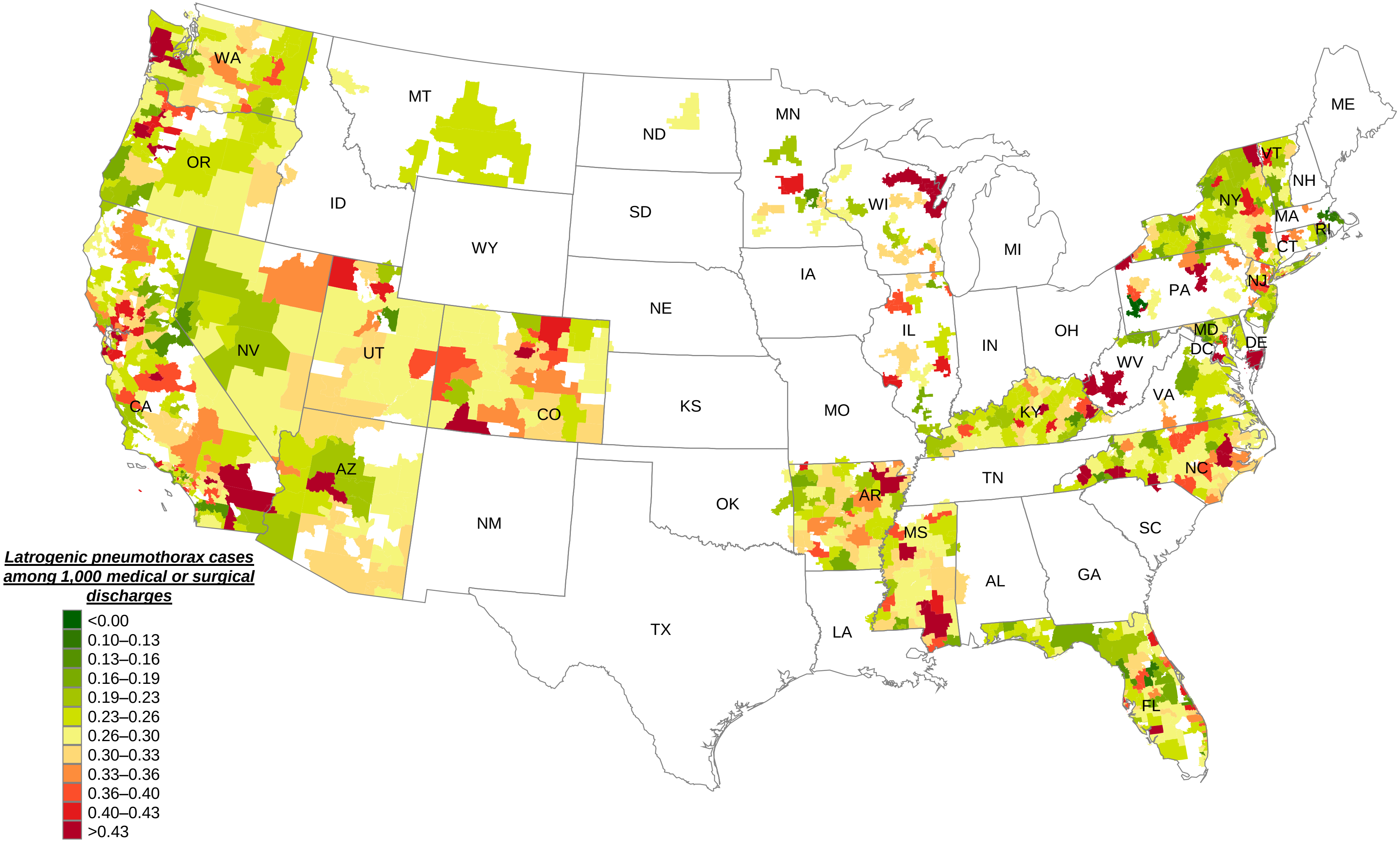

Draft—for discussion only

Copyright © 2014 by The Boston Consulting Group, Inc. All rights reserved.

Figure F: HRR level geographic variability in PSI 06 - Iatrogenic Pneumothorax Rate

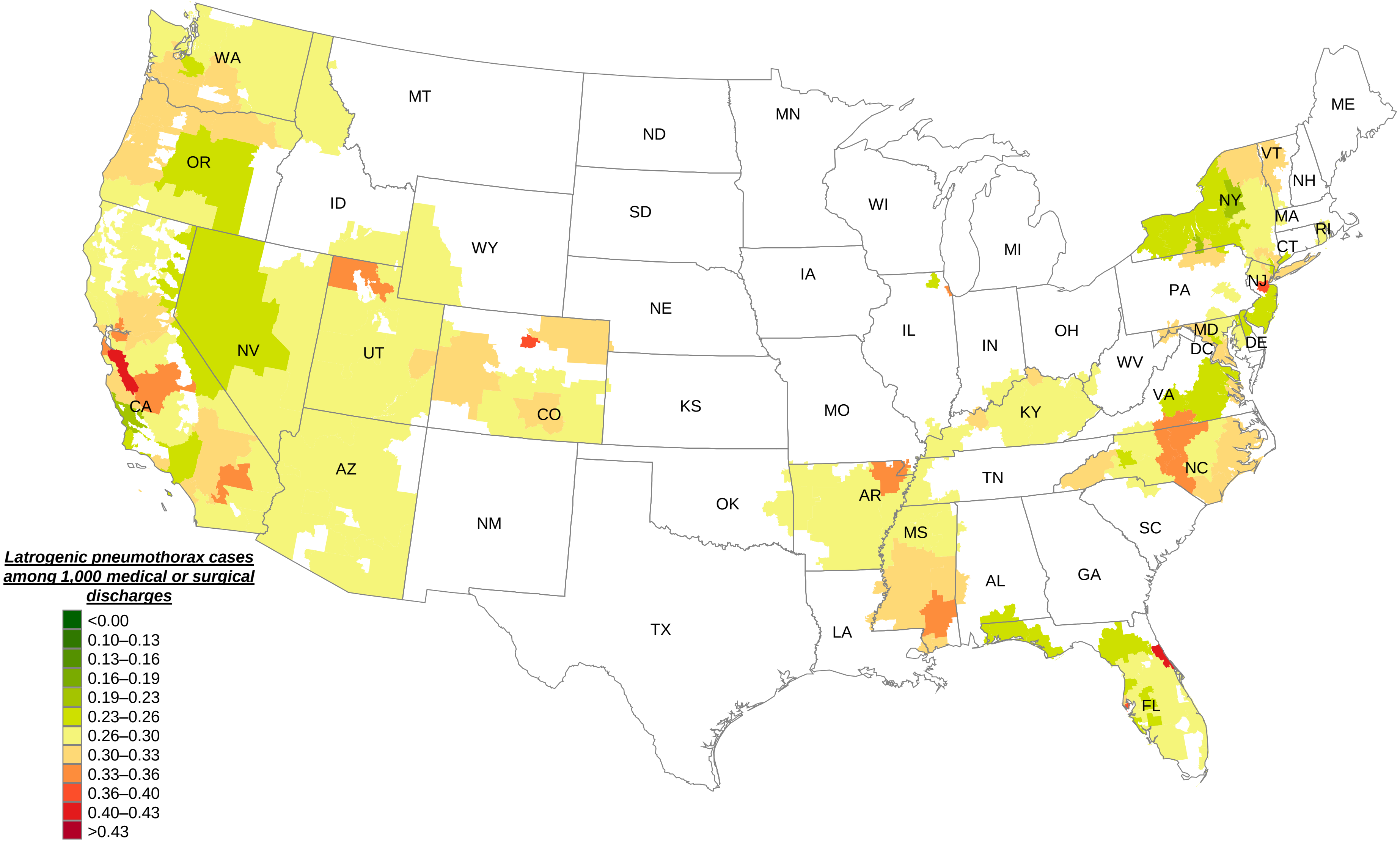

Copyright © 2014 by The Boston Consulting Group, Inc. All rights reserved.

Figure F: State level geographic variability in PSI 06 - Iatrogenic Pneumothorax Rate

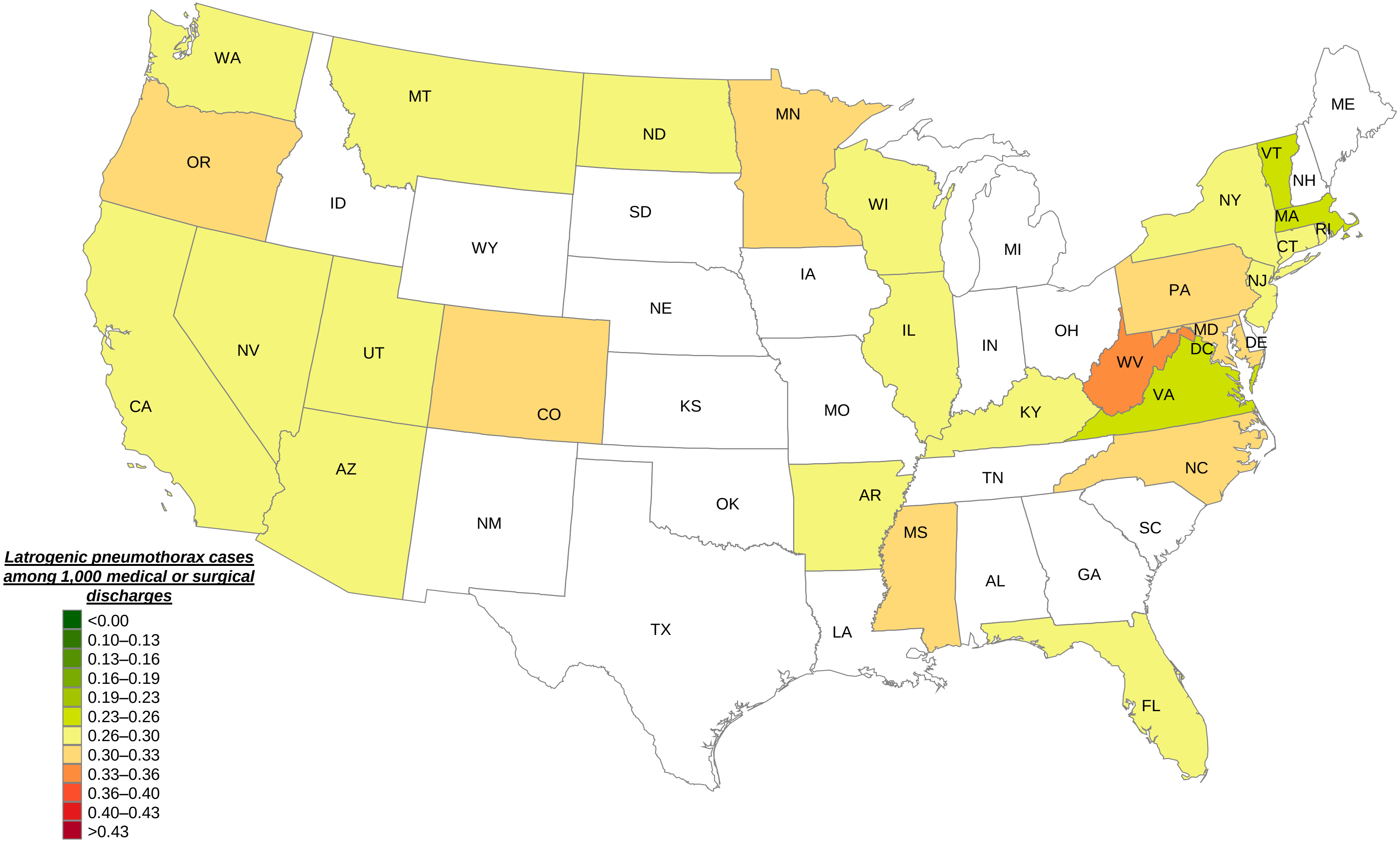

Copyright © 2014 by The Boston Consulting Group, Inc. All rights reserved.

Draft—for discussion only

Figure F: HSA level geographic variability in PSI 07 - Central Venous Catheter Bloodstream Infection Rate

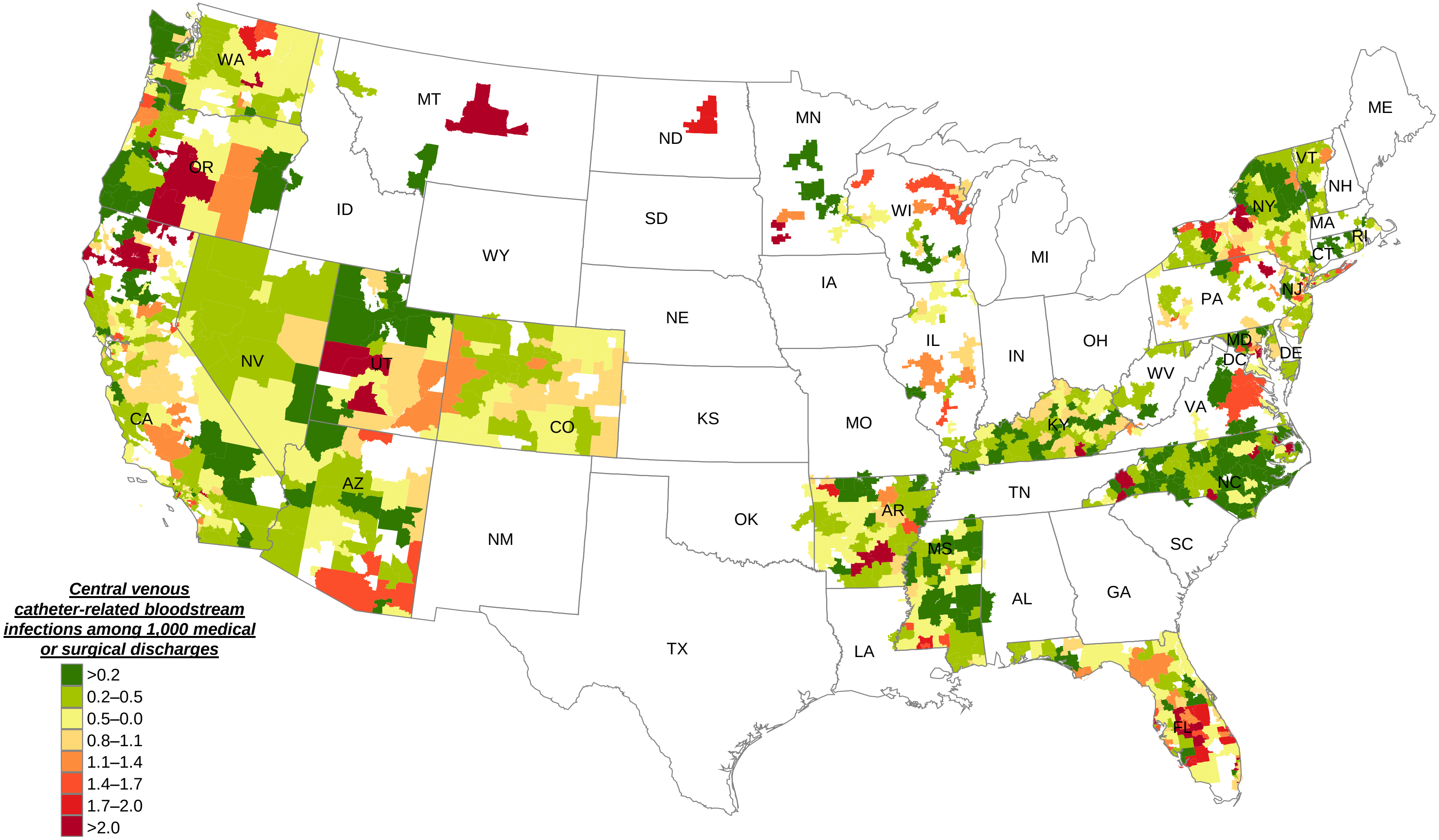

Copyright © 2014 by The Boston Consulting Group, Inc. All rights reserved.

Figure F: HRR level geographic variability in PSI 07 - Central Venous Catheter Bloodstream Infection Rate

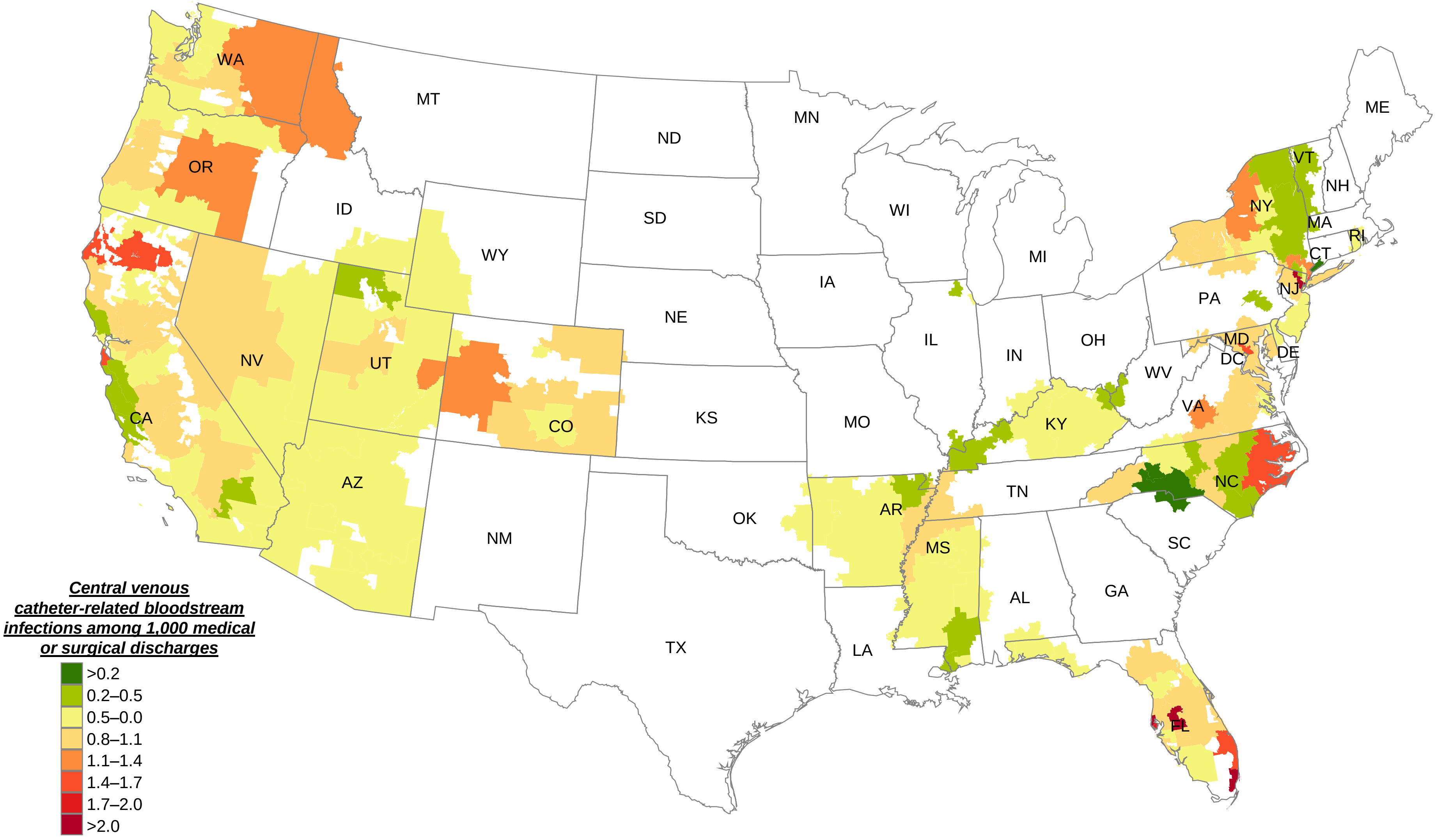

Copyright © 2014 by The Boston Consulting Group, Inc. All rights reserved.

Figure F: State level geographic variability in PSI 07 - Central Venous Catheter Bloodstream Infection Rate

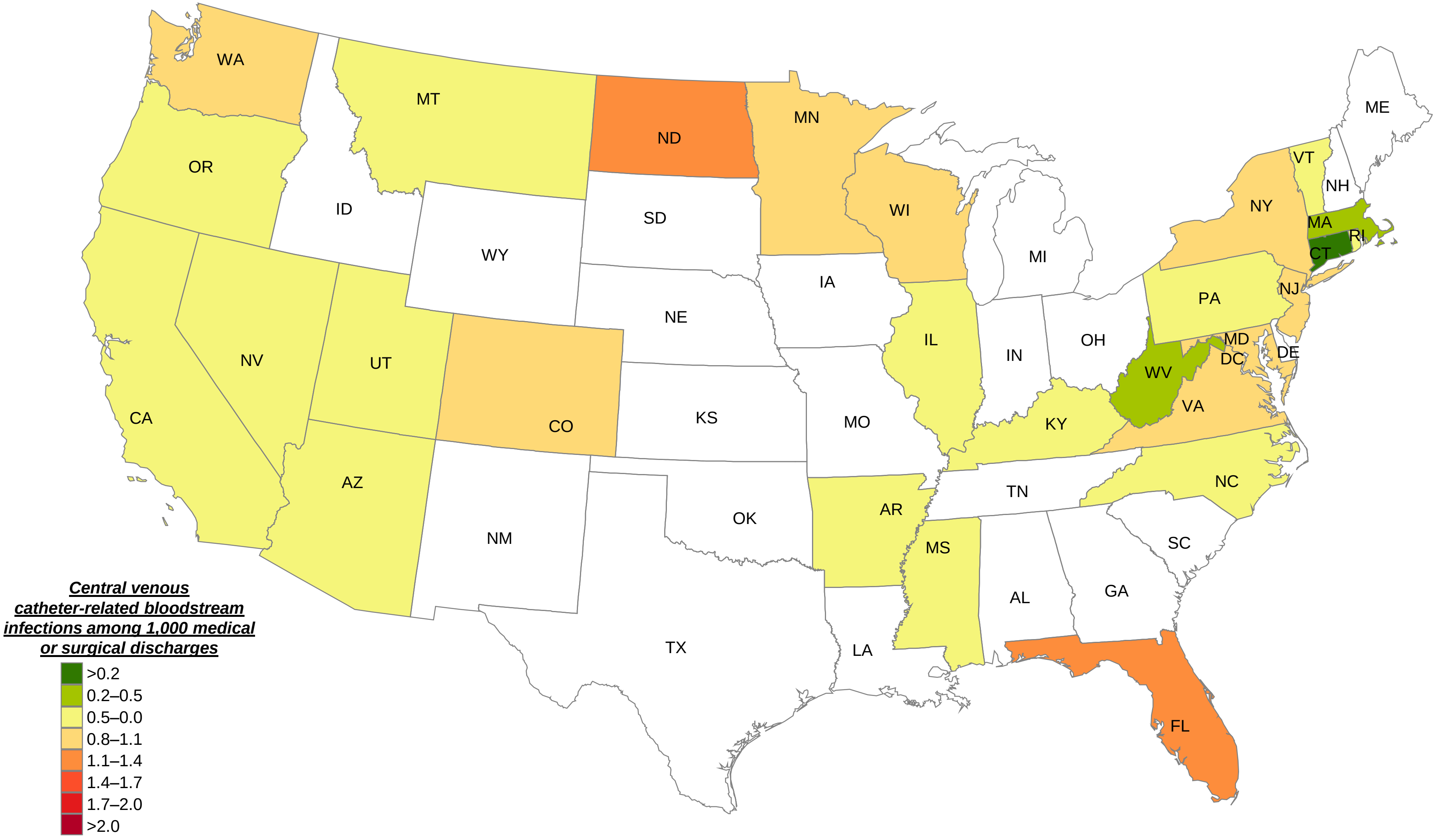

Copyright © 2014 by The Boston Consulting Group, Inc. All rights reserved.

Draft—for discussion only

Figure F: HSA level geographic variability in PSI 08 - Postoperative Hip Fracture Rate

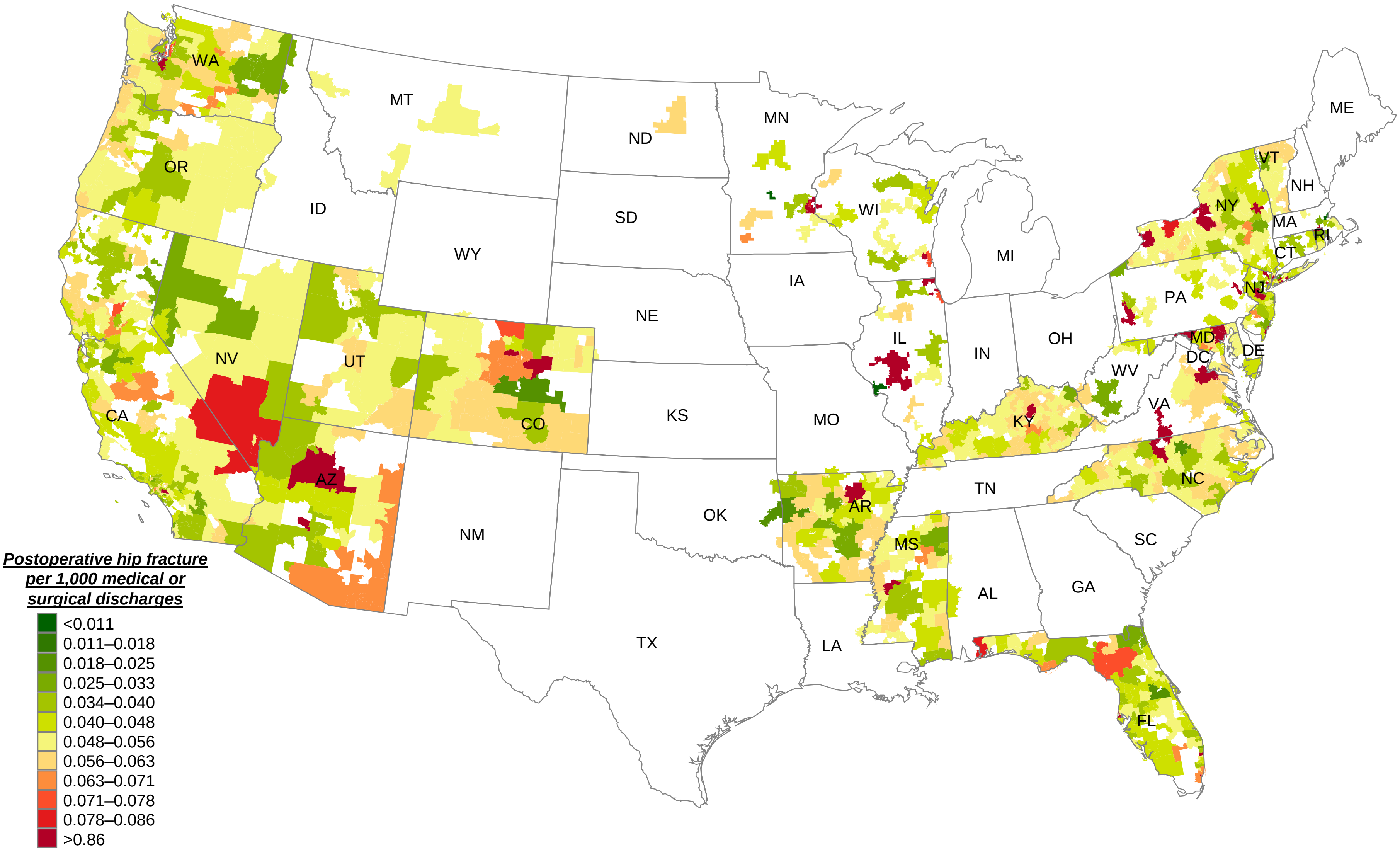

Copyright © 2014 by The Boston Consulting Group, Inc. All rights reserved.

Figure F: HRR level geographic variability in PSI 08 - Postoperative Hip Fracture Rate

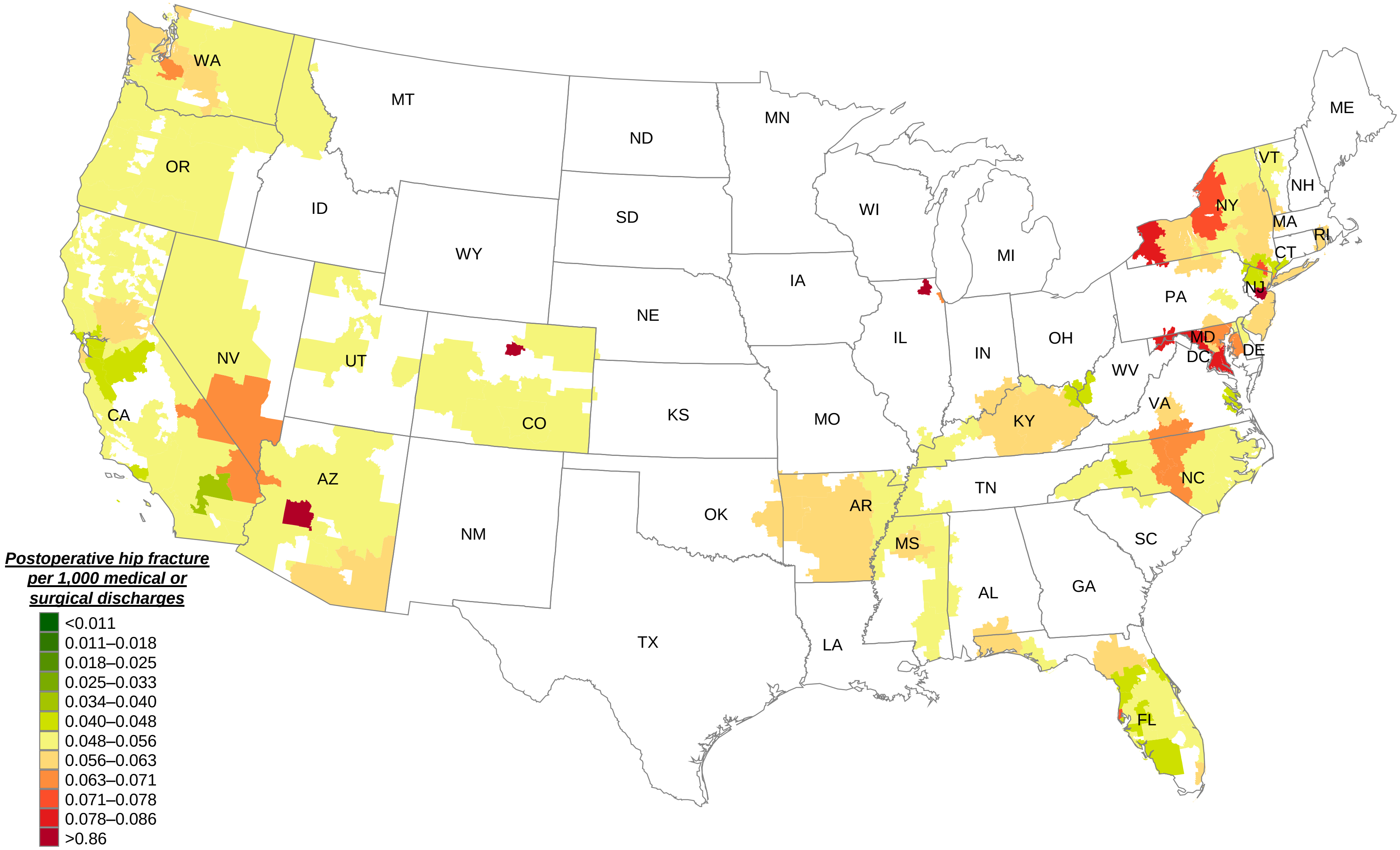

Copyright © 2014 by The Boston Consulting Group, Inc. All rights reserved.

Figure F: State level geographic variability in PSI 08 - Postoperative Hip Fracture Rate

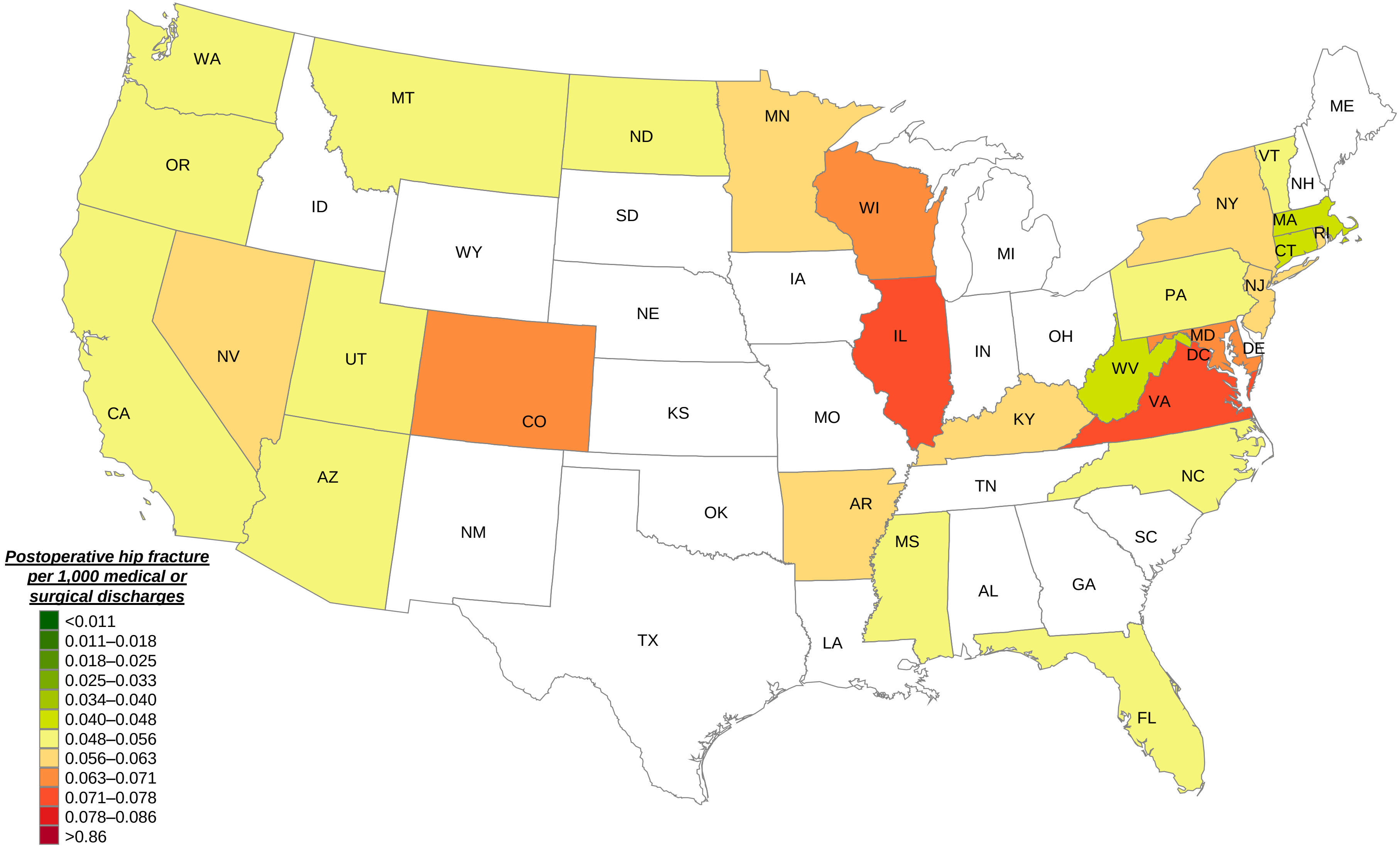

Copyright © 2014 by The Boston Consulting Group, Inc. All rights reserved.

Figure F: HSA level geographic variability in PSI12 - Postop. Pulmonary Embolism or DVT Rate

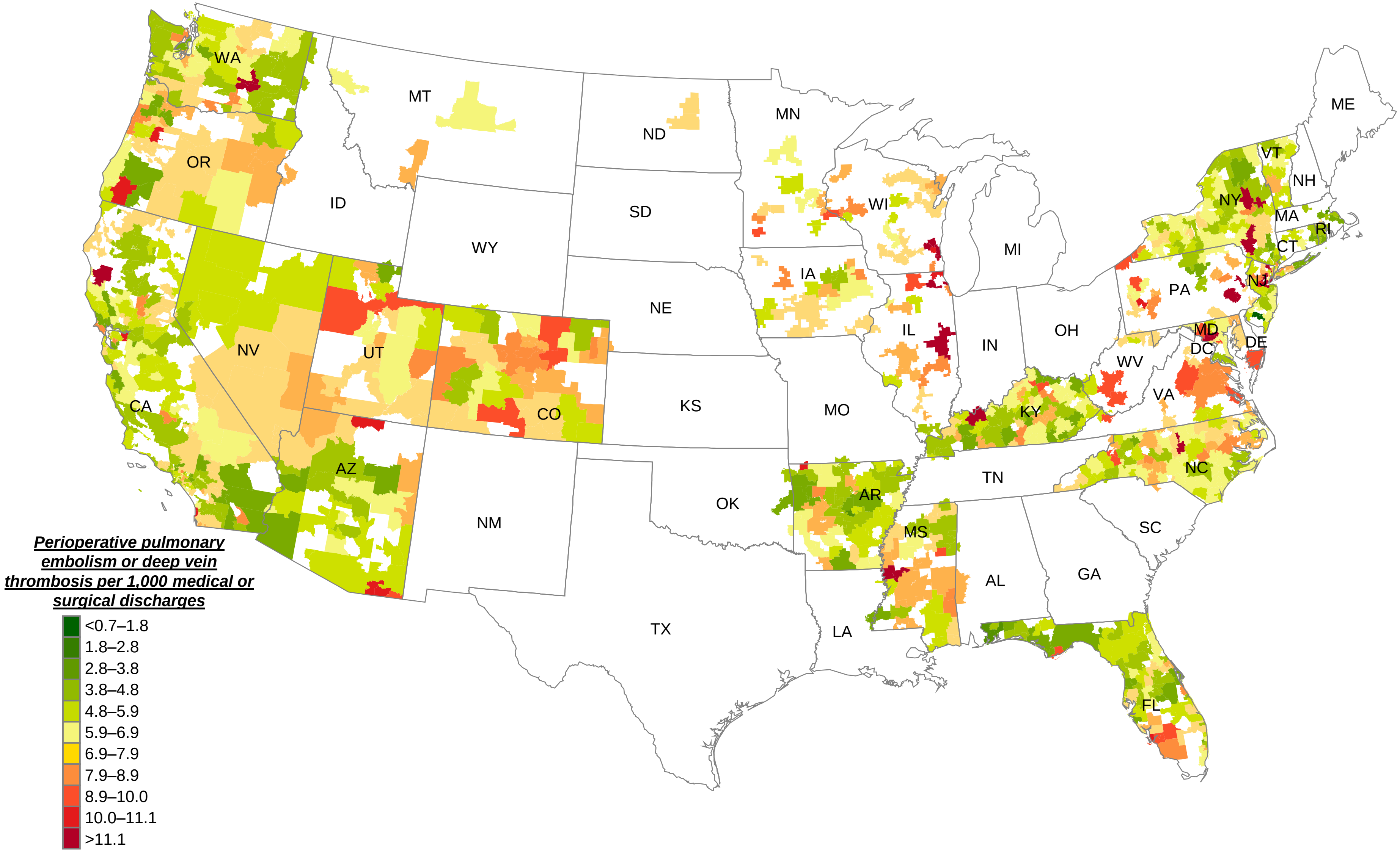

Copyright © 2014 by The Boston Consulting Group, Inc. All rights reserved.

Figure F: HRR level geographic variability in PSI12 - Postop. Pulmonary Embolism or DVT Rate

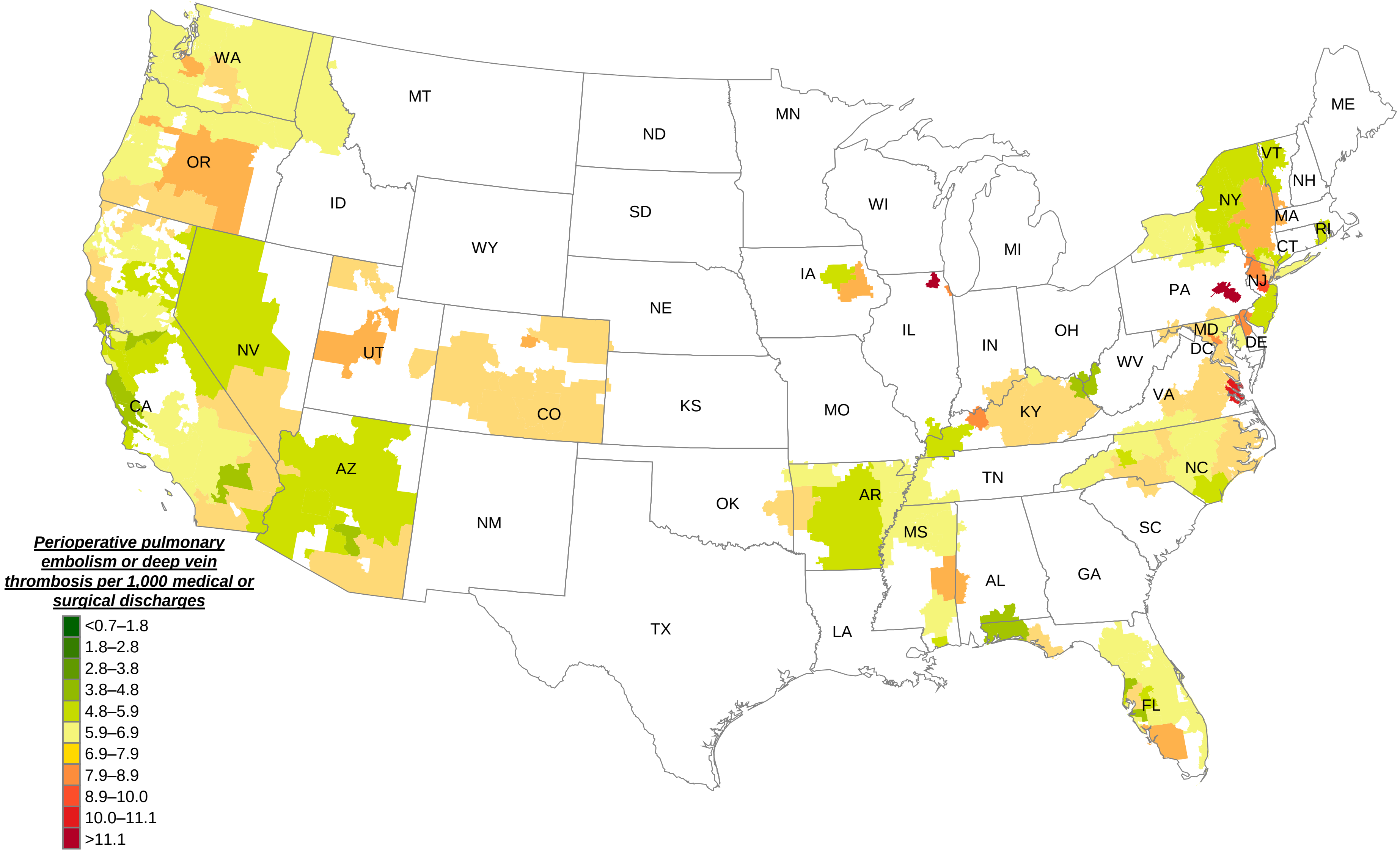

Copyright © 2014 by The Boston Consulting Group, Inc. All rights reserved.

Figure F: State level geographic variability in PSI12 - Postop. Pulmonary Embolism or DVT Rate

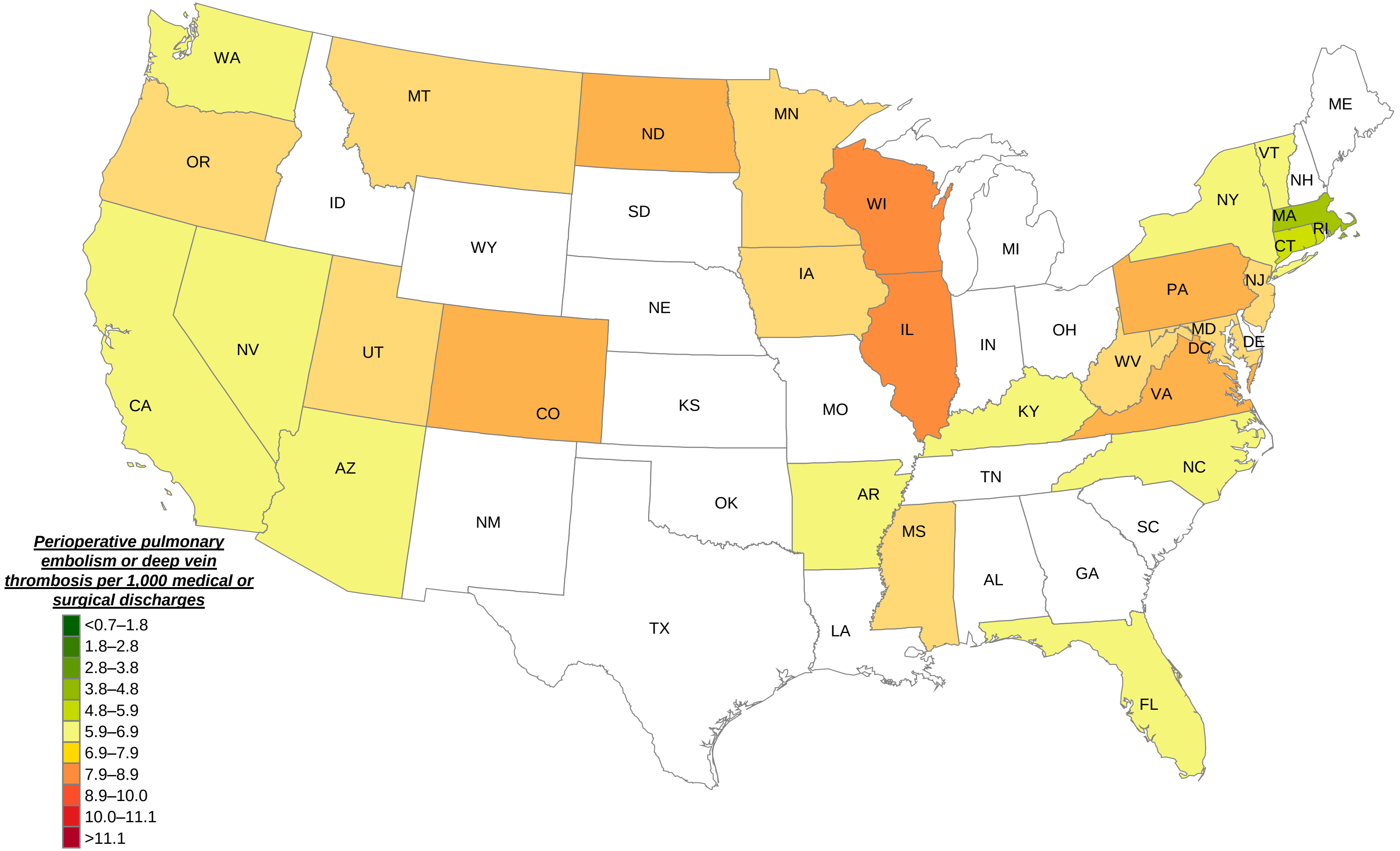

Copyright © 2014 by The Boston Consulting Group, Inc. All rights reserved.

Figure F: HSA level geographic variability in PSI13 - Postoperative Sepsis Rate

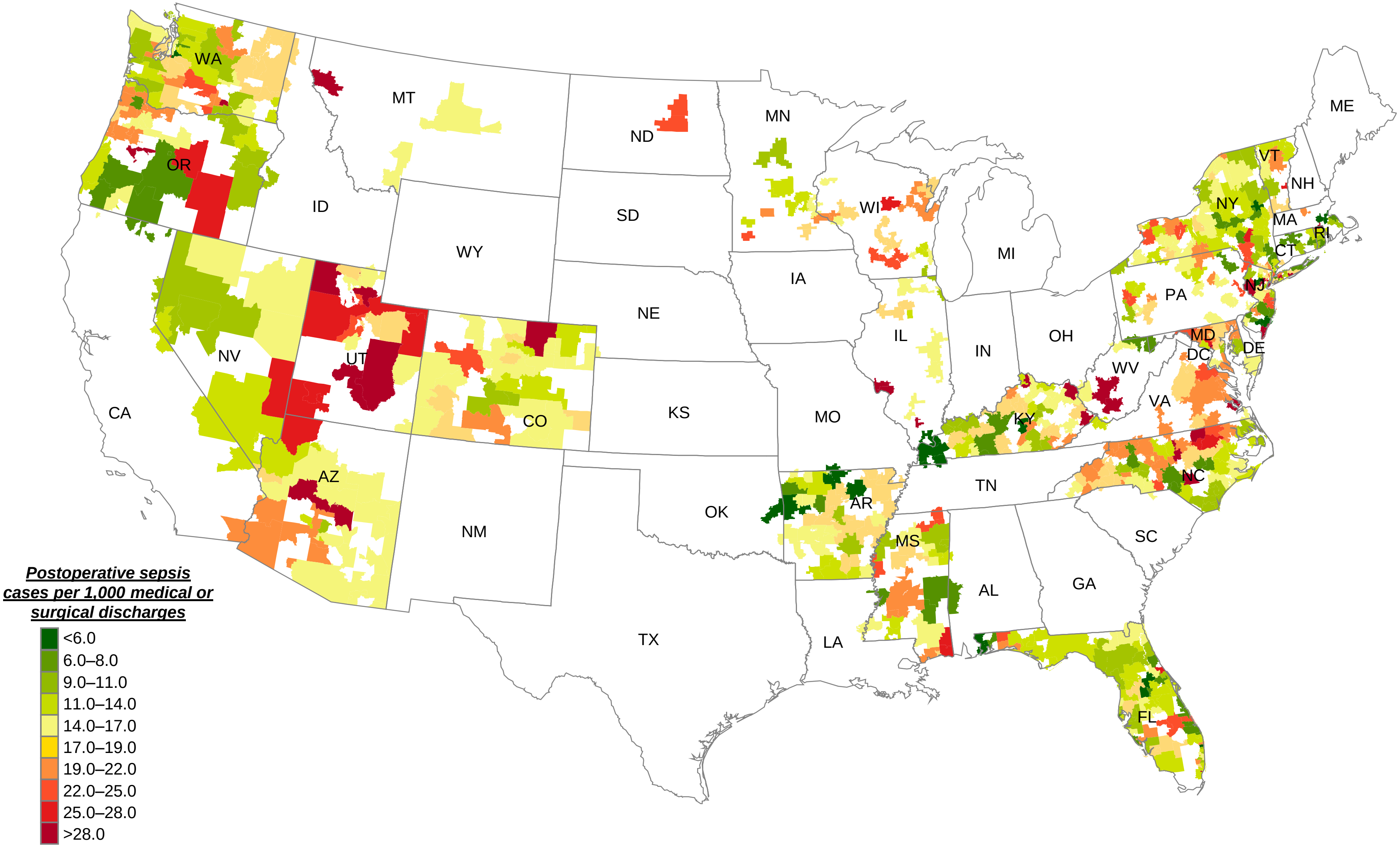

Copyright © 2014 by The Boston Consulting Group, Inc. All rights reserved.

Draft—for discussion only

Figure F: HRR level geographic variability in PSI13 - Postoperative Sepsis Rate

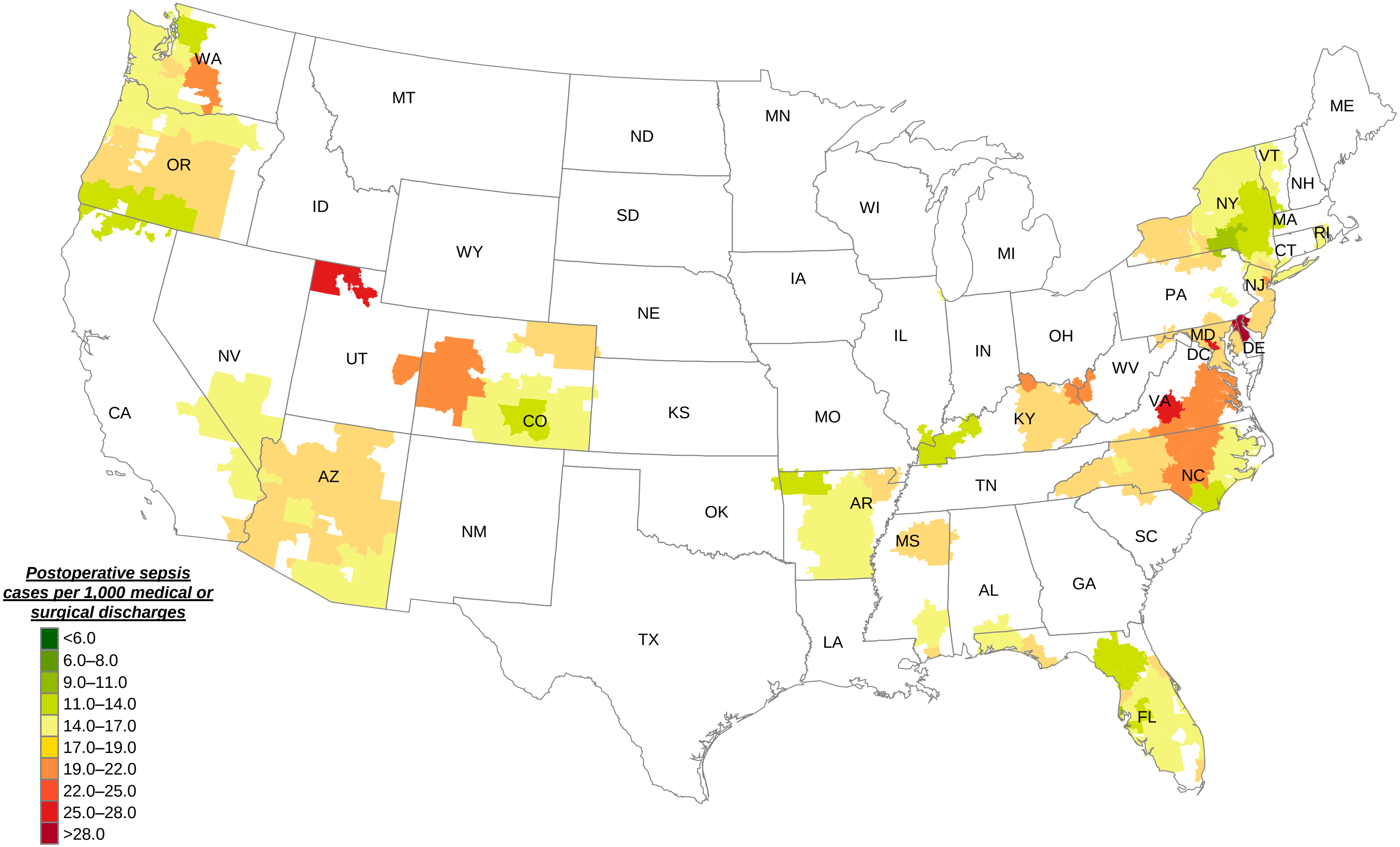

Copyright © 2014 by The Boston Consulting Group, Inc. All rights reserved.

Draft—for discussion only

Figure F: State level geographic variability in PSI13 - Postoperative Sepsis Rate

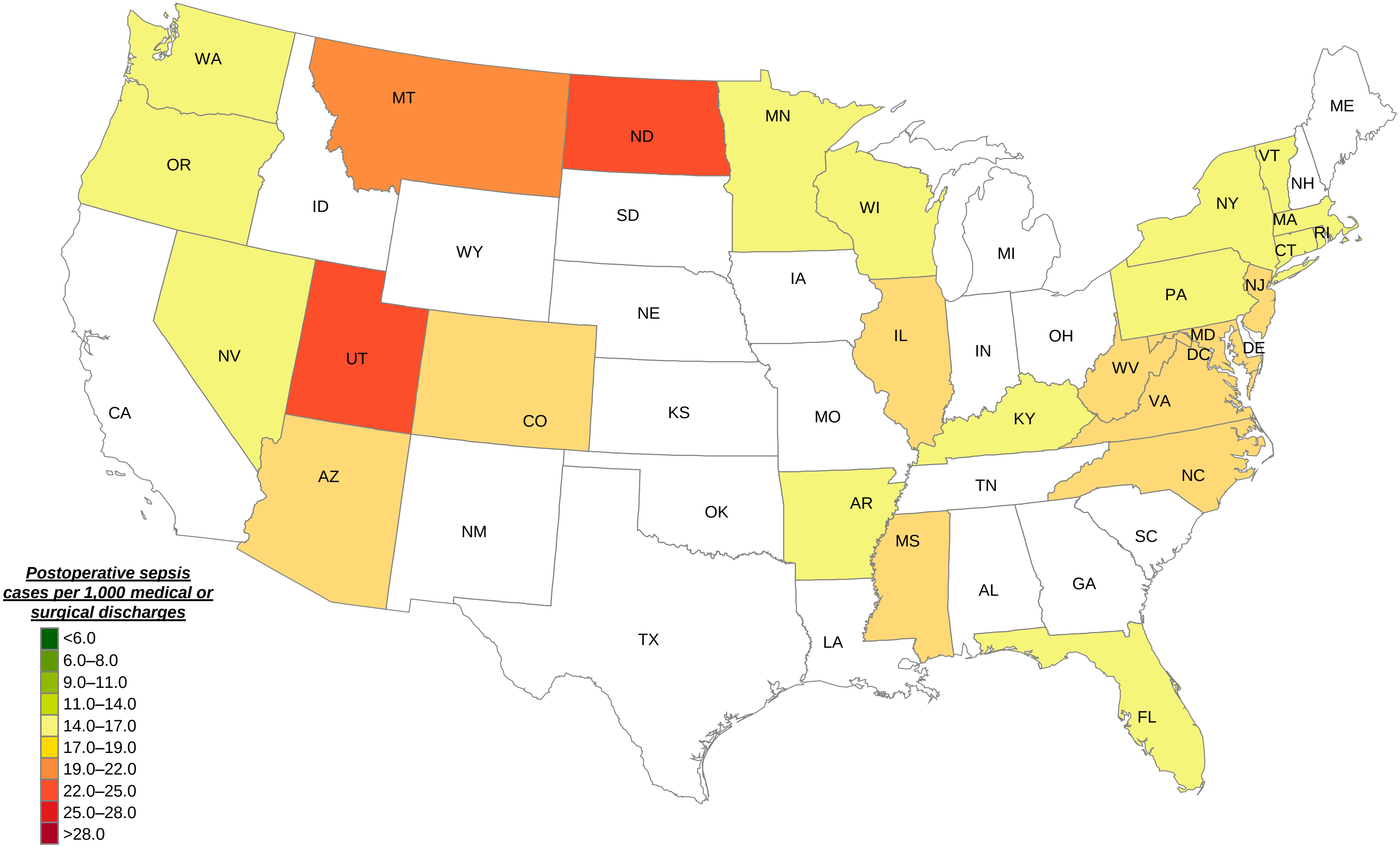

Copyright © 2014 by The Boston Consulting Group, Inc. All rights reserved.

Figure F: HSA level geographic variability in PSI14 - Postoperative wound dehiscence rate

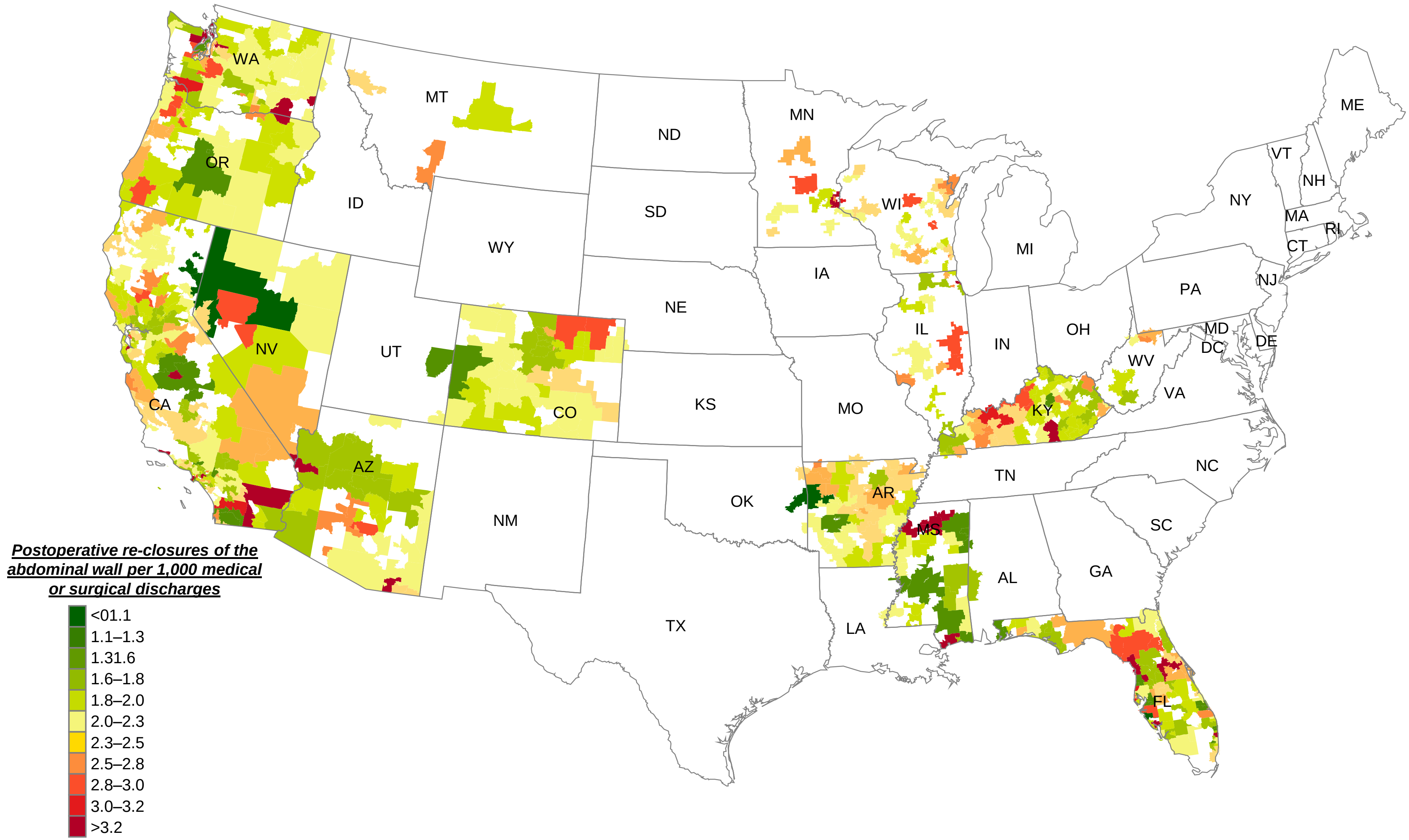

Copyright © 2014 by The Boston Consulting Group, Inc. All rights reserved.

Draft—for discussion only

Figure F: HRR level geographic variability in PSI14 - Postoperative wound dehiscence rate

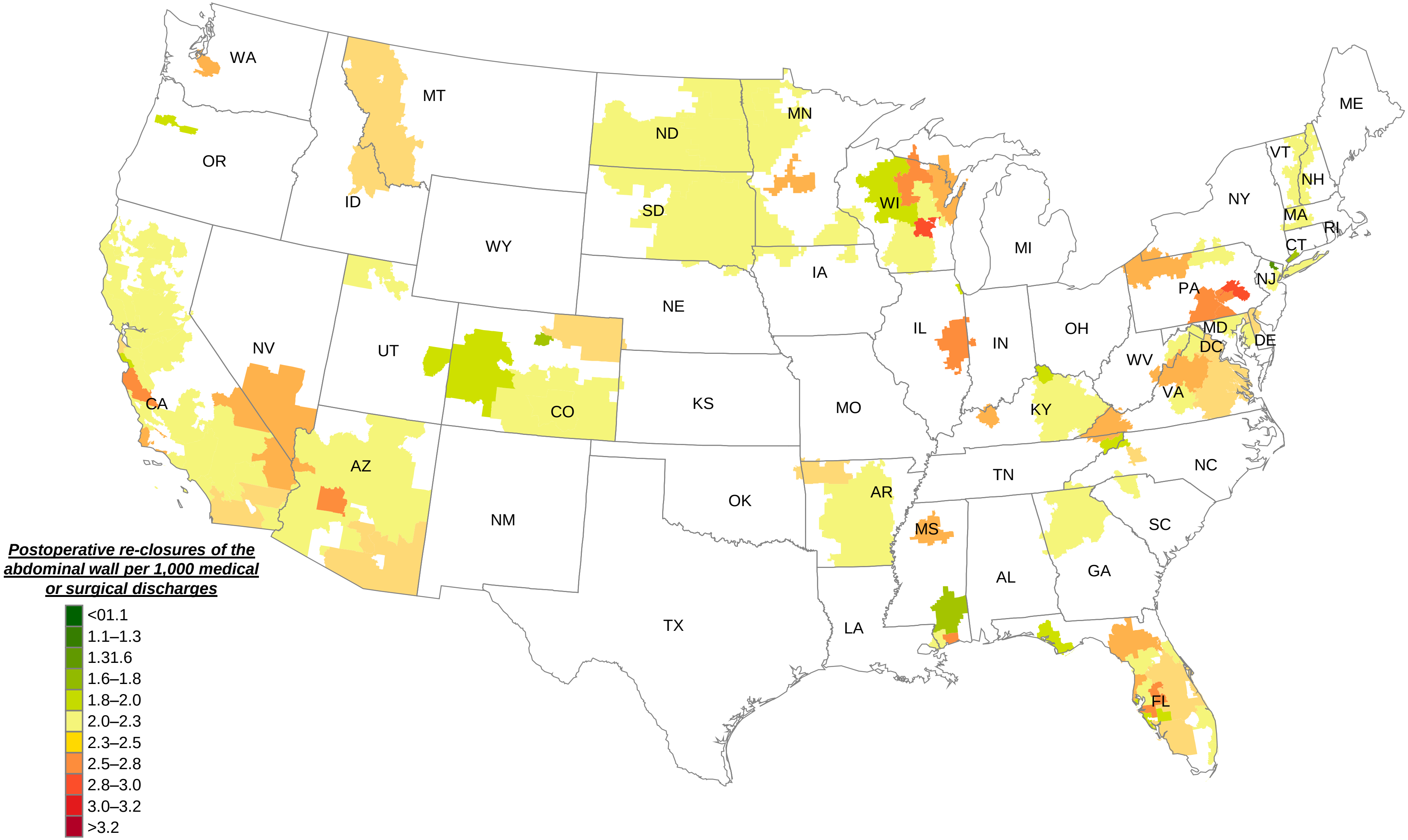

Copyright © 2014 by The Boston Consulting Group, Inc. All rights reserved.

Figure F: State level geographic variability in PSI14 - Postoperative wound dehiscence rate

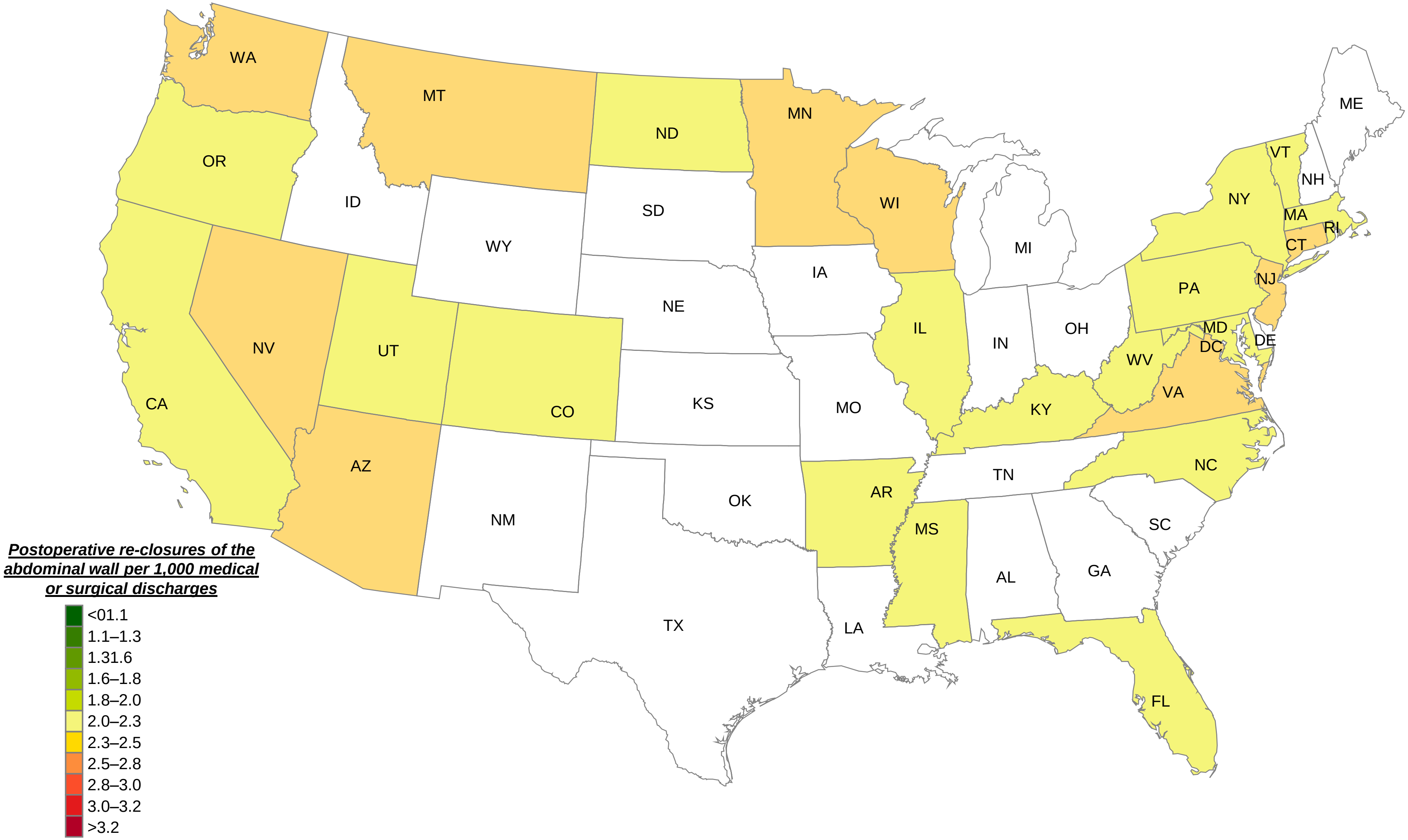

Copyright © 2014 by The Boston Consulting Group, Inc. All rights reserved.

Figure F: HSA level geographic variability in PSI15 - Accidental puncture or laceration rate

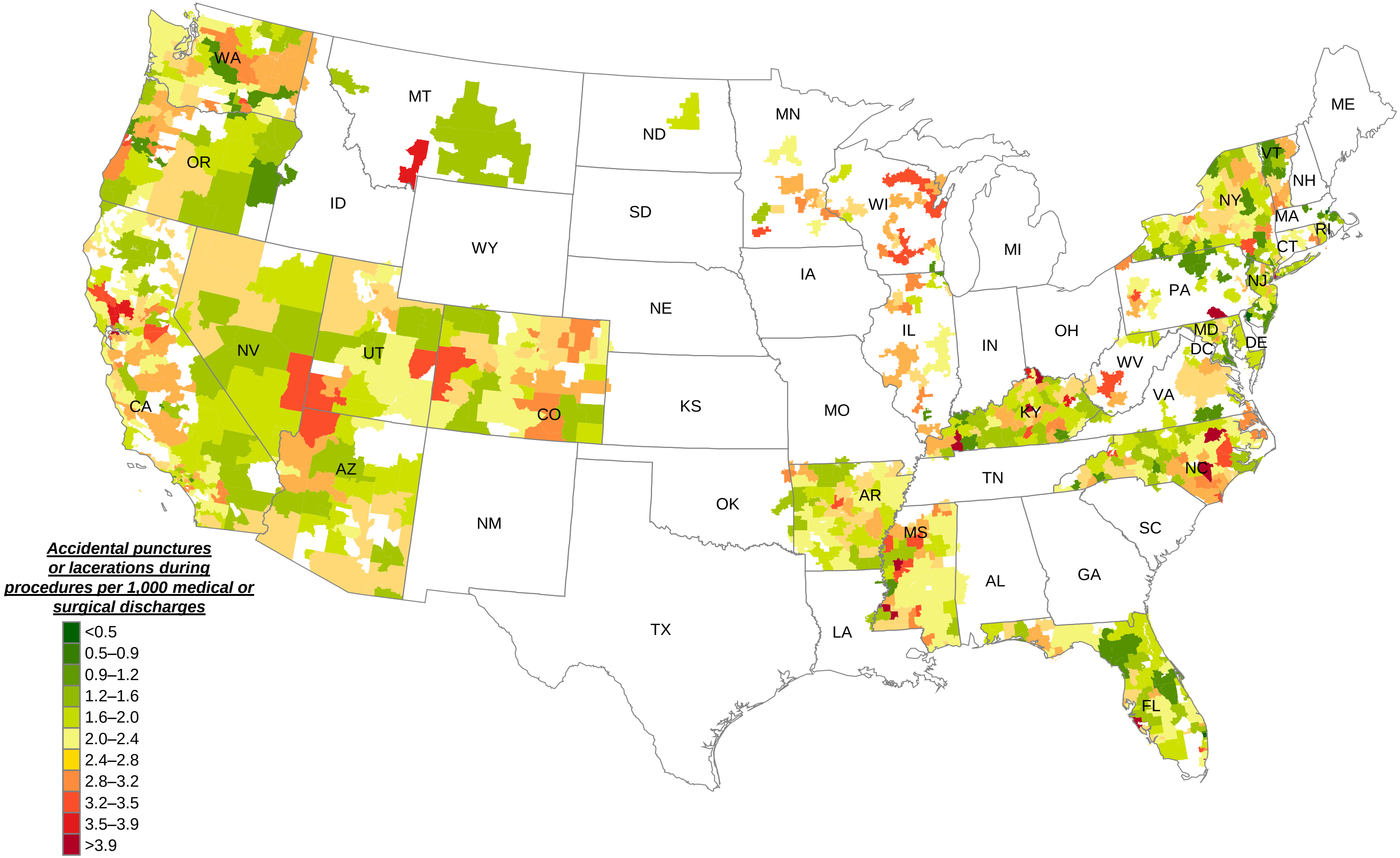

Copyright © 2014 by The Boston Consulting Group, Inc. All rights reserved.

Figure F: HRR level geographic variability in PSI15 - Accidental puncture or laceration rate

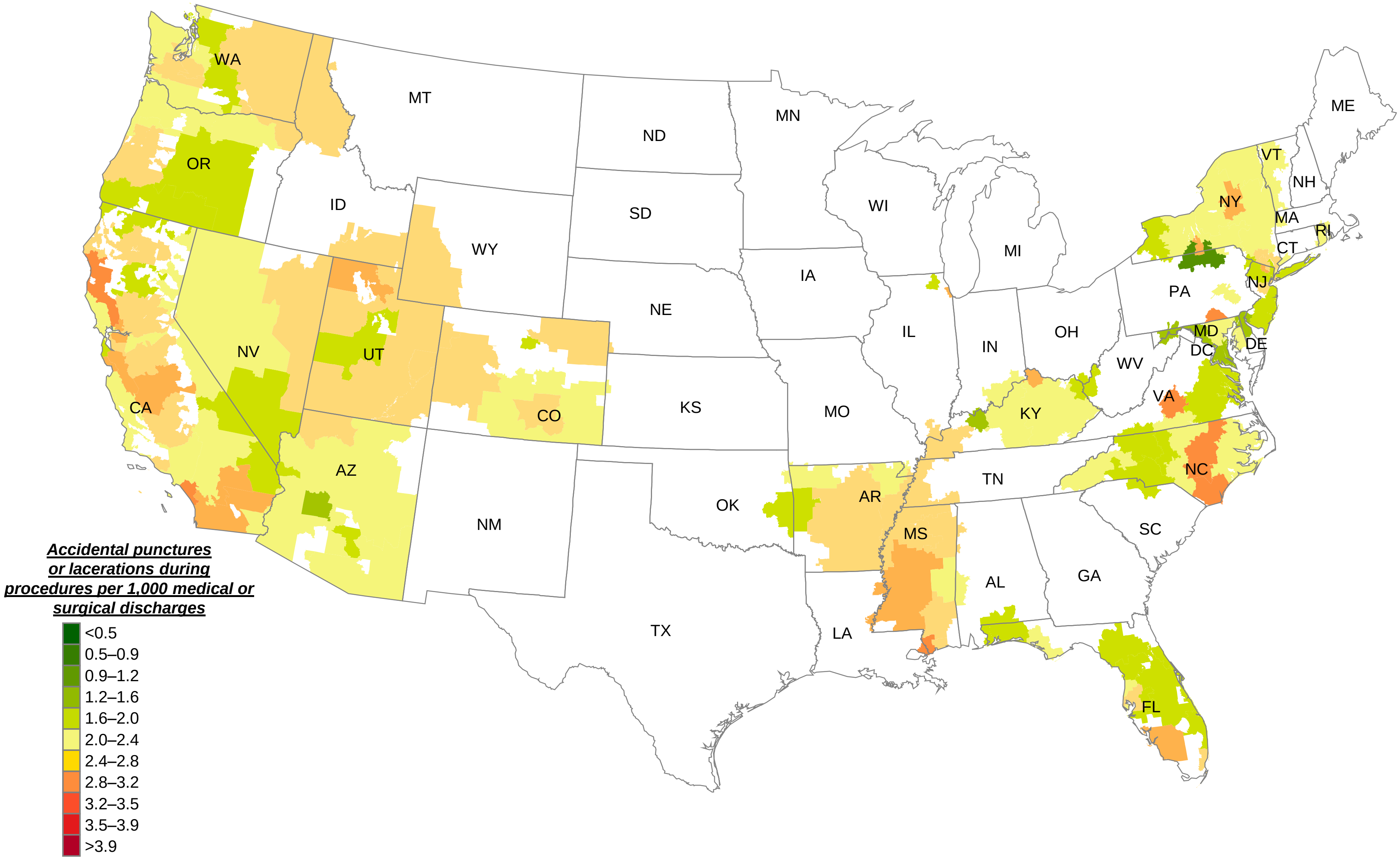

Copyright © 2014 by The Boston Consulting Group, Inc. All rights reserved.

Figure F: State level geographic variability in PSI15 - Accidental puncture or laceration rate

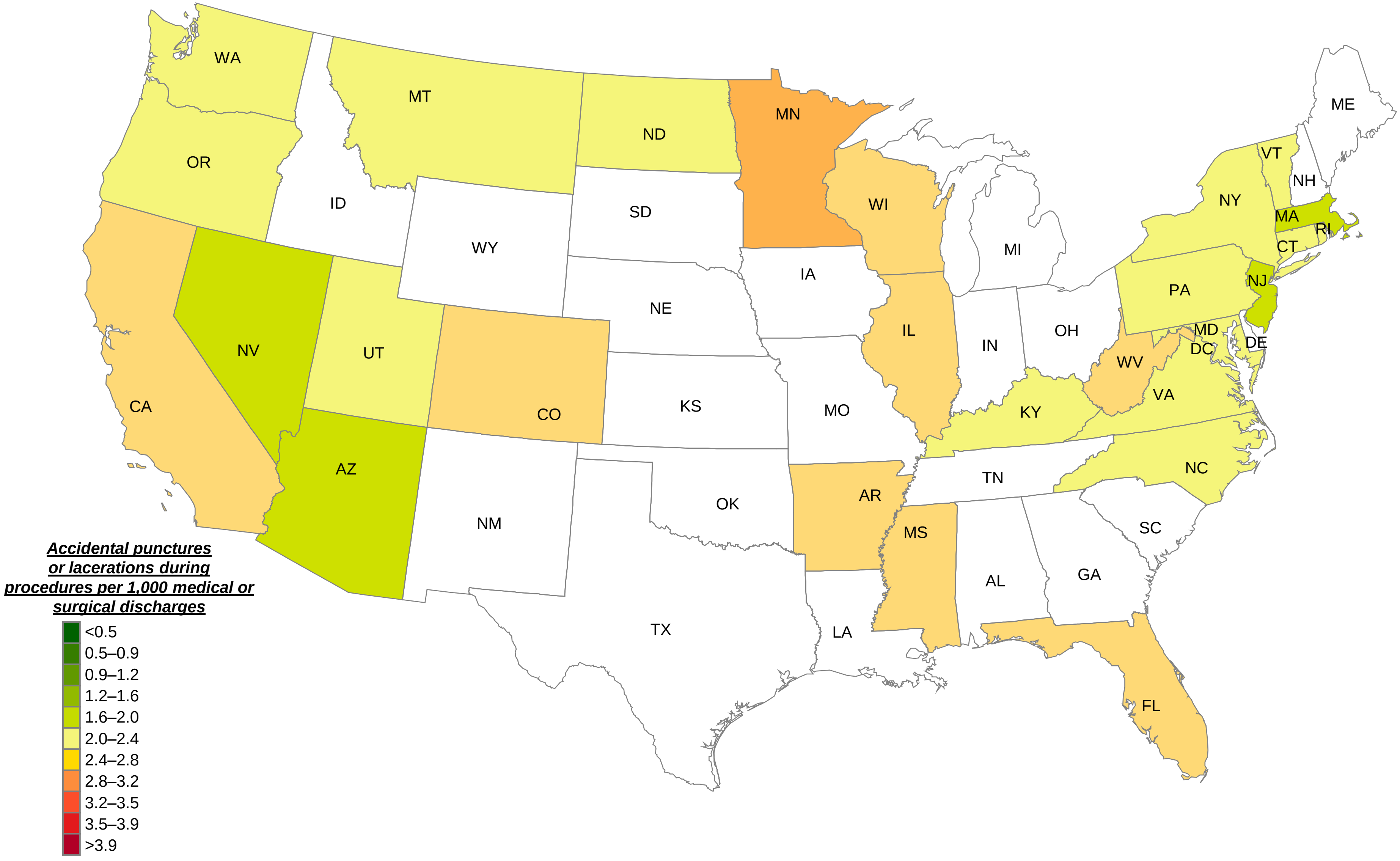

Copyright © 2014 by The Boston Consulting Group, Inc. All rights reserved.

Draft—for discussion only

Figure F: County level geographic variability in PQI01 - Diabetes short-term complications admission rate

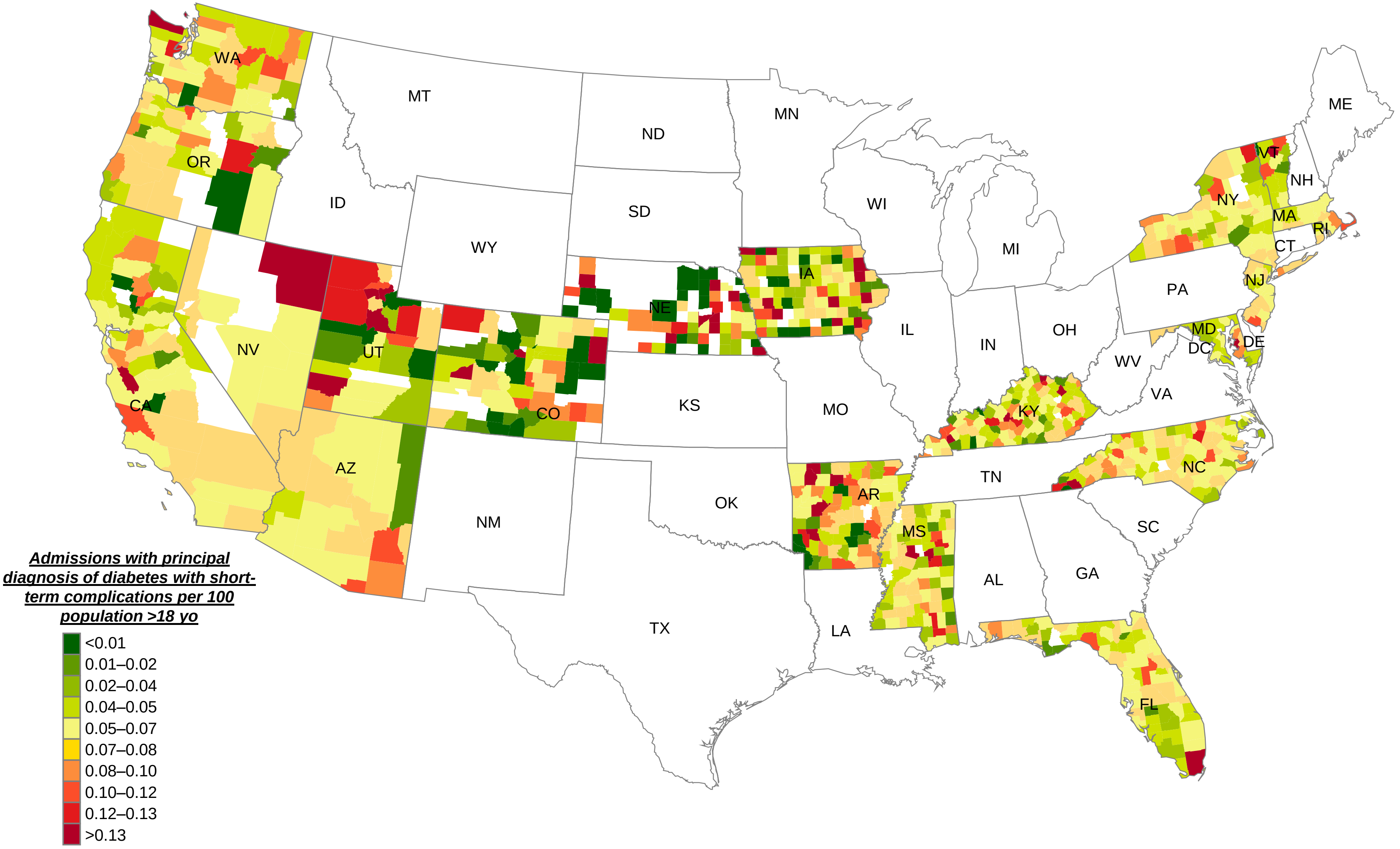

Copyright © 2014 by The Boston Consulting Group, Inc. All rights reserved.

Figure F: State level geographic variability in PQI01 - Diabetes short-term complications admission rate

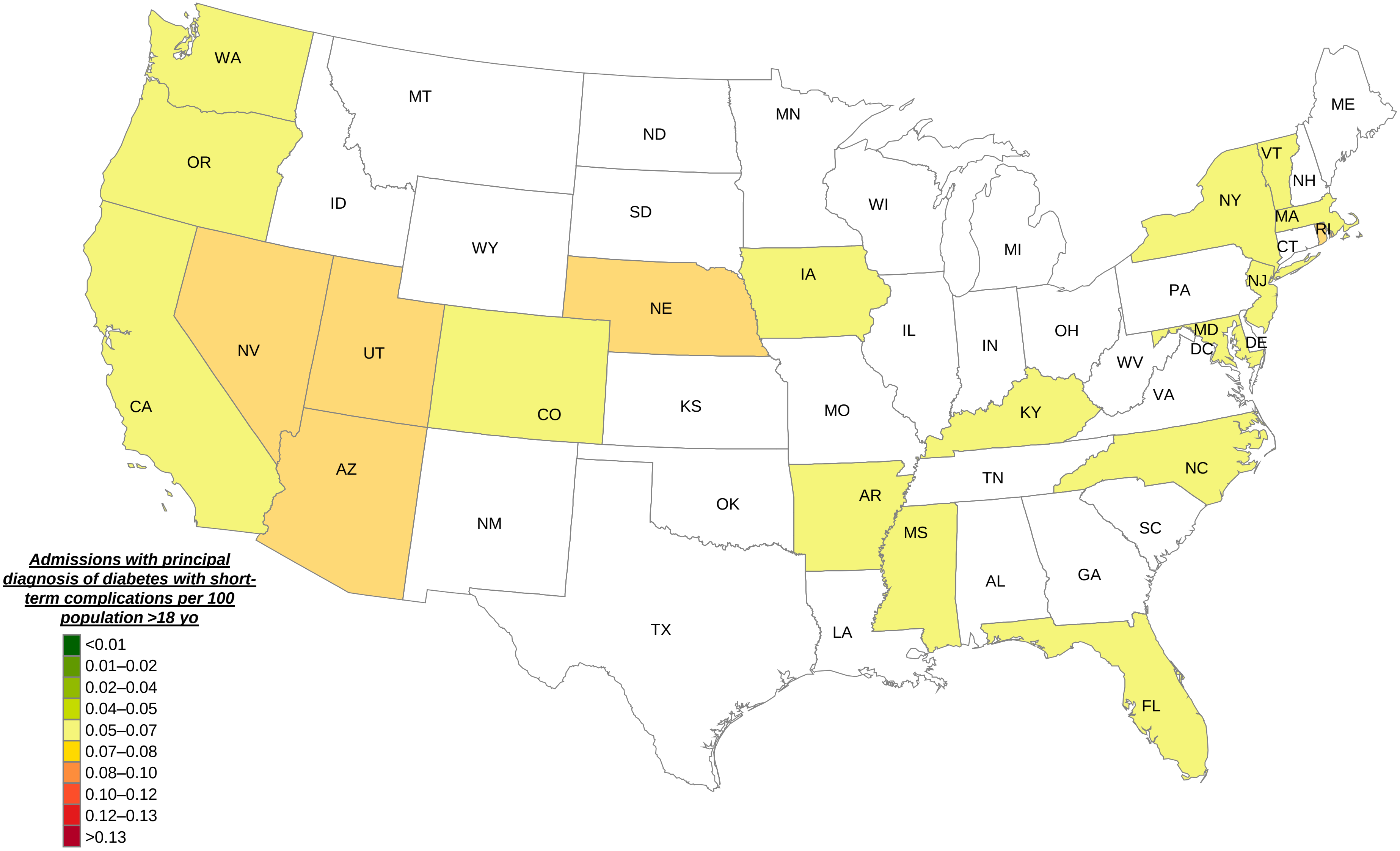

Copyright © 2014 by The Boston Consulting Group, Inc. All rights reserved.

Draft—for discussion only

Figure F: County level geographic variability in PQI03 - Diabetes long-term complications admission rate

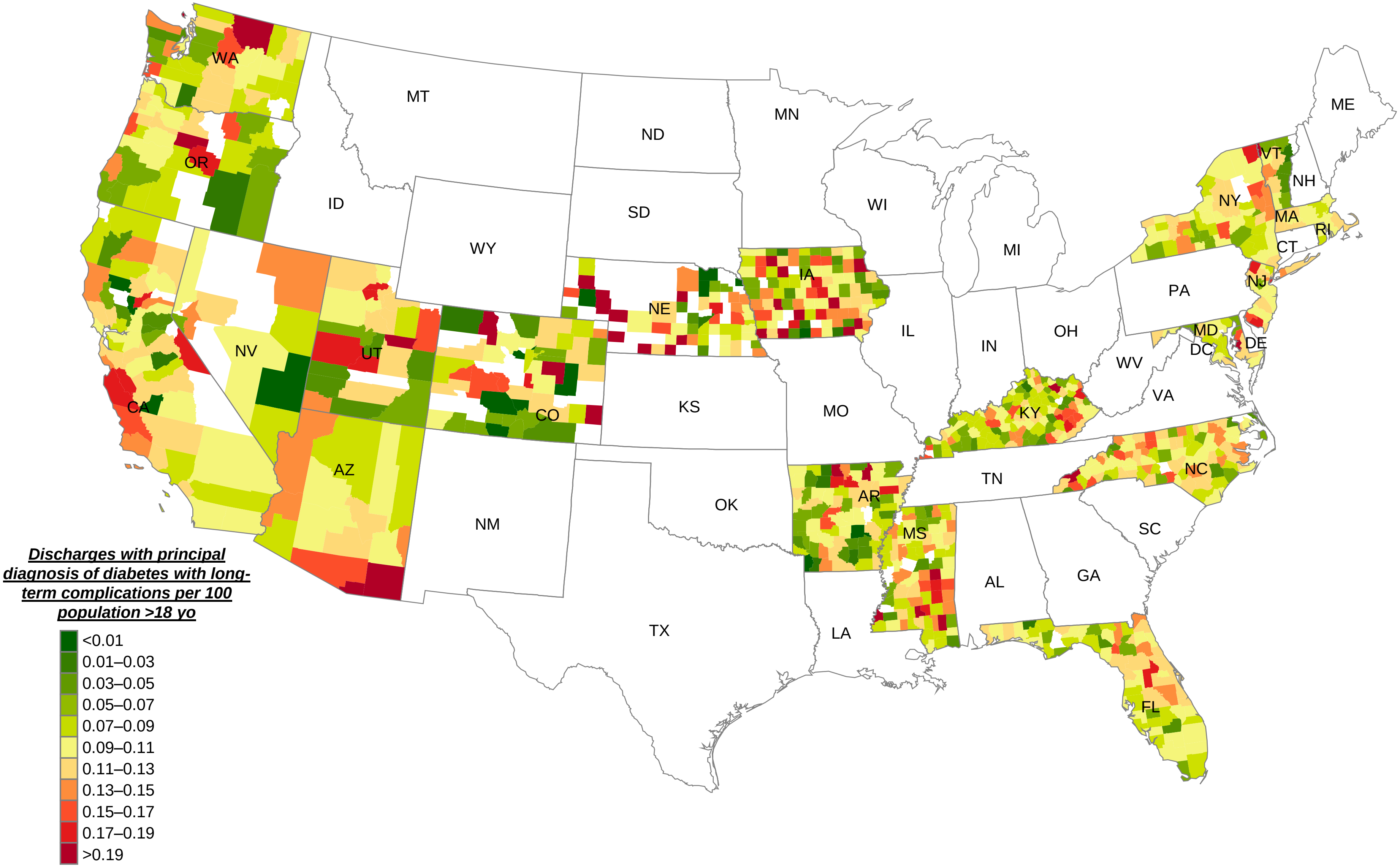

Copyright © 2014 by The Boston Consulting Group, Inc. All rights reserved.

Figure F: State level geographic variability in PQI03 - Diabetes long-term complications admission rate

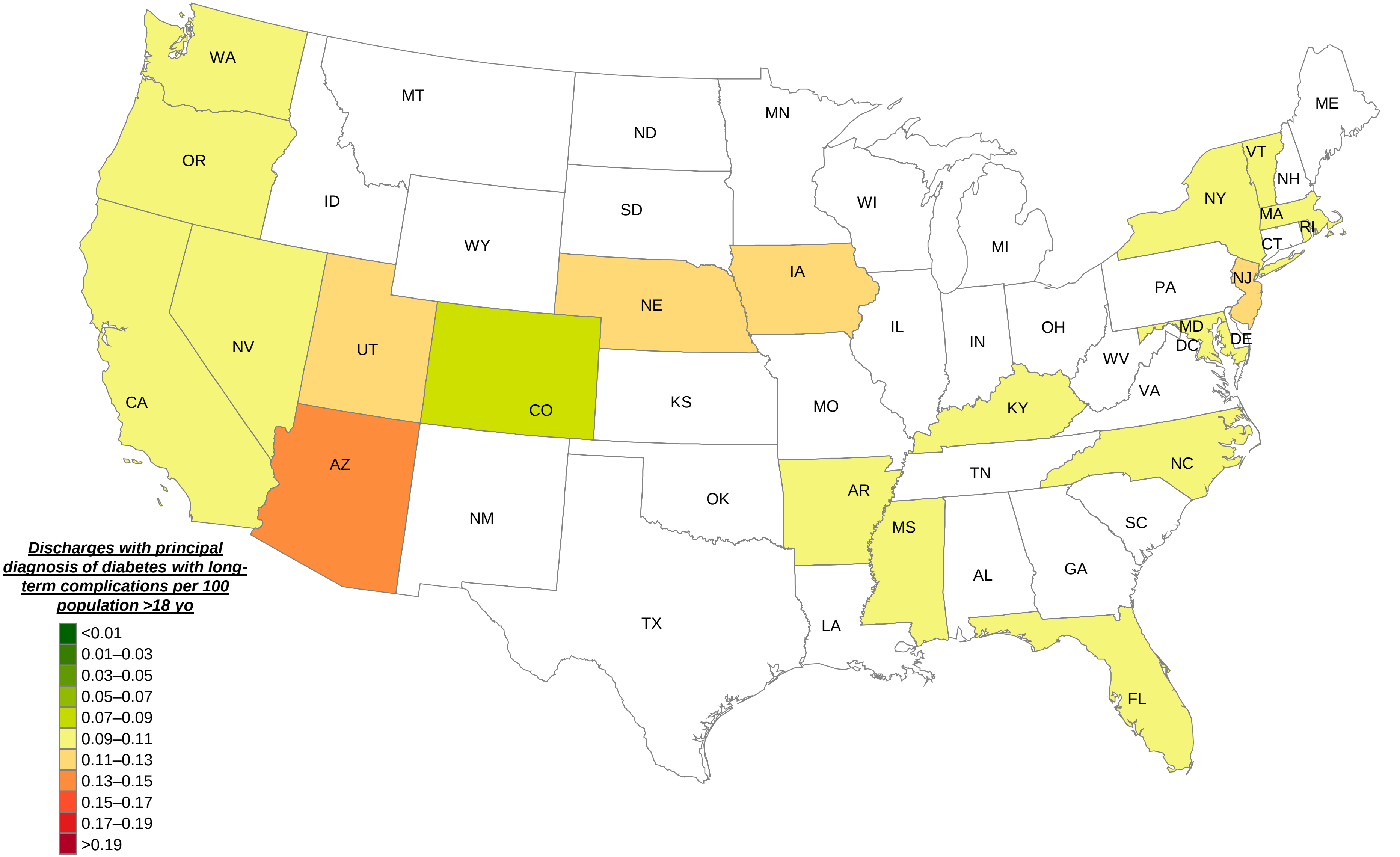

Copyright © 2014 by The Boston Consulting Group, Inc. All rights reserved.

Figure F: County level geographic variability in PQI05 - COPD or asthma in older adults admission rate

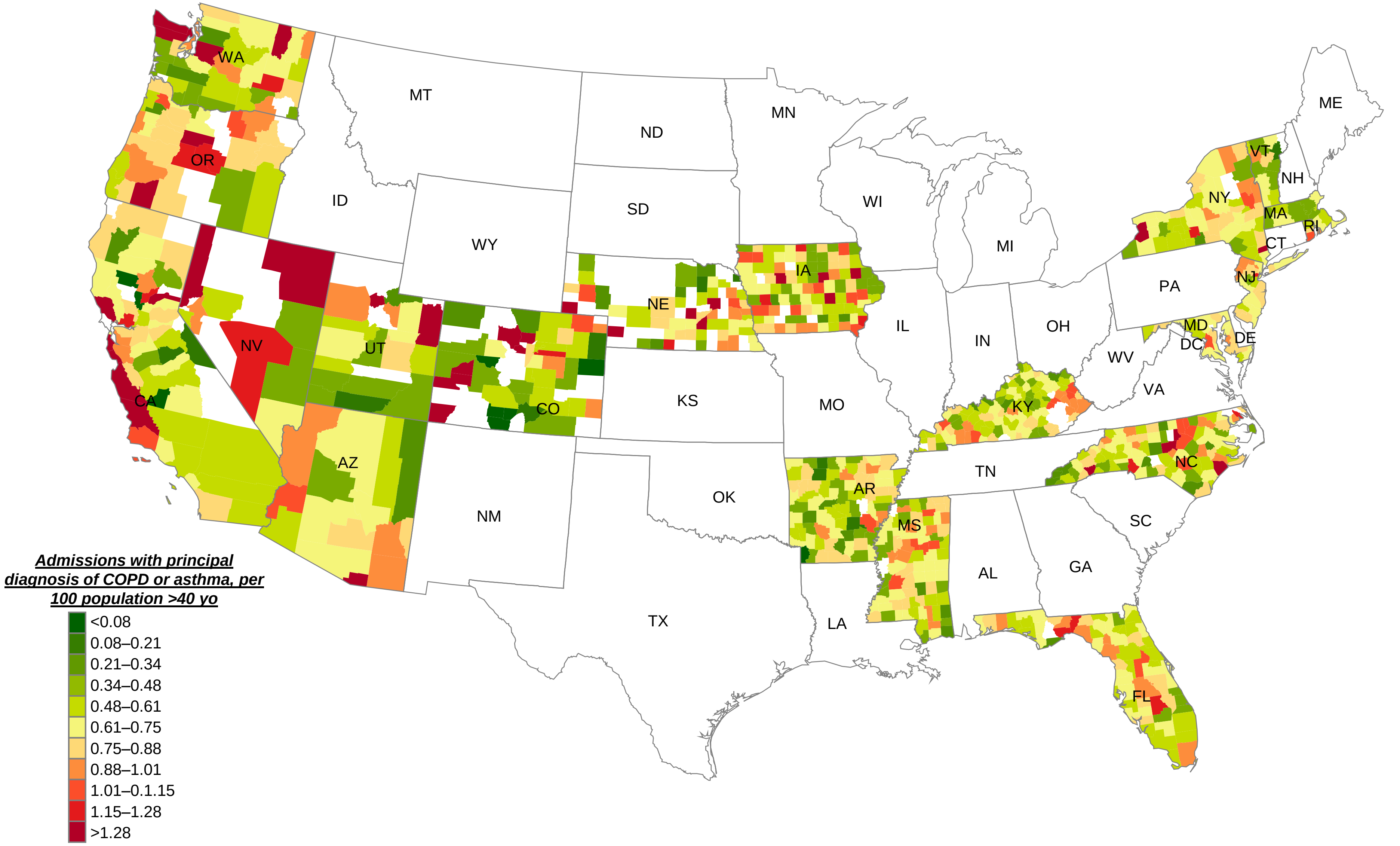

Copyright © 2014 by The Boston Consulting Group, Inc. All rights reserved.

Figure F: State level geographic variability in PQI05 - COPD or asthma in older adults admission rate

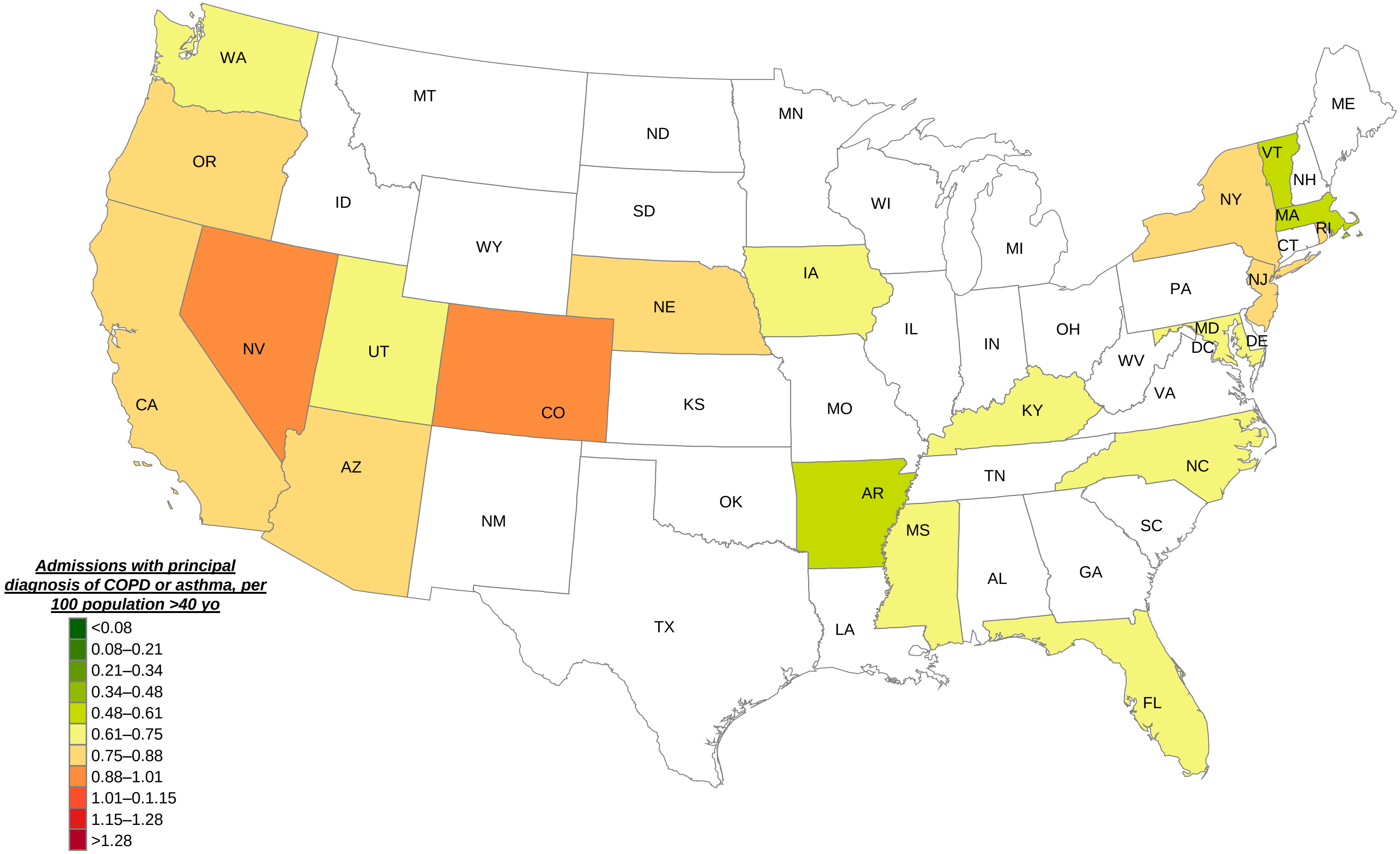

Copyright © 2014 by The Boston Consulting Group, Inc. All rights reserved.

Figure F: County level geographic variability in PQI08 - Congestive heart failure (CHF) admission rate

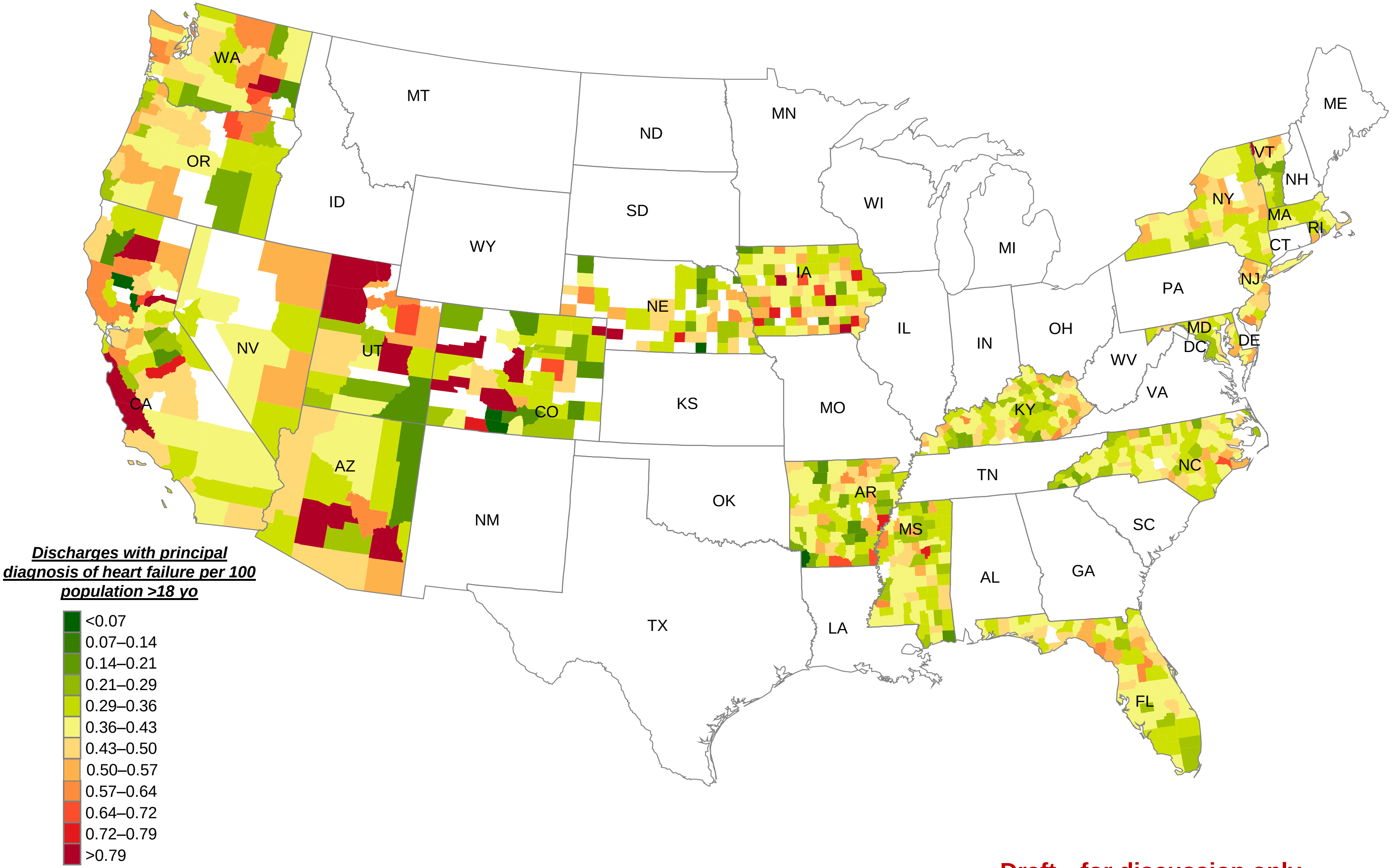

Copyright © 2014 by The Boston Consulting Group, Inc. All rights reserved.

Draft—for discussion only

Figure F: State level geographic variability in PQI08 - Congestive heart failure (CHF) admission rate

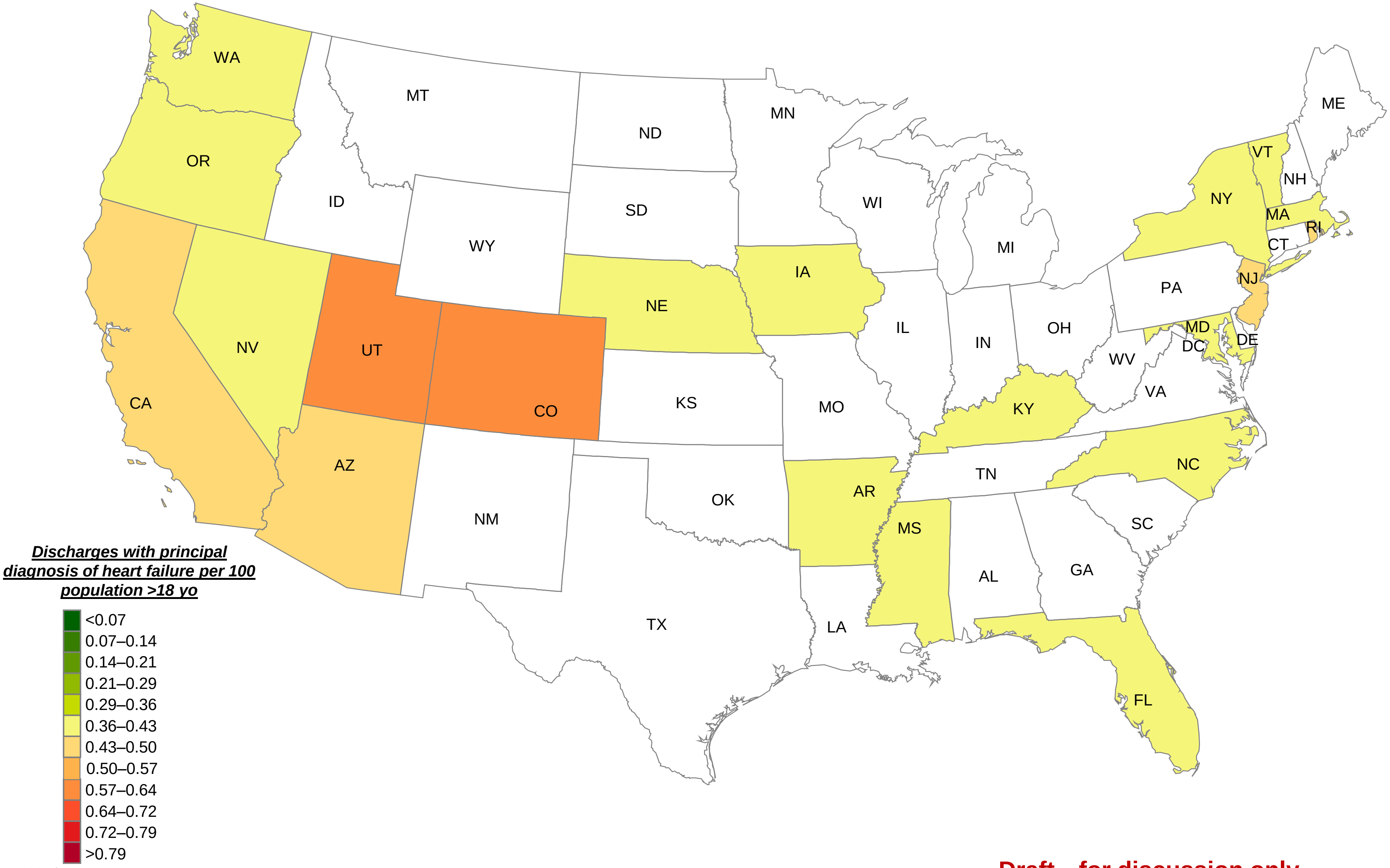

Copyright © 2014 by The Boston Consulting Group, Inc. All rights reserved.

Draft—for discussion only

Figure F: County level geographic variability in PQI10 - Dehydration admission rate

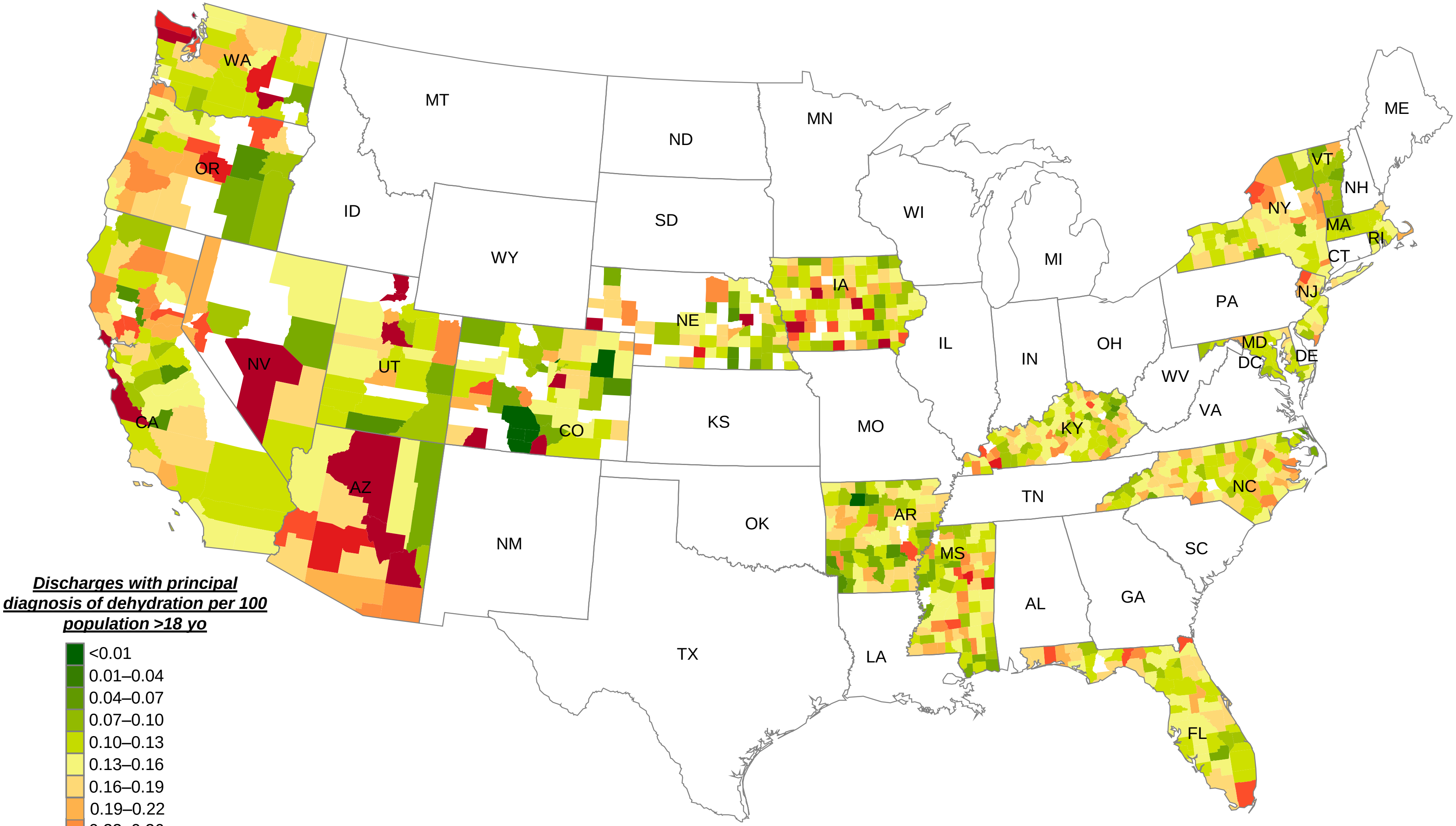

Copyright © 2014 by The Boston Consulting Group, Inc. All rights reserved.

Figure F: State level geographic variability in PQI10 - Dehydration admission rate

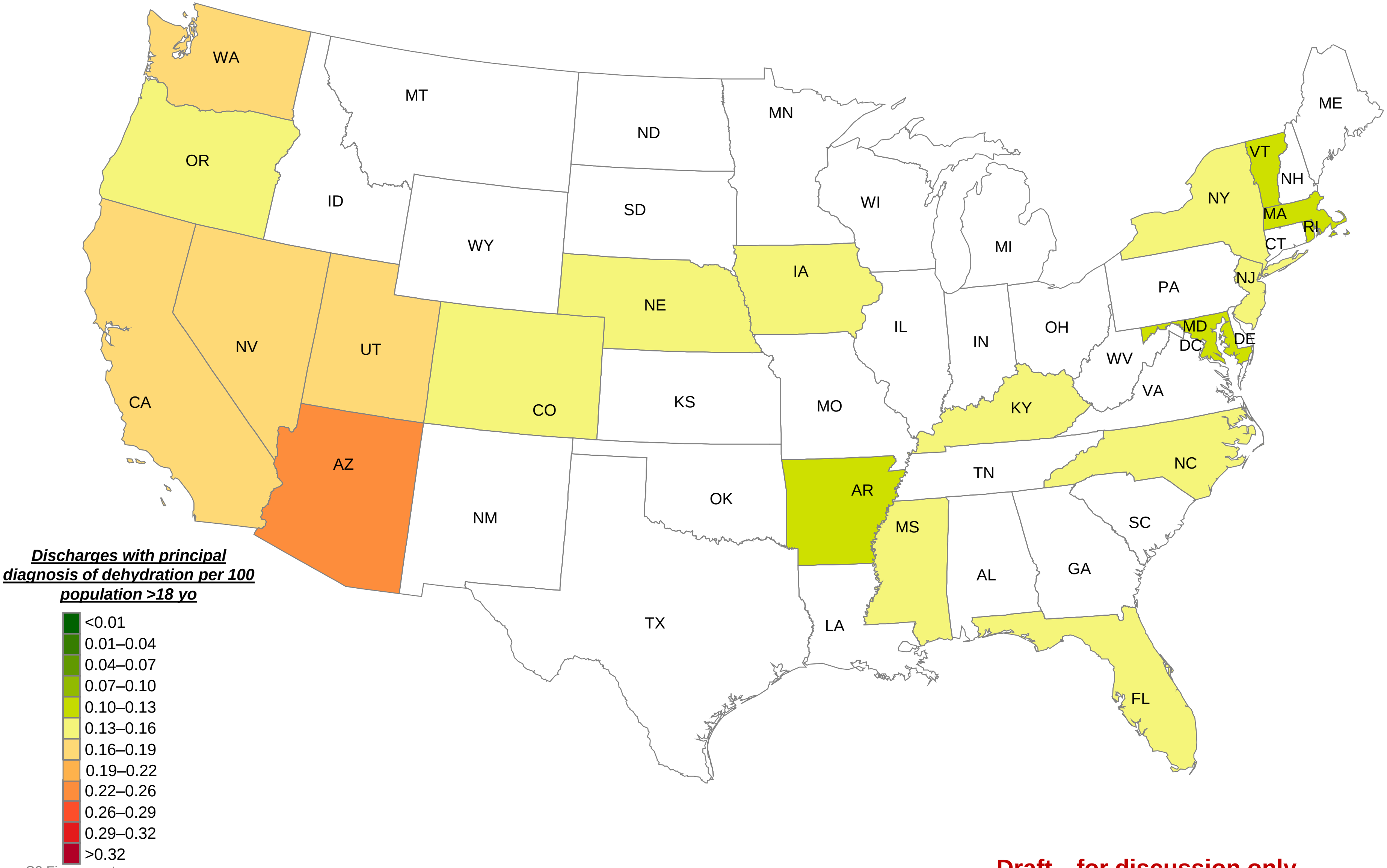

Copyright © 2014 by The Boston Consulting Group, Inc. All rights reserved.

Draft—for discussion only

Figure F: County level geographic variability in PQI11 - Bacterial pneumonia admission rate

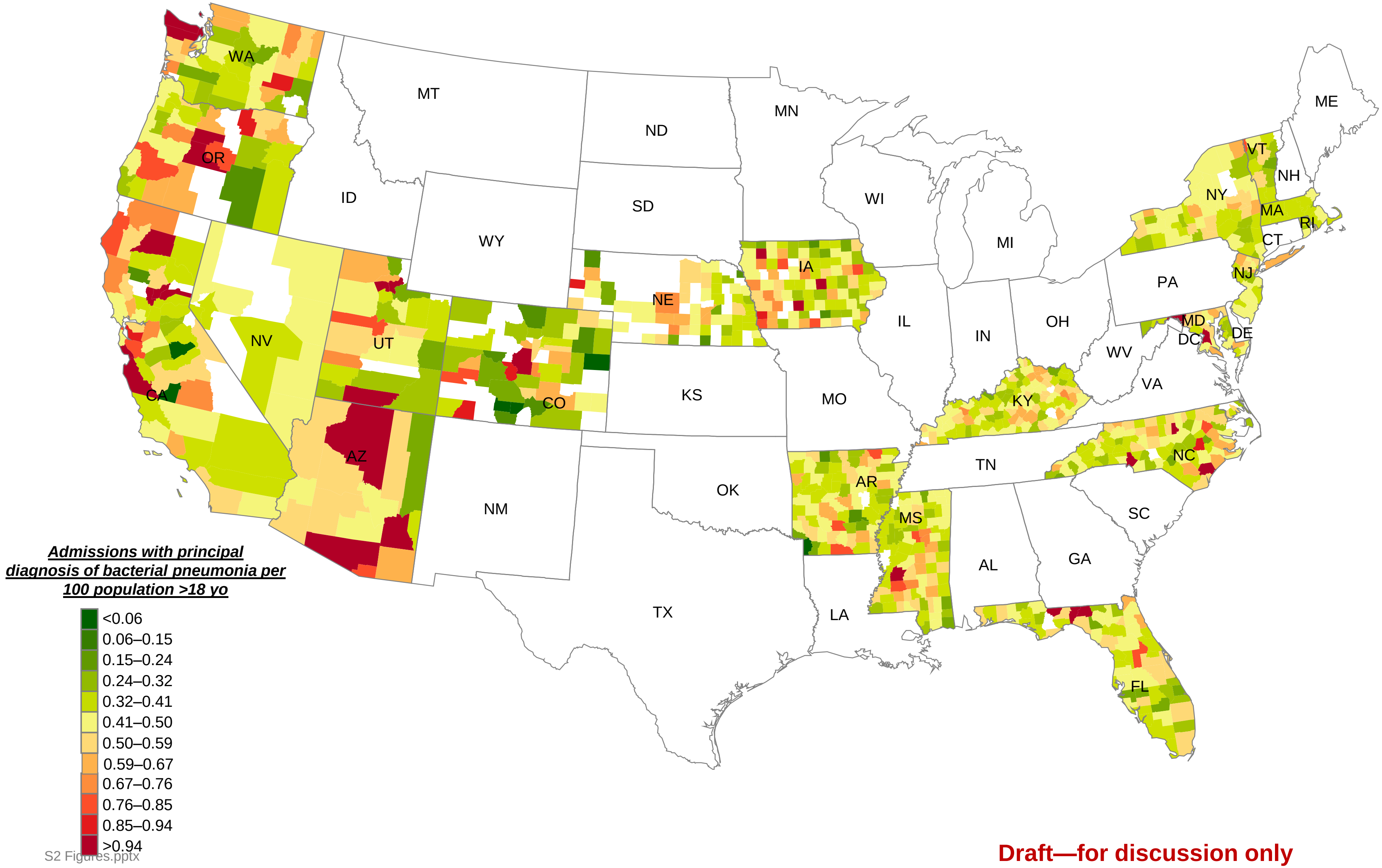

Copyright © 2014 by The Boston Consulting Group, Inc. All rights reserved.

Draft—for discussion only

Figure F: State level geographic variability in PQI11 - Bacterial pneumonia admission rate

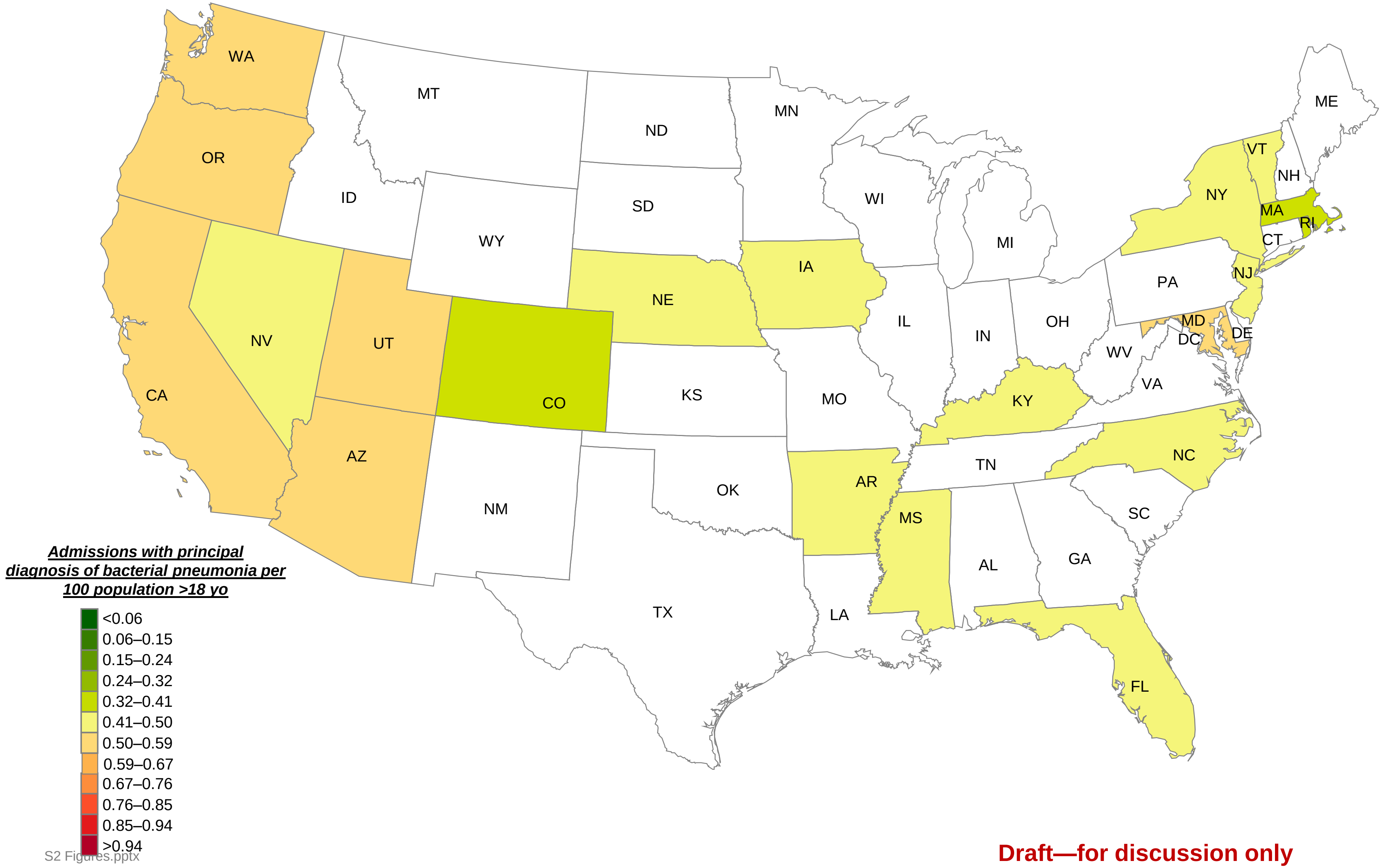

Copyright © 2014 by The Boston Consulting Group, Inc. All rights reserved.

Figure F: County level geographic variability in PQI12 - Urinary tract infection admission rate

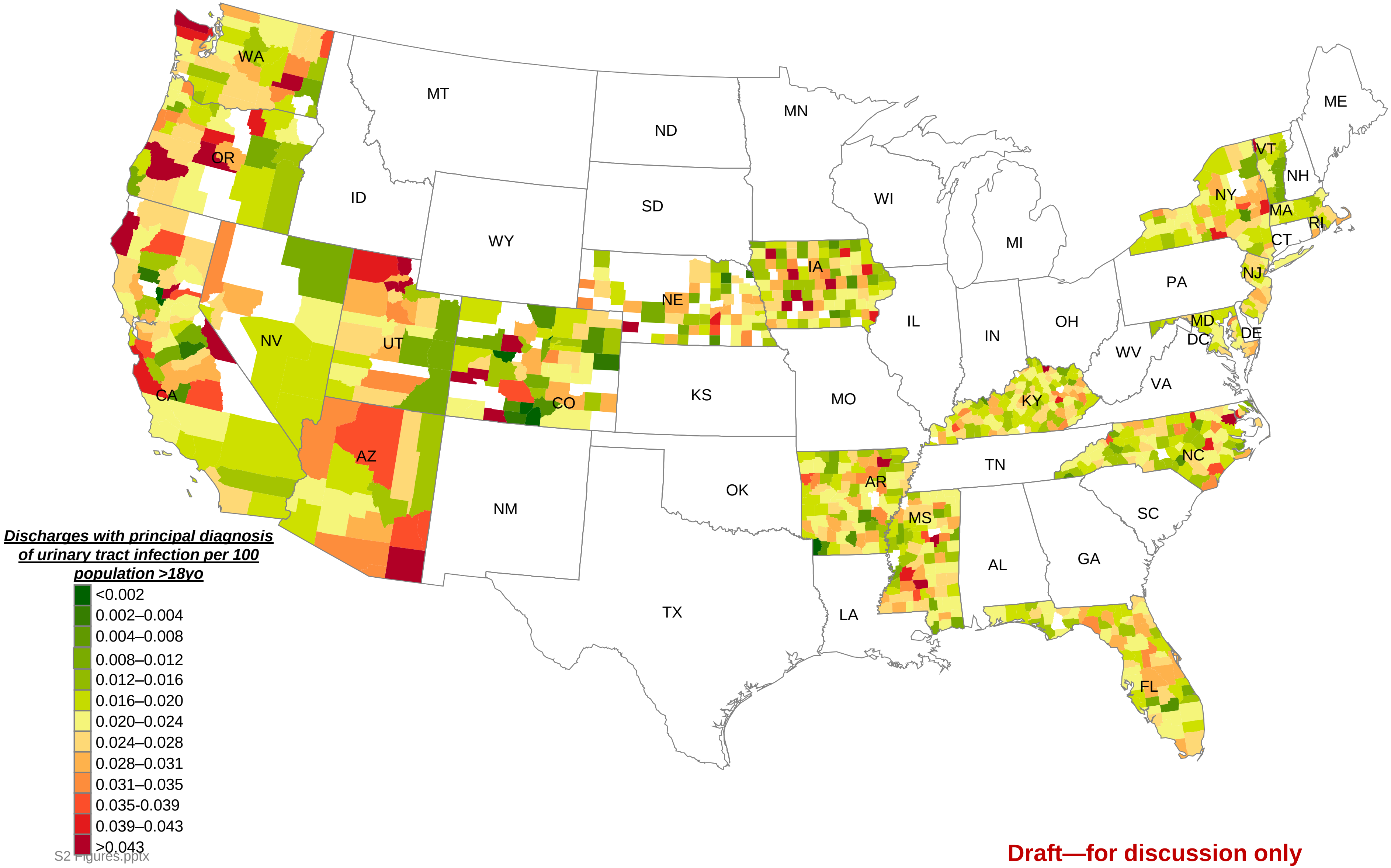

Copyright © 2014 by The Boston Consulting Group, Inc. All rights reserved.

Draft—for discussion only

Figure F: State level geographic variability in PQI12 - Urinary tract infection admission rate

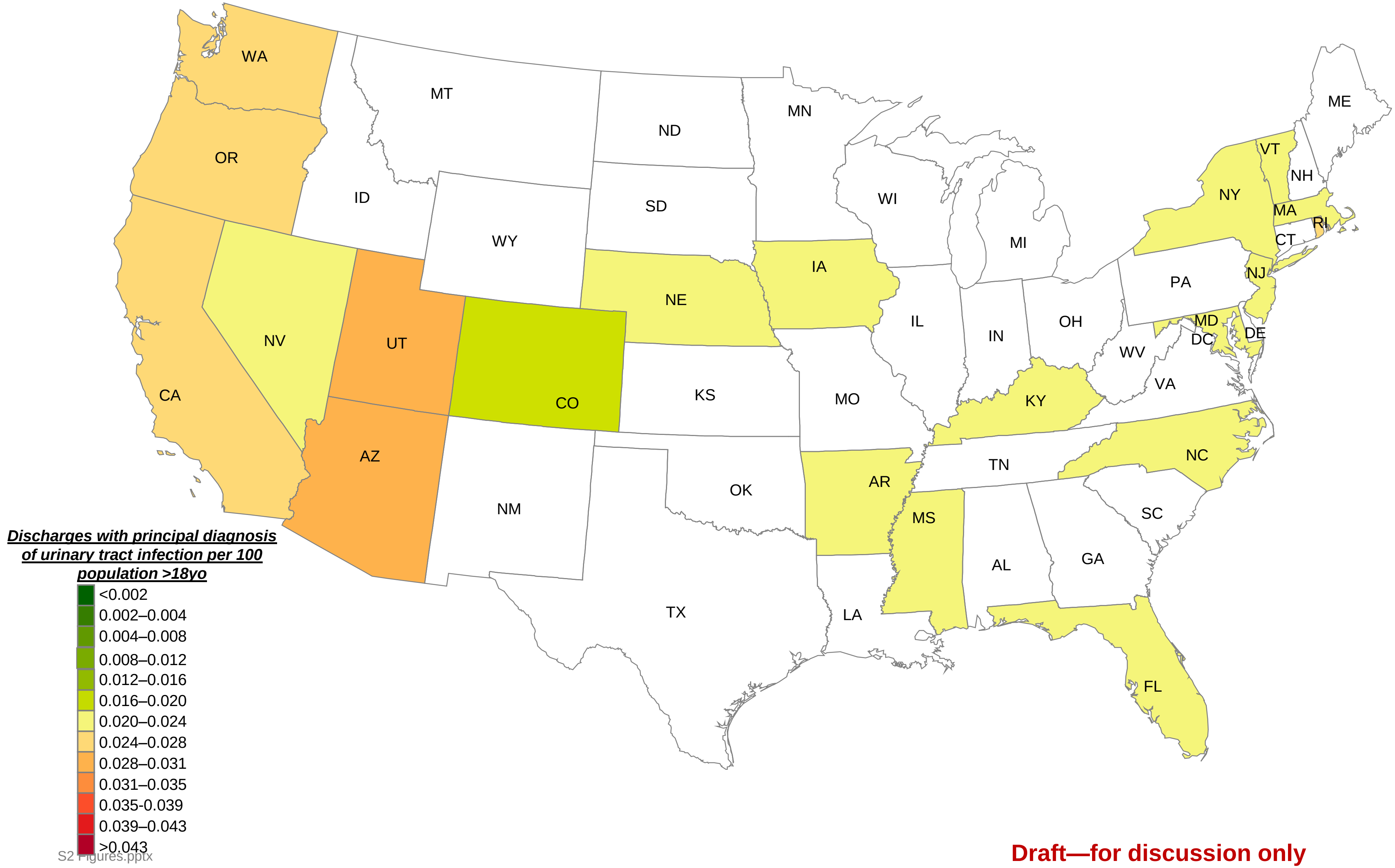

Copyright © 2014 by The Boston Consulting Group, Inc. All rights reserved.

Draft—for discussion only

Figure F: County level geographic variability in PQI14 - Uncontrolled diabetes admission rate

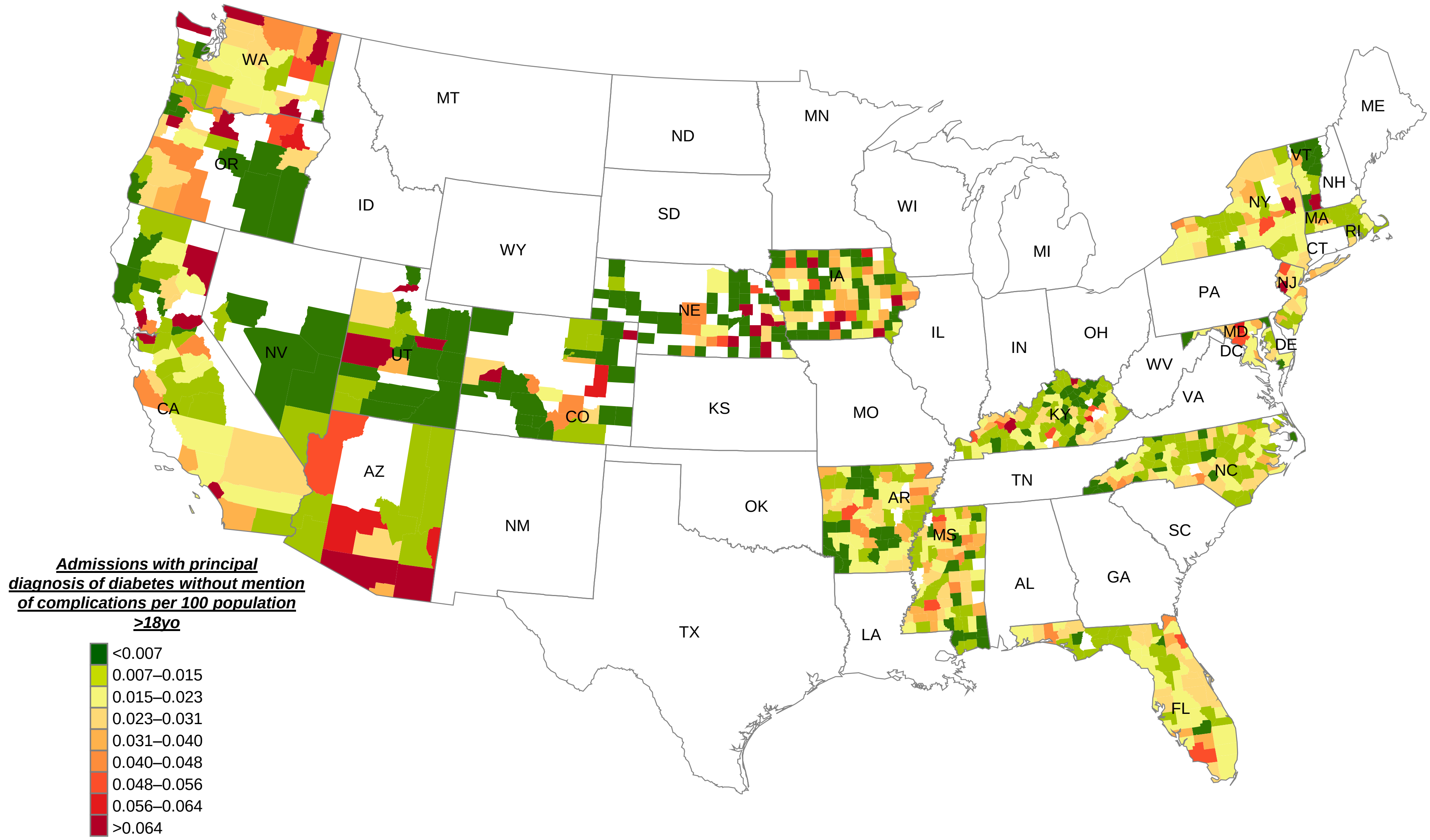

Copyright © 2014 by The Boston Consulting Group, Inc. All rights reserved.

Draft—for discussion only

Figure F: State level geographic variability in PQI14 - Uncontrolled diabetes admission rate

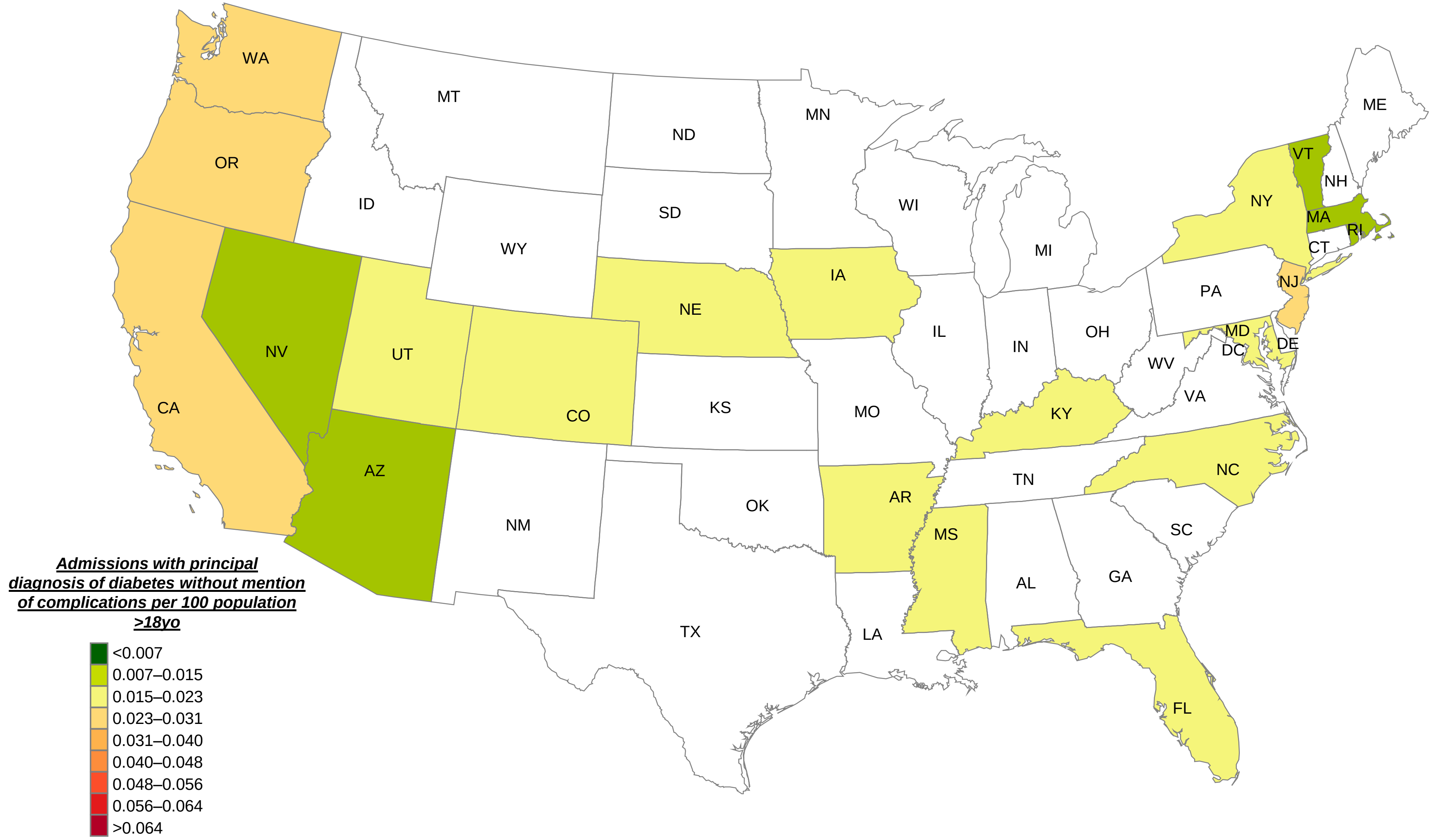

Copyright © 2014 by The Boston Consulting Group, Inc. All rights reserved.

Figure F: County level geographic variability in PQI15 - Asthma in younger adults admission rate

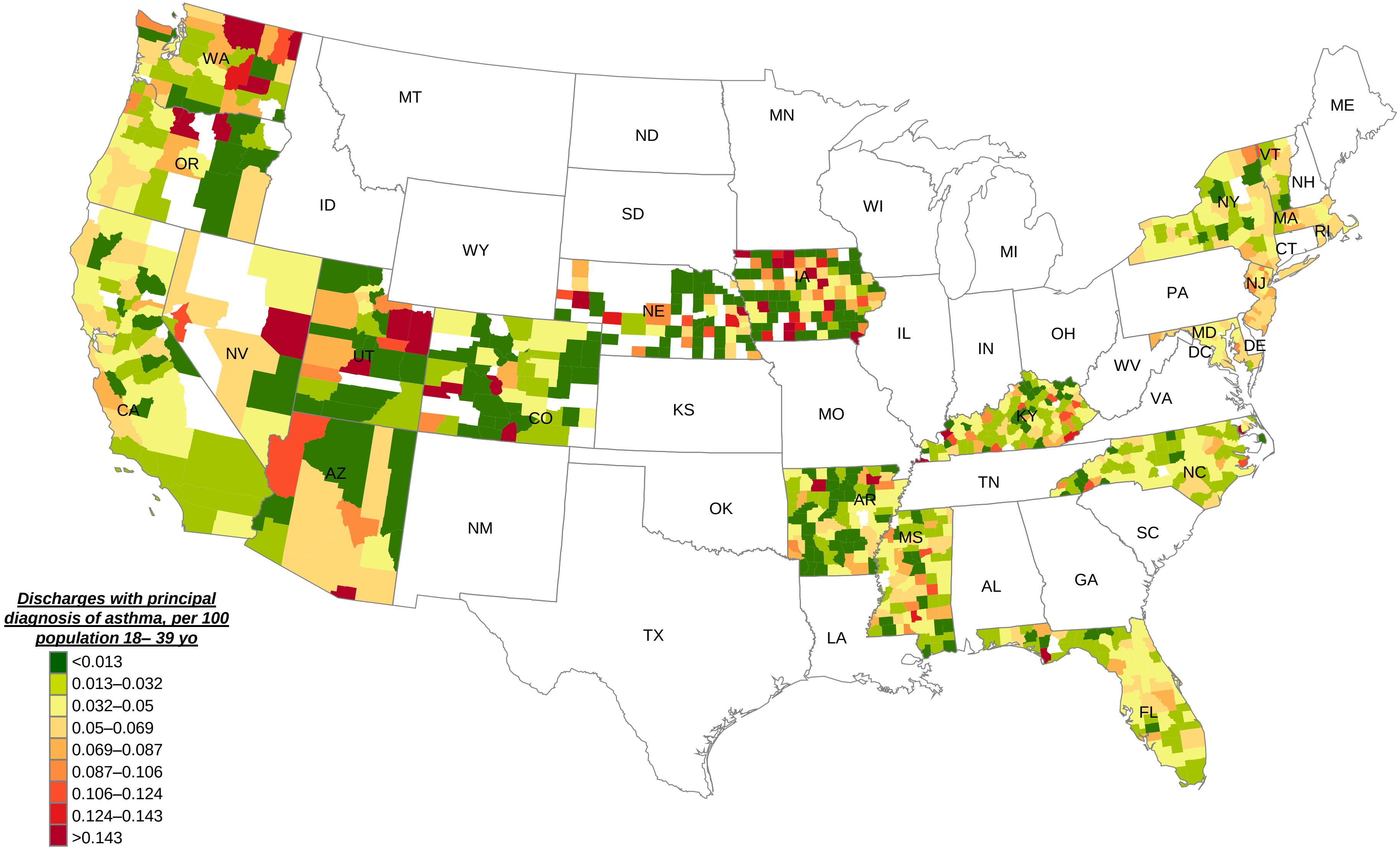

Copyright © 2014 by The Boston Consulting Group, Inc. All rights reserved.

Figure F: State level geographic variability in PQI15 - Asthma in younger adults admission rate

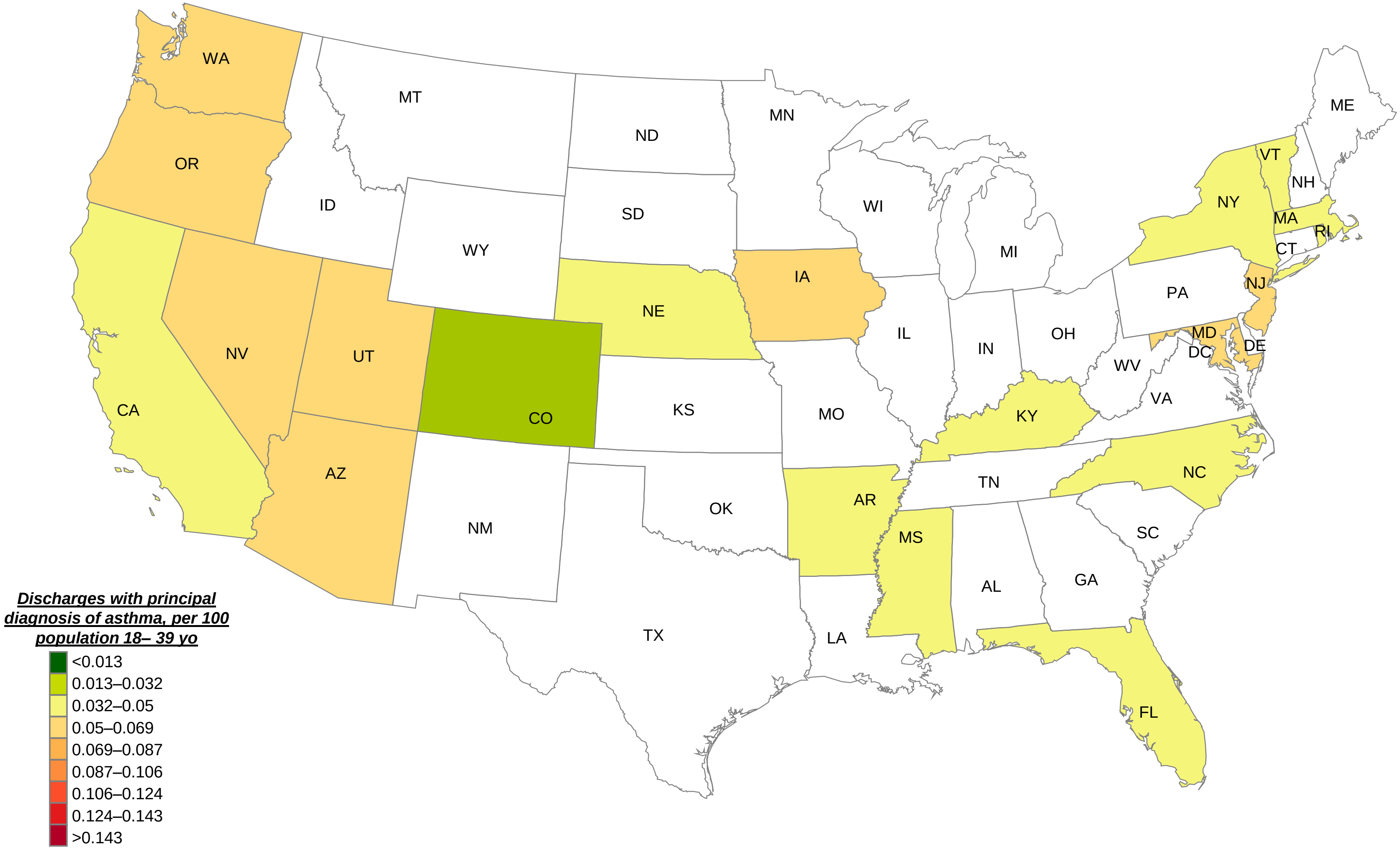

Copyright © 2014 by The Boston Consulting Group, Inc. All rights reserved.

Figure F: County level geographic variability in PQI16 - Lower-extremity amputation among diabetics

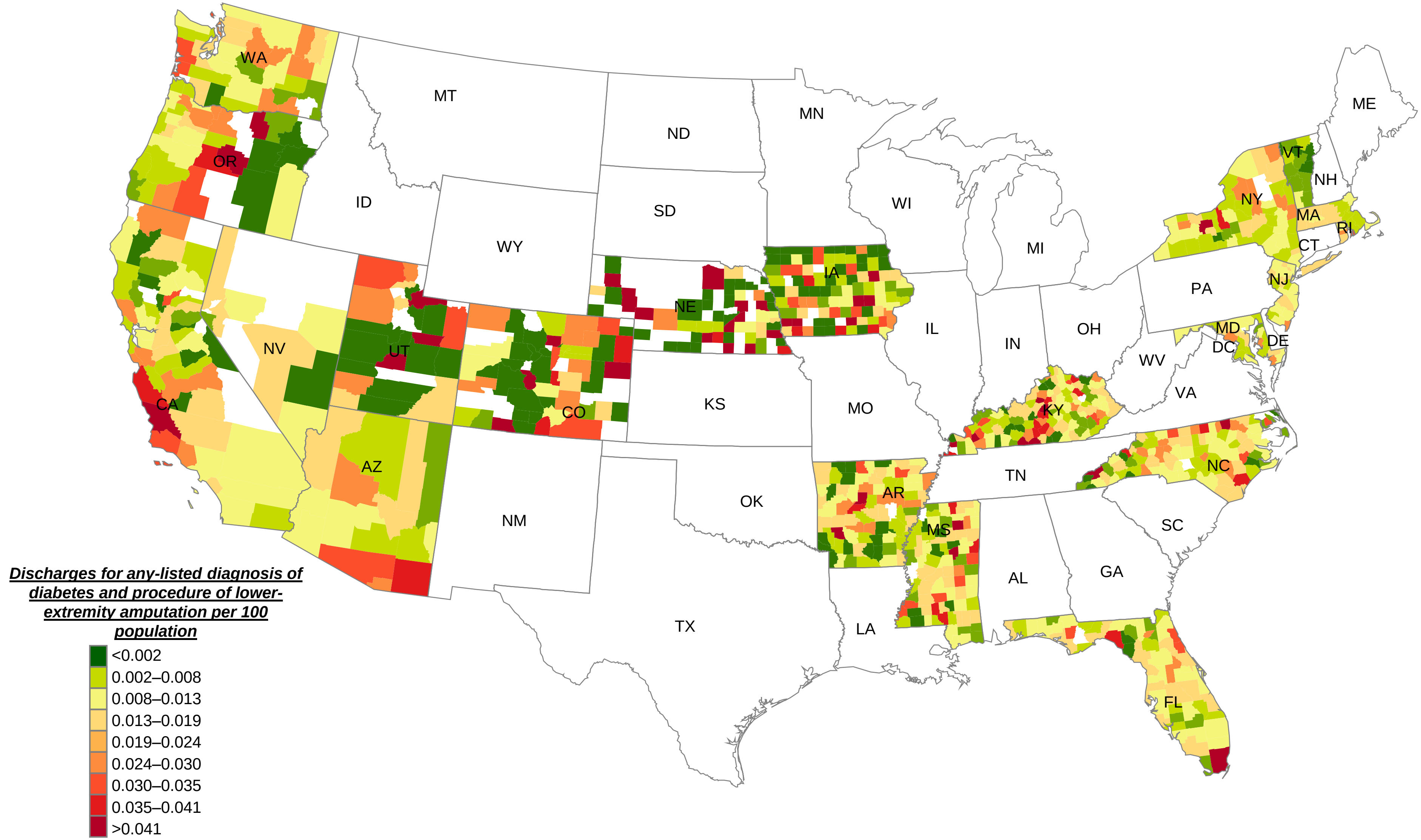

Copyright © 2014 by The Boston Consulting Group, Inc. All rights reserved.

Draft—for discussion only

Figure F: State level geographic variability in PQI16 - Lower-extremity amputation among diabetics

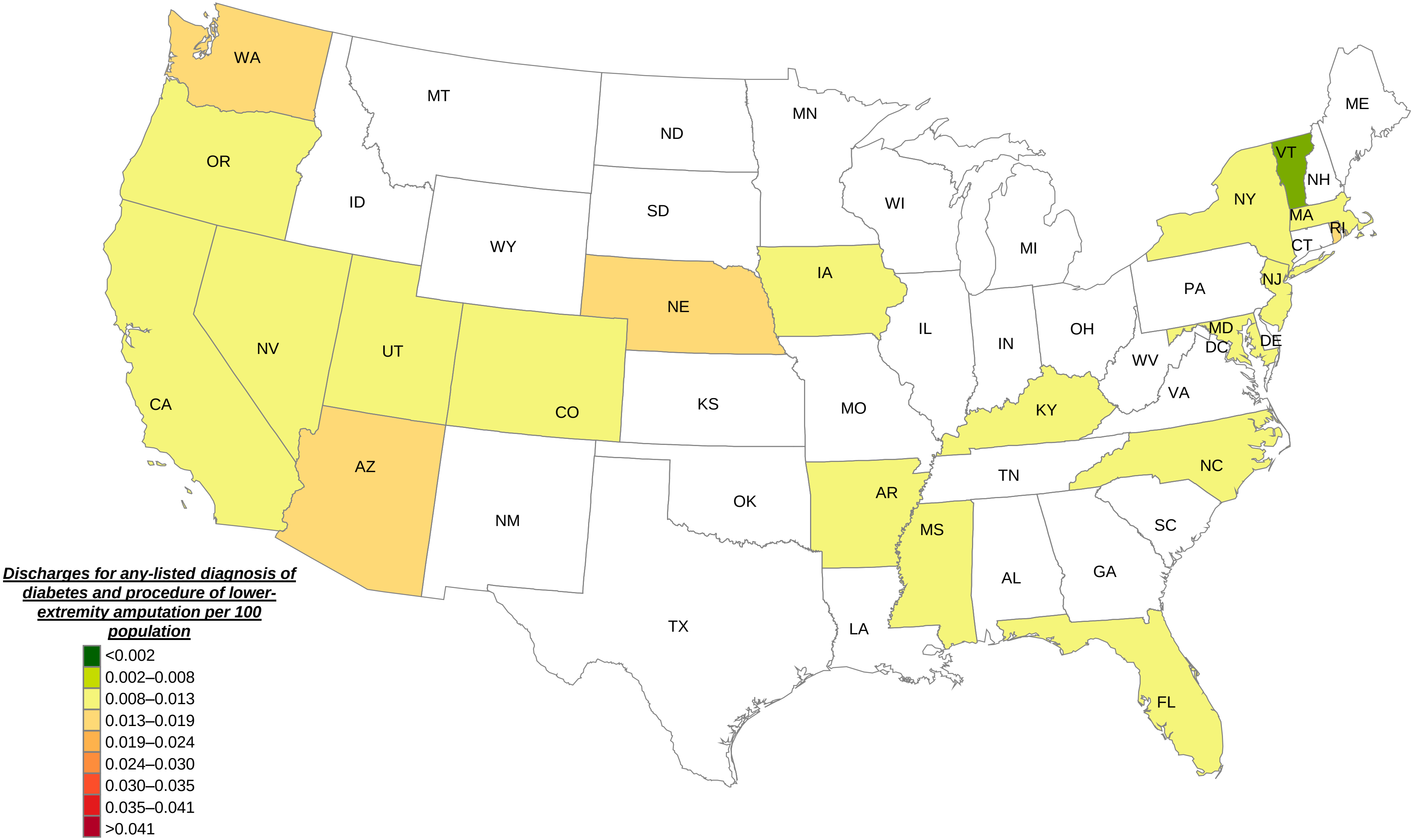

Copyright © 2014 by The Boston Consulting Group, Inc. All rights reserved.

Figure F: Representative map of geographic variability in outcomes across the US

This map shows geographic variability in each of the 24 outcomes studied. Large variation is observed in most outcomes. All values on the map are adjusted for low-volume noise using empirical Bayesian shrinkage method. Additionally, all HSAs with only one hospital were merged with adjacent HSA so that the resulting region contains two hospitals-as required by HCUP's data use agreement.

Copyright © 2014 by The Boston Consulting Group, Inc. All rights reserved.

Figure G:
Persistence of hospital/county performance over 11-years

Inpatient mortality

Outcome	Persistence
**IQI 15**	81%
**IQI 16**	72%
**IQI 17**	72%
**IQI 18**	58%
**IQI 19**	64%
**IQI 20**	69%
**AVG.**	69%

Inpatient safety

Outcome	Persistence
**PSI 03**	70%
**PSI 06**	66%
**PSI 07**	71%
**PSI 08**	49%
**PSI 12**	74%
**PSI 13**	62%
**PSI 14**	61%
**PSI 15**	84%
**AVG.**	67%

Prevention

Outcome	Persistence
**PQI 01**	82%
**PQI 03**	87%
**PQI 05**	94%
**PQI 10**	85%
**PQI 11**	93%
**PQI 12**	83%
**PQI 14**	83%
**PQI 15**	78%
**PQI 16**	74%
**AVG.**	84%

Copyright © 2014 by The Boston Consulting Group, Inc. All rights reserved.

Draft—for discussion only

Figure G: Footnote and methods
Persistence of hospital/county performance over 11-years

All outcomes measures show a high degree of persistence. Inpatient mortality has 69% persistence, inpatient safety has 67% persistence, and prevention has 85% persistence. To calculate, inpatient mortality and inpatient safety measures were first shrunk using Bayesian shrinkage. Then the variation each year was assessed by calculating Top 10%/bottom 10% ratio. Persistence in hospital performance was evaluated by ranking each hospital every year into deciles, as well as ranking each hospital based on its 11-year cumulative performance. Percent of time (years) in which a hospital was within two deciles of its 11-year rank was defined as persistence.

Copyright © 2014 by The Boston Consulting Group, Inc. All rights reserved.
