# Supplementary material for: Quantifying Geographic Variation in Health Care Outcomes in the United States before and after Risk-Adjustment
Source: PLoS One. 2016 Dec 14;11(12):e0166762. doi: 10.1371/journal.pone.0166762 (PMC5156342; doi:10.1371/journal.pone.0166762)
Supplement: S2 File — (PDF) [file pone.0166762.s002.pdf]

A: Volume-outcome relationship

Methods

For mortality we assessed the relationship between outcomes and case volume by modeling the mortality (M) as $M=-\alpha * \ln(V)+\beta$, where V is hospital case volume, and α and β are constants for each of the inpatient mortality outcomes. For inpatient safety, the sample size was too small to establish a significant volume-outcome relationship. For prevention measures, the volume-outcome analysis is not meaningful because the prevention outcomes measure is admissions rates. The denominator of the ratio is the total population in a given geography; not hospital case volume.

Results

For all mortality measures assessed, the relationship between outcomes and case volume is clear: low-case-volume hospitals have higher mortality (Figure 3). The coefficient of proportionality (α) was largest for AMI mortality (0.03) and smallest for GI hemorrhage (0.003). This translates into meaningful differences in performance between low-case-volume and high-case-volume hospitals. Consider acute myocardial infarction: for hospitals with 50 or fewer AMI cases, the average inpatient mortality rate is 13%, while for hospitals with more than 200 AMI cases it is 5% ($p<0.001$). The volume-outcome relationship is also observed in PSI 13 (postoperative sepsis rate). It may be present

in the other PSIs as well, but is difficult to confirm in this study due to the low overall incidence rate present in many inpatient safety measures.

B: Risk adjustment using Poisson distribution

As noted in the methods section, we repeated the risk adjustment using the Poisson distribution in order to examine the impact of the choice of distribution model on our conclusions.

After conducting the risk adjustment using a Poisson model we computed D9/D1 ratios with an identical methodology as we did previously with a Gaussian distribution. Nineteen out of twenty one measures examined with a Poisson model were within 25% of the D9/D1 value calculated with a Gaussian distribution. The consistency of this variation with both Gaussian and Poisson models further validates that there is substantial variation in outcomes between hospitals and counties even after risk adjustment.

Two out of the twenty-one measures we examined had more than a 25% variation between methodologies. PSI 3 shows a similar D9/D1 ratio after adjusting for population factors (4% difference) but shows meaningful differences between the Gaussian and Poisson models after adjustment for population factors and co-morbidities (27% difference) as well as after adjustment for population factors, co-morbidities and system factors (42% difference). The discrepancy is likely a result of significant under-dispersion in the PSI count data as indicated by a Cameron & Trivedi[1] test (c value for PSI03 <-100 while for all the other PSIs the values are between -4 and 0). PSI 08 shows similar D9/D1 ratios after adjusting for population factors (6% difference) but shows meaningful difference between Gaussian vs. Poisson model after adjustment for population factors

and co-morbidities (51% difference) as well as after adjustment for population factors, co-morbidities and system factors (57% difference). In this case it is likely that the discrepancy comes from the fact that post-operative hip fracture is very uncommon and only observed in ~5% of the hospitals.

C: Longitudinal analysis

Methods

To understand the variation in hospital performance over time and quantify the persistence in performance, we analyzed inpatient mortality, inpatient safety, and prevention outcomes at ~250 hospitals and ~60 counties in New York state over an 11-year period from 2002 to 2012. Inpatient mortality and inpatient safety measures were first shrunk using Bayesian shrinkage as described previously. The variation each year was assessed by calculating the top 10%/bottom 10% ratio, and variation over the entire 11-year period was assessed by first calculating weighted average performance of each hospital over the 11-year period and then calculating the top 10%/bottom 10% ratio. Persistence in hospital performance was evaluated by ranking each hospital or county every year in deciles as well as ranking them based on their 11-year performance. The percent of time (years) in which the hospital was within two deciles of its 11-year rank was defined as its persistence (e.g., a hospital whose annual rank was always within two deciles of its 11-year rank would have 100% persistence).

Results

Longitudinal analysis demonstrated that hospital performance showed similar large levels of variation each year during the 11-year period and when data was aggregated over the entire period. For

example, for AMI mortality (IQI 15) the annual top 10%/bottom 10% mortality ratio ranged from 3.4 to 4.2, and the 11-year weighted average of each hospital results in a D9/D1 ratio of 3.9. Hospitals have an average persistence of ~70% across all inpatient mortality and safety measures and counties have ~85% persistence across all prevention outcomes (Figure G in S1 file). This is consistent with a similar study looking into the predictive power of hospital rankings for CABG mortality, which showed that two-year ranking is a strong predictor of future performance[2]. This suggests that the observed national variability is not unique to 2011.

References:

1. Cameron AC, Trivedi PK. Regression-based tests for overdispersion in the Poisson model. J Econom. 1990;46: 347–364.
2. Glance LG, Dick AW, Mukamel DB, Li Y, Osler TM. How well do hospital mortality rates reported in the New York State CABG report card predict subsequent hospital performance? Med Care. 2010;48: 466–471.
